# In vivo hippocampal subfield volumes in bipolar disorder—A mega‐analysis from The Enhancing Neuro Imaging Genetics through Meta‐Analysis Bipolar Disorder Working Group

**DOI:** 10.1002/hbm.25249

**Published:** 2020-10-19

**Authors:** Unn K. Haukvik, Tiril P. Gurholt, Stener Nerland, Torbjørn Elvsåshagen, Theophilus N. Akudjedu, Martin Alda, Dag Alnæs, Silvia Alonso‐Lana, Jochen Bauer, Bernhard T. Baune, Francesco Benedetti, Michael Berk, Francesco Bettella, Erlend Bøen, Caterina M. Bonnín, Paolo Brambilla, Erick J. Canales‐Rodríguez, Dara M. Cannon, Xavier Caseras, Orwa Dandash, Udo Dannlowski, Giuseppe Delvecchio, Ana M. Díaz‐Zuluaga, Theo G. M. van Erp, Mar Fatjó‐Vilas, Sonya F. Foley, Katharina Förster, Janice M. Fullerton, José M. Goikolea, Dominik Grotegerd, Oliver Gruber, Bartholomeus C. M. Haarman, Beathe Haatveit, Tomas Hajek, Brian Hallahan, Mathew Harris, Emma L. Hawkins, Fleur M. Howells, Carina Hülsmann, Neda Jahanshad, Kjetil N. Jørgensen, Tilo Kircher, Bernd Krämer, Axel Krug, Rayus Kuplicki, Trine V. Lagerberg, Thomas M. Lancaster, Rhoshel K. Lenroot, Vera Lonning, Carlos López‐Jaramillo, Ulrik F. Malt, Colm McDonald, Andrew M. McIntosh, Genevieve McPhilemy, Dennis van der Meer, Ingrid Melle, Elisa M. T. Melloni, Philip B. Mitchell, Leila Nabulsi, Igor Nenadić, Viola Oertel, Lucio Oldani, Nils Opel, Maria C. G. Otaduy, Bronwyn J. Overs, Julian A. Pineda‐Zapata, Edith Pomarol‐Clotet, Joaquim Radua, Lisa Rauer, Ronny Redlich, Jonathan Repple, Maria M. Rive, Gloria Roberts, Henricus G. Ruhe, Lauren E. Salminen, Raymond Salvador, Salvador Sarró, Jonathan Savitz, Aart H. Schene, Kang Sim, Marcio G. Soeiro‐de‐Souza, Michael Stäblein, Dan J. Stein, Frederike Stein, Christian K. Tamnes, Henk S. Temmingh, Sophia I. Thomopoulos, Dick J. Veltman, Eduard Vieta, Lena Waltemate, Lars T. Westlye, Heather C. Whalley, Philipp G. Sämann, Paul M. Thompson, Christopher R. K. Ching, Ole A. Andreassen, Ingrid Agartz, ENIGMA Bipolar Disorder Working Group

**Affiliations:** ^1^ Department of Adult Mental Health Institute of Clinical Medicine, University of Oslo Oslo Norway; ^2^ Norwegian Centre for Mental Disorders Research (NORMENT) Division of Mental Health and Addiction, Oslo University Hospital Oslo Norway; ^3^ Norwegian Centre for Mental Disorders Research (NORMENT) Institute of Clinical Medicine, University of Oslo Oslo Norway; ^4^ Department of Psychiatric Research Diakonhjemmet Hospital Oslo Norway; ^5^ Department of Neurology Oslo University Hospital Oslo Norway; ^6^ Institute of Clinical Medicine University of Oslo Oslo Norway; ^7^ Centre for Neuroimaging & Cognitive Genomics (NICOG) Clinical Neuroimaging Laboratory, NCBES Galway Neuroscience Centre, College of Medicine Nursing and Health Sciences, National University of Ireland Galway Galway Ireland; ^8^ Institute of Medical Imaging & Visualisation Faculty of Health & Social Sciences, Bournemouth University Bournemouth UK; ^9^ Department of Psychiatry Dalhousie University Halifax Nova Scotia Canada; ^10^ National Institute of Mental Health Klecany Czech Republic; ^11^ FIDMAG Germanes Hospitalàries Research Foundation CIBERSAM Barcelona Spain; ^12^ Institute of Clinical Radiology University of Münster Münster Germany; ^13^ Department of Psychiatry University of Münster Münster Germany; ^14^ Department of Psychiatry Melbourne Medical School, The University of Melbourne Melbourne Australia; ^15^ The Florey Institute of Neuroscience and Mental Health The University of Melbourne Parkville Australia; ^16^ Psychiatry and Clinical Psychobiology Scientific Institute Ospedale San Raffaele Milan Italy; ^17^ University Vita‐Salute San Raffaele Milan Italy; ^18^ Deakin University IMPACT, the Institute for Mental and Physical Health and Clinical Translation, School of Medicine, Barwon Health Geelong Victoria Australia; ^19^ Orygen, The National Centre of Excellence in Youth Mental Health and Centre for Youth Mental Health, the Department of Psychiatry and the Florey Institute for Neuroscience and Mental Health The University of Melbourne Melbourne Victoria Australia; ^20^ Psychosomatic and CL Psychiatry Division of Mental Health and Addiction, Oslo University Hospital Oslo Norway; ^21^ Barcelona Bipolar Disorders and Depressive Unit Hospital Clinic, Institute of Neurosciences, University of Barcelona, IDIBAPS, CIBERSAM Barcelona Spain; ^22^ Department of Neurosciences and Mental Health Fondazione IRCCS Ca' Granda Ospedale Maggiore Policlinico Milan Italy; ^23^ Department of Pathophysiology and Transplantation University of Milan Milan Italy; ^24^ MRC Centre for Neuropsychiatric Genetics and Genomics, Department of Psychological Medicine and Clinical Neurosciences Cardiff University Cardiff UK; ^25^ Brain, Mind and Society Research Hub, Turner Institute for Brain and Mental Health, School of Psychological Sciences Monash University Clayton Victoria Australia; ^26^ Melbourne Neuropsychiatry Centre, Department of Psychiatry University of Melbourne and Melbourne Health Melbourne Victoria Australia; ^27^ Research Group in Psychiatry, Department of Psychiatry Faculty of Medicine, Universidad de Antioquia Medellín Antioquia Colombia; ^28^ Clinical Translational Neuroscience Laboratory, Department of Psychiatry and Human Behavior University of California Irvine Irvine California USA; ^29^ Center for the Neurobiology of Learning University of California Irvine and Memory Irvine California USA; ^30^ Cardiff University Brain Research Imaging Centre (CUBRIC) Cardiff University Cardiff UK; ^31^ Neuroscience Research Australia Randwick New South Wales Australia; ^32^ School of Medical Sciences The University of New South Wales Sydney New South Wales Australia; ^33^ Section for Experimental Psychopathology and Neuroimaging, Department of General Psychiatry Heidelberg University Hospital Heidelberg Germany; ^34^ Department of Psychiatry University Medical Center Groningen, University of Groningen Groningen The Netherlands; ^35^ Division of Psychiatry University of Edinburgh Edinburgh UK; ^36^ Department of Psychiatry and Mental Health University of Cape Town Cape Town Western Cape South Africa; ^37^ Neuroscience Institute University of Cape Town Cape Town Western Cape South Africa; ^38^ Imaging Genetics Center USC Mark and Mary Stevens Neuroimaging and Informatics Institute, Keck School of Medicine of the University of Southern California Marina del Rey California USA; ^39^ Department of Psychiatry and Psychotherapy Philipps‐University Marburg Marburg Germany; ^40^ Center for Mind Brain and Behavior (CMBB) Marburg Germany; ^41^ Department of Psychiatry and Psychotherapy University of Bonn Bonn Germany; ^42^ Laureate Institute for Brain Research Tulsa Oklahoma USA; ^43^ School of Psychology Bath University Bath UK; ^44^ School of Psychiatry University of New South Wales Sydney New South Wales Australia; ^45^ University of New Mexico Albuquerque New Mexico USA; ^46^ Mood Disorders Program Hospital Universitario San Vicente Fundación Medellín Antioquia Colombia; ^47^ School of Mental Health and Neuroscience Faculty of Health, Medicine and Life Sciences, Maastricht University Maastricht The Netherlands; ^48^ Black Dog Institute Sydney New South Wales Australia; ^49^ Department of Psychiatry Psychosomatic Medicine and Psychotherapy, University Hospital Frankfurt Frankfurt am Main Germany; ^50^ LIM44, Department of Radiology and Oncology University of São Paulo São Paulo Brazil; ^51^ Research Group Instituto de Alta Tecnología Médica Medellín Antioquia Colombia; ^52^ Institut d'Investigacions Biomèdiques August Pi i Sunyer (IDIBAPS), CIBERSAM Barcelona Spain; ^53^ Department of Psychosis Studies Institute of Psychiatry, Psychology and Neuroscience, King's College London London UK; ^54^ Department of Clinical Neuroscience Centre for Psychiatry Research, Karolinska Institutet Stockholm Sweden; ^55^ Psychiatry Amsterdam UMC, Location AMC Amsterdam The Netherlands; ^56^ Donders Institute for Brain, Cognition and Behavior Radboud University Nijmegen The Netherlands; ^57^ Department of Psychiatry Radboudumc Nijmegen The Netherlands; ^58^ Oxley College of Health Sciences The University of Tulsa Tulsa Oklahoma USA; ^59^ West Region/Institute of Mental Health Singapore Singapore; ^60^ Yong Loo Lin School of Medicine/National University of Singapore Singapore Singapore; ^61^ Lee Kong Chian School of Medicine/Nanyang Technological University Singapore Singapore; ^62^ Department of Psychiatry Universidade de São Paulo São Paulo Brazil; ^63^ SA MRC Unit on Risk & Resilience in Mental Disorders, Department of Psychiatry & Neuroscience Institute University of Cape Town Cape Town Western Cape South Africa; ^64^ PROMENTA Research Center, Department of Psychology University of Oslo Oslo Norway; ^65^ General Adult Psychiatry Division Valkenberg Hospital Cape Town Western Cape South Africa; ^66^ Department of Psychiatry Amsterdam UMC, Location VUMC Amsterdam The Netherlands; ^67^ Amsterdam Neuroscience Amsterdam UMC Amsterdam The Netherlands; ^68^ Hospital Clinic University of Barcelona, IDIBAPS, CIBERSAM Barcelona Catalonia Spain; ^69^ Department of Psychology University of Oslo Oslo Norway; ^70^ Max Planck Institute of Psychiatry Munich Germany; ^71^ Department of Clinical Neuroscience Centre for Psychiatric Research, Karolinska Institutet Stockholm Sweden

**Keywords:** bipolar disorder subtype, hippocampus, large‐scale, lithium, psychosis, structural brain MRI

## Abstract

The hippocampus consists of anatomically and functionally distinct subfields that may be differentially involved in the pathophysiology of bipolar disorder (BD). Here we, the Enhancing NeuroImaging Genetics through Meta‐Analysis Bipolar Disorder workinggroup, study hippocampal subfield volumetry in BD. T1‐weighted magnetic resonance imaging scans from 4,698 individuals (BD = 1,472, healthy controls [HC] = 3,226) from 23 sites worldwide were processed with FreeSurfer. We used linear mixed‐effects models and mega‐analysis to investigate differences in hippocampal subfield volumes between BD and HC, followed by analyses of clinical characteristics and medication use. BD showed significantly smaller volumes of the whole hippocampus (Cohen's *d =* −0.20), cornu ammonis (CA)1 (*d =* −0.18), CA2/3 (*d =* −0.11), CA4 (*d =* −0.19), molecular layer (*d =* −0.21), granule cell layer of dentate gyrus (*d =* −0.21), hippocampal tail (*d =* −0.10), subiculum (*d =* −0.15), presubiculum (*d =* −0.18), and hippocampal amygdala transition area (*d =* −0.17) compared to HC. Lithium users did not show volume differences compared to HC, while non‐users did. Antipsychotics or antiepileptic use was associated with smaller volumes. In this largest study of hippocampal subfields in BD to date, we show widespread reductions in nine of 12 subfields studied. The associations were modulated by medication use and specifically the lack of differences between lithium users and HC supports a possible protective role of lithium in BD.

## Graphical abstract

- The hippocampus consists of anatomically and functionally distinct subfields that may be differentially involved in the pathophysiology of bipolar disorder.
- In the largest study of hippocampal subfields in bipolar disorder to date, from 23 sites worldwide, we report widespread reductions in nine of 12 subfields.
- The lack of differences between lithium users and healthy controls supports a possible protective role of lithium in bipolar disorder.

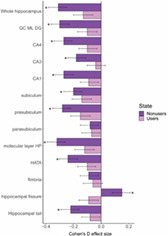

## 1 INTRODUCTION

Bipolar disorders (BD) affect over 1% of the population worldwide (Grande, Berk, Birmaher, & Vieta, 2016). Clinical characteristics and severity of the disorder vary; while some patients are disabled, others live normal lives between mood episodes; some experience psychotic episodes whereas others do not, and medication regimes and responses differ widely. This clinical heterogeneity may hamper the search for consistent underlying pathophysiological disease mechanisms that remain elusive despite widespread research efforts.

Smaller hippocampal volumes have been reported in BD compared to healthy controls (HC) in magnetic resonance imaging (MRI) studies (Haukvik et al., 2015; Hibar et al., 2016). The hippocampus is a key structure in the limbic system and is involved in multiple cognitive functions including pattern separation/completion processes that contribute to learning and episodic memory (Squire & Wixted, 2011), emotion regulation, as well as visuospatial orientation (Fanselow & Dong, 2010). Of specific interest in BD, disrupted pattern separation and completion processes are hypothesized to underlie the formation of delusional thought content (Tamminga, Stan, & Wagner, 2010). Importantly, the hippocampus consists of anatomically and functionally distinct subfields that may be differentially involved in the pathophysiology of the disorder. Post mortem neuropathological investigations of individuals with BD show lower nonpyramidal somal volume in the *cornu ammonis* (CA) 2/3 region (Konradi et al., 2011) and fewer interneurons in the parasubiculum (Wang et al., 2011) region. Furthermore, individuals with BD show greater neuronal counts in the CA1 and subiculum and higher CA1 oligodendrocyte counts compared to HC (Malchow et al., 2015). Other postmortem studies have reported negative findings (Harrison, Colbourne, & Harrison, 2020), which highlights a lack of consensus and need for more precise interrogation.

MRI studies of hippocampal subfield volumetry in BD have been inconsistent (Haukvik, Tamnes, Soderman, & Agartz, 2018). Among the two largest studies to date (each comprising approximately 200 individuals with BD and 300 HC), one study reported smaller volumes in the CA2/3, CA4/dentate gyrus (DG), presubiculum, and subiculum (Mathew et al., 2014), and the other reported smaller CA2/3, CA4/DG, subiculum, and CA1, but no detectable abnormalities in presubiculum volume (Haukvik et al., 2015) in BD compared to HC, respectively. Furthermore, subfield volume reductions in the left CA4, granular cell layer of the DG, molecular layer, and bilateral tail volumes were reported more pronounced in BD1 than BD2 participants (Cao et al., 2017). Although limited, such evidence suggests that differentiation in subfield hippocampal volumetry may depend on BD subtypes. Hippocampal subfield volumes may also be affected by medication use. Lithium treatment has been associated with less pronounced volume deficiencies in CA2/3, CA4/DG and subiculum (Haukvik et al., 2015; Mathew et al., 2014), and CA1 (Hartberg et al., 2015). Stem cell research has shown that lithium increases progenitor cell proliferation in the DG, lending support to the possible neuroprotective and neurotrophic effects of lithium inferred from other MRI studies (Ferensztajn‐Rochowiak & Rybakowski, 2016). Increased angiogenesis and neurogenesis in the DG have been associated with the use of antidepressant medications (selective serotonin reuptake inhibitors) in individuals with major depressive disorder (Boldrini et al., 2012). Antipsychotic medication use has been linked to elevated hippocampal neurogenesis (olanzapine) and increased cell‐proliferation (clozapine) (Balu & Lucki, 2009), but the results on hippocampal volume from human MRI (Bodnar et al., 2016) and animal (Crum et al., 2016; Schmitt, Weber, Jatzko, Braus, & Henn, 2004) studies are inconclusive.

Given the small size of the hippocampal subfields, it is challenging to obtain valid and reliable hippocampal subfield volume estimates from automated MRI‐based processing tools. Recently, by combining cyto‐ and chemo‐architectural features with macroscopic landmarks, segmentation of the hippocampal subfields from MR‐images has improved, and high‐resolution ex‐vivo data have been used to develop detailed hippocampal subfield atlases (Iglesias et al., 2015), which have proven stable across scanners and time points (Brown et al., 2020). Despite such advances and the refinement of automated segmentation protocols (Iglesias et al., 2015; Pipitone et al., 2014; Yushkevich et al., 2010; Yushkevich et al., 2015) it remains challenging to reproduce findings across studies (Haukvik et al., 2018). This discrepancy could reflect differences in clinical characteristics, but also methodological differences between segmentation algorithms (e.g., discrepancies in subfield delineation which may yield different volume estimates), field strength differences (1.5T, 3T, or higher) and the use of T1 and/or T2 weighted images (Mueller et al., 2018).

The Enhancing Neuro Imaging Genetics through Meta‐Analysis Bipolar Disorder (ENIGMA BD) Working Group has brought BD researchers together from around the world to address the limitations of previous smaller scale MRI studies. More recently, ENIGMA groups have moved beyond cohort level meta‐analyses to pooled, or “mega”‐analyses, where anonymized and unidentifiable individual‐level data are aggregated in a central location, allowing more flexible statistical design (Boedhoe et al., 2018). By applying publicly available, harmonized protocols across retrospective samples, mega‐analyses become feasible, offering benefits over meta‐analyses of studies based on different processing/analysis methodologies (Boedhoe et al., 2018). We previously reported on smaller whole hippocampal volumes in BD compared to HC (Hibar et al., 2016), and in other psychiatric disorders including major depressive disorder (Schmaal et al., 2016), and schizophrenia (van Erp et al., 2016). Moreover, prior studies (Cao et al., 2017; Haukvik et al., 2015; Mathew et al., 2014) of hippocampal subfield volumes in BD used a previous version of the FreeSurfer segmentation algorithm (Van Leemput et al., 2009), and a subsequent meta‐analysis (Haukvik et al., 2018) did not allow for analyses of individual medication effects. Here we extend previous studies by using an individual mega‐analytic approach and a newer more anatomically robust hippocampal subfield FreeSurfer segmentation algorithm to determine whether alterations in specific hippocampal subfields can explain the previously reported lower overall hippocampal volume in BD. By this, we also address the need for replication of neuroimaging studies in clinical samples (Open Science Collaboration, 2015; Thompson et al., 2020). We include secondary analyses of the effects of diagnostic subtype, medication use, and clinical characteristics on hippocampal subfield volumes. In the largest study to date—with pooled data from over 4,600 participants—we hypothesized a robust pattern of lower hippocampal subfield volumes in individuals with BD compared to HC. These deficits were expected to be more severe in BD1 than in BD2. We also expected to find evidence for neuroprotective effects of lithium. Determining the specific pattern of subfield volume reduction in BD may provide further insights into the pathophysiological mechanisms of the disorder.

## 2 METHODS AND MATERIALS

### 2.1 Subject samples

The ENIGMA BD hippocampal subfields project included MRI data and clinical characterization of 4,698 subjects (57% female) from 23 sites worldwide (27 scanners), with *n* = 1,472 individuals with BD (60% female) and *n* = 3,226 HC (56% female). BD diagnoses were assessed according to the Diagnostic and Statistical Manual of Mental Disorders, fourth version (DSM‐IV) or the International Statistical Classification of Diseases and Related Health Problems, 10th Revision (ICD‐10). The sample was split into BD1 (*n* = 1,079, 58% female) or BD2 (*n* = 353, 65% female) for DSM‐IV classified patients. The age range was 18–70 years. Current psychotic symptoms were assessed with the Positive and Negative Symptoms Scale (PANSS). Lifetime psychosis was obtained as a yes/no variable across sites. Clinical and demographic information, as well as estimated current medications are presented in Table 1, whereas site‐specific information is shown in Table S1.

**TABLE 1:** Demographic and clinical information

	Cases (*N* = 1,472)		Controls (*N* = 3,226)			
	*N*	%	*N*	%	χ^2^/Wilcoxon rank‐sum test	*p*‐Value
Females (%)	884	60.1	1,793	55.6	8.1	.0045
	**Mean**	***SD***	**Mean**	***SD***		
Age (years)^a^	37.9	11.9	33.3	11.2	4.6	<.001
AOO (years)^b^	23.2	9.3				
DOI (years)^b^	15.1	10.8				
PANSS positive^c^	9.1	3.1				
PANSS negative^d^	9.4	3.3				
	***N***		***N***			
Lifetime psychosis^e^	403/369/700					
BD1/BD2/BD‐NOS^f^	1,079/353/36					
Medication						
Lithium^e^	363/749/360					
Antipsychotics^e^	549/614/309					
Antiepileptics^e^	363/495/614					
Antidepressants^e^	278/580/614					
Scanner field strength						
1.5T; BD (BD1/BD2/BD‐NOS); HC	436 (337/88/10)		496			
3T; BD (BD1/BD2/BD‐NOS); HC	1,036 (742/265/26)		2,730			

Abbreviations: AAO, age at onset; DOI, duration of illness; BD, bipolar disorder; HC, healthy controls; NOS, not otherwise specified; PANSS, Positive and Negative Syndrome Scale.

^a^ Not normal—applied two‐sided Wilcoxon rank sum test.

^b^ Two hundred and nineteen patients with missing AAO/DOI.

^c^ Nine hundred and sixty‐two patients missing PANSS positive score.

^d^ Nine hundred and sixty‐four patients missing PANSS negative score.

^e^ Yes/no/missing.

^f^ Four patients missing diagnostic category (BD1, BD2, BD.NOS).

All subjects provided written informed consent and all participating sites obtained prior approval from their local ethics committees and institutional review boards, including approval to share anonymized data. The study was conducted in accordance with the Helsinki declaration.

### 2.2 Image processing and analysis

Structural T1‐weighted brain MRI scans were obtained at 23 sites following locally optimized scanner protocols. The majority of scans were acquired on 3T scanners (*n* = 21 scanners/3,766 scans) and the remaining were scanned at 1.5T (*n* = 6 scanners/932 scans), with all diagnostic groups represented across field strengths. Image acquisition parameters for each site are provided in Table S2. Images were processed locally with the automated and validated FreeSurfer software (http://www.freesurfer.net) following standardized ENIGMA protocols for harmonization and quality control across multiple sites (http://enigma.ini.usc.edu; Note S1). Subfield volumes were obtained using the hippocampal subfield segmentation algorithm in FreeSurfer v 6.0.0 (Iglesias et al., 2015) based on information from manual delineations of ultrahigh resolution (~0.1 mm isotropic) ex vivo MRI data. We obtained volumes for 12 subfield regions (CA1, CA2/3, CA4, molecular layer, granule cell layer of the dentate gyrus (GC ML DG; FreeSurfer naming convention), hippocampal tail, subiculum, presubiculum, parasubiculum, fimbria, hippocampal fissure, and the hippocampal amygdala transition area (HATA)), the whole hippocampus, and estimated intracranial volume (ICV). The segmented volumes were assessed for outliers at each site following standardized ENIGMA protocols. Any outlier volumes were visually inspected and removed if the segmentation quality was judged to be inadequate (Note S1).

### 2.3 Statistical analyses

All statistical analyses were performed in R (version 3.5.2; http://R-project.org). Group differences in demographic and clinical variables were assessed with chi‐squared tests for categorical data and *t*‐tests/Wilcoxon rank‐sum tests (Note S2) for normally/non‐normally distributed continuous data, respectively. To assess the normality of distributions, we used the Shapiro–Wilk normality test. We also evaluated the distribution of the participants' whole hippocampus and hippocampus subfield volumes for normality (Figures S1 and S2).

Individual tabular data from all sites were pooled on a secure server at the University of Oslo for centralized analysis. For the main case–control analysis, a linear mixed‐effects (LME) model was used to assess diagnostic differences in the whole hippocampus and hippocampal subfields volumes, with sex, age, age^2^, sex*age, sex*age^2^, and ICV as fixed‐effects variables, and with scanner nested in field strength as random‐effects variables. We included the age^2^ term because the hippocampus shows a nonlinear age‐related trajectory with accelerating atrophy at more advanced age (Fjell et al., 2013). We used the *lme‐function* from the *nlme*‐package to fit the LME models. Because the left and right hemisphere subfield volumes were highly correlated, and we did not have an a priori hypothesis on laterality, the left and right hemisphere volumes of each subfield were combined (summed) in order to reduce the number of tests and increase statistical power. For completeness, we investigated the model for each hemisphere separately. We also performed analyses with field strength added as a fixed factor to address possible confounding effects of field strength on the volume results.

Follow‐up analyses of BD1 versus BD2 subgroup differences were performed using similar LME models that included sex, age, age^2^, sex*age, sex*age^2^, and ICV as fixed‐effects variables, and scanner nested in fields strength as random‐effects variables. Firstly, we compared BD1 and BD2 to each other, and then secondly, we compared each BD subtype to HC in separate analyses, unless otherwise stated. We used this approach to determine the effects of lifetime psychosis (i.e., the occurrence of any episode of psychosis during mood episodes throughout life), which cuts across the BD1/BD2 categorization. In separate analyses, we analyzed associations between current positive or negative psychotic symptoms, duration of illness, age at illness onset and subfield volumes among patients only, while adjusting for BD1/BD2 categorization. Finally, within individuals with BD1, we analyzed the effects of current medication use for each group of medication (i.e., lithium, antipsychotics, antidepressants, or antiepileptics), and performed a joint examination of the effects of all medication groups. Current medication use was stratified into users/non‐users based on the available medication data from each site and for each group of medication. In the first set of medication analyses, we directly compared medication users with non‐users. In the second set of medication analyses, users and non‐users were separately compared to HC. We limited the joint examination of all medication groups to patients only. Medication analyses were restricted to the BD1 group to avoid potential confounding effects of the different medication regimes, clinical characteristics, and hippocampal volumetry associated with BD1 and BD2. We did not perform separate medication analyses in the BD2 group, as it was smaller and had limited information on medication.

We computed Cohen's *d* effect size estimates from the *t*‐statistics from the LME models (Nakagawa & Cuthill, 2007). To adjust for multiple comparisons, Bonferroni correction for *N* tests with *α* = .05 was applied, where *N* is the number of tests for the combined right and left hemisphere subfields (and whole hippocampus) which gives a significance threshold at *p =* .0038 (13 tests). We used forest plots to visualize possible site differences (Note S3).

## 3 RESULTS

### 3.1 Demographic variables

The BD group was significantly older ($\underset{\text{\_}}{\Delta }$ = 4.6/*p* < .001) and included more women (*χ* ^2^ = 10.8/*p* = .0045) than the HC group. Demographic and medication information are listed in Table 1 and shown in Figure S3 (for site‐specific information see Table S1).

Among the individuals with BD1 included in the medication analyses, 165 received antipsychotics, 151 received lithium, 42 received antiepileptics, and 28 received antidepressants. In addition, 168 received lithium in combination with antipsychotics, antiepileptics, antidepressants, or a combination of the three, and 196 received antipsychotics, antiepileptics and/or antidepressants in different combinations (Figure S4 for details). Demographic variables for the BD1 group are listed in Table S3.

### 3.2 Bipolar disorder versus healthy control differences in hippocampus subfield volumes

**FIGURE 1:**
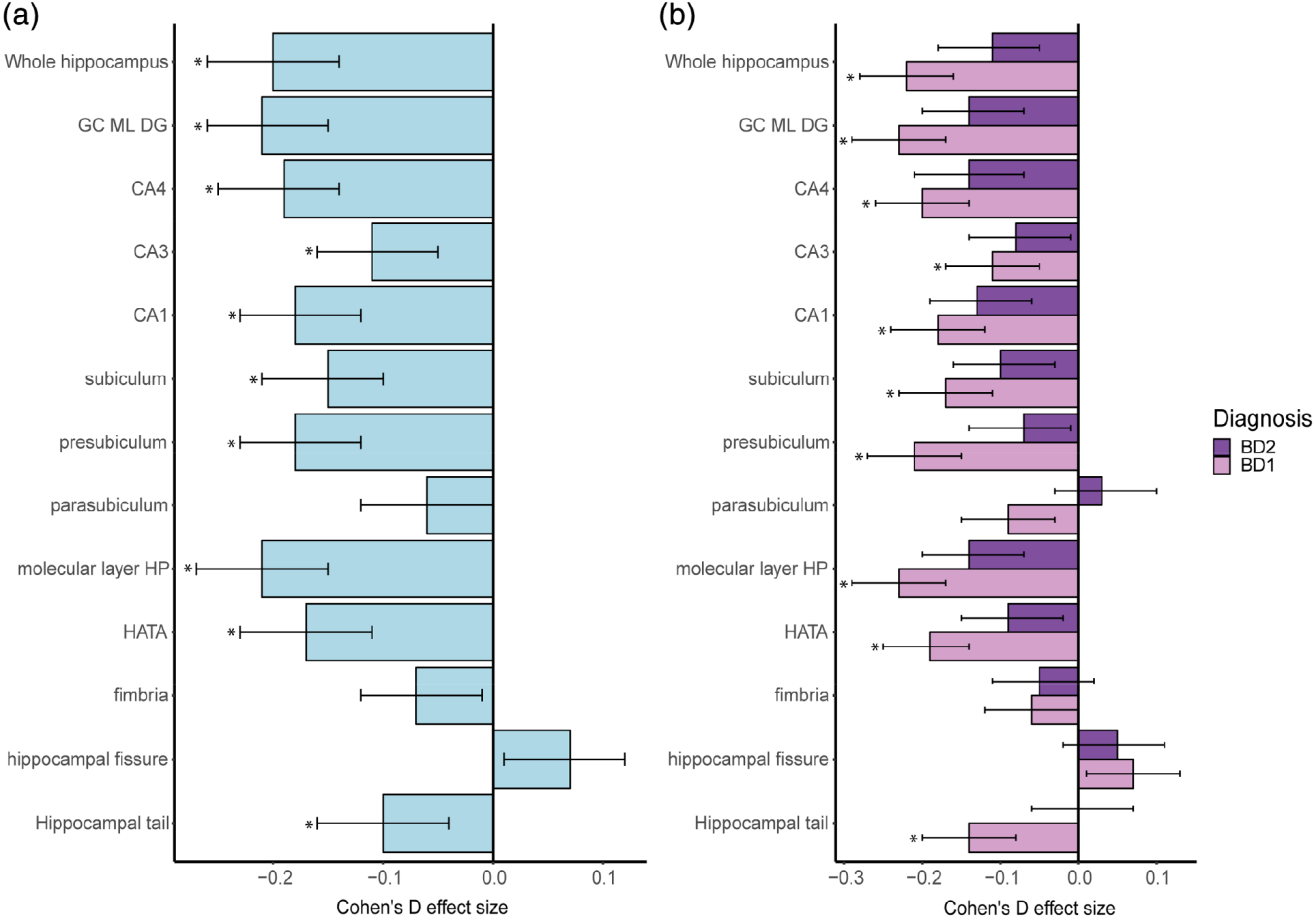
Hippocampal subfield volume differences between patients with bipolar disorder and healthy controls. *Notes*: (a) all bipolar disorder patients compared to healthy controls, (b) patients with bipolar 1 (*n* = 1,079) and bipolar 2 (*n* = 353) compared to healthy controls. Significant differences indicated by *. CA3 implies CA2/3. CA, cornu ammonis; GC ML DG, granule cell layer of dentate gyrus; HATA, hippocampal amygdala transition area; HP, hippocampus

In the main LME analysis, individuals with BD showed significantly smaller whole hippocampus volume (Cohen's *d* = −0.20, *p* = 3.1e−10) compared to HC (Figure 1a, Table S4). Smaller volumes were present across most subfields, including the hippocampal tail, subiculum, presubiculum, CA1, CA2/3, CA4, molecular layer, GC ML DG, and HATA, with the largest effect sizes for the molecular layer (*d* = −0.21) and GC ML DG (*d* = −0.21). Split hemisphere analyses showed a similar pattern of subfield volume reductions in the left and right hippocampus (Figure S5a). Forest plots illustrate the patterns of subfield volume reductions across sites (Figure S6). The introduction of a fixed term for field strength resulted in no significant effect for the latter and did not alter the group analysis results from the main model (data not shown).

### 3.3 Bipolar disorder subtype and clinical characteristics effects on subfield volume

Follow‐up analyses of BD‐subtypes showed no significant volumetric differences between BD1 and BD2 for the whole hippocampus or any of the subfields (Table S5). Smaller whole hippocampus volume was found in BD1 (Cohen's *d* = −0.22, *p* = 8.9e−11) but not BD2 (*d* = −0.11, nominally significant) compared to HC (Figure 1b; Table S6a). In BD1, volumes were smaller across most subfields, including the hippocampal tail, subiculum, presubiculum, CA1, CA2/3, CA4, molecular layer, GC ML DG, and HATA. The effect sizes were slightly larger than in the main analysis (including all BD subtypes), with the largest effects for the GC ML DG (*d* = −0.23) and molecular layer (*d* = −0.23). In BD2, there were nominally significant findings for CA1, CA4, GC ML DG, and molecular layer volumes when compared to HC. Split hemisphere analyses showed a similar pattern of subfield volume alterations for both hemispheres (Figure S5b; Table S6b,c). Forest plots of BD1 and BD2 subfield volumes when compared to HC also showed differences across sites (Figures S7 and S8).

**FIGURE 2:**
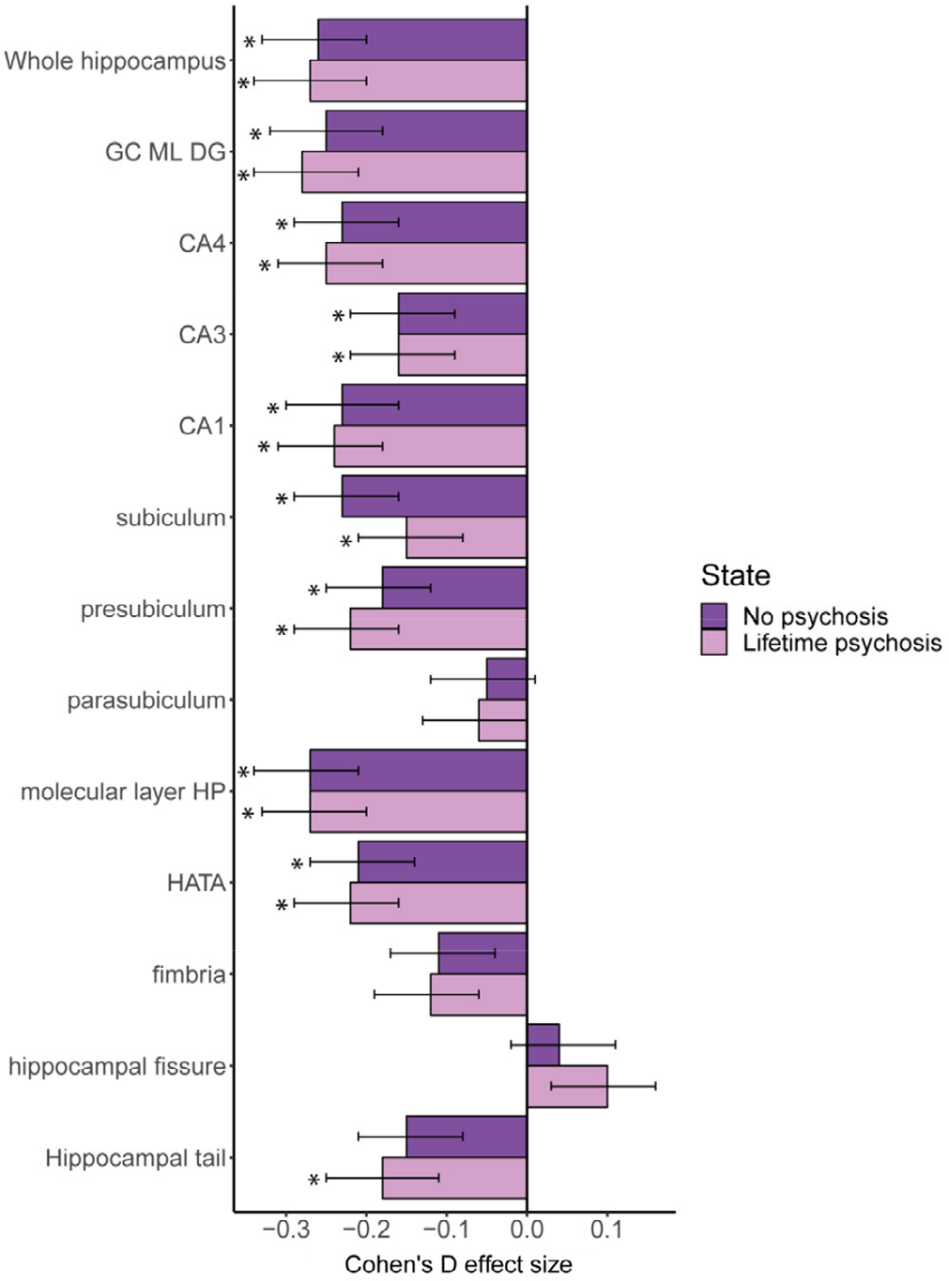
Hippocampal subfield volume differences between bipolar disorder patients with or without a lifetime history of psychosis, and healthy controls. *Notes*: Bipolar patients with (*n* = 403) and without (*n* = 369) a diagnosis of lifetime psychosis compared to controls. Significant differences indicated by *. CA3 implies CA2/3. CA, cornu ammonis; GC ML DG, granule cell layer of dentate gyrus; HATA, hippocampal amygdala transition area; HP, hippocampus

In follow‐up analyses of the whole BD group, patients with and without a history of lifetime psychosis (available in 403 patients versus 369 without) both showed similar effect patterns compared to HC across subfield volumes (Figures 2 and S9; Table S7). Current psychosis symptoms, age at onset, and illness duration were not associated with any of the hippocampal subfield volumes (Tables S8–S11).

### 3.4 Medication effects

**FIGURE 3:**
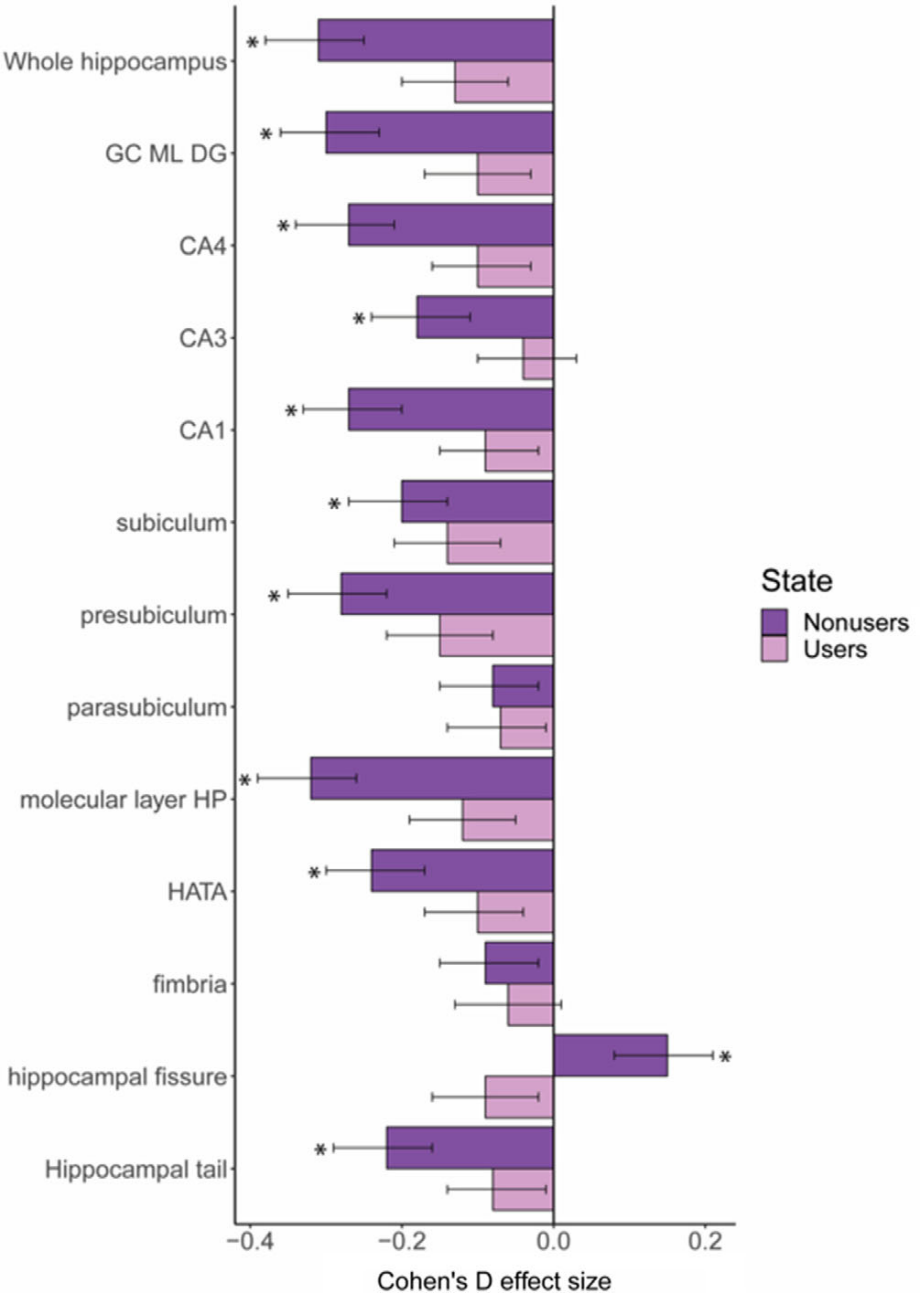
Hippocampal subfield volume differences between lithium users and nonusers among bipolar disorder 1 patients, and healthy controls. *Notes*: Bipolar 1 lithium users (*n* = 319) and nonusers (*n* = 464) compared to healthy controls (reference). Significant structures indicated by *. CA3 implies CA2/3. CA, cornu ammonis; GC ML DG, granule cell layer of dentate gyrus; HATA, hippocampal amygdala transition area; HP, hippocampus

Individuals with BD1 taking lithium at the time of scan (*n* = 319) showed significantly larger volumes for the whole hippocampus (*d* = 0.22, *p* = .0028), molecular layer (*d* = 0.23), GC ML DG (*d* = 0.22), and smaller hippocampal fissure (*d* = −0.24), compared to those not taking lithium (*n* = 464) after applying Bonferroni correction (Table S12). Individuals with BD1 and taking lithium did not show significant volume differences in any subfield or for the whole hippocampus compared to HC. Conversely, individuals with BD1 who were not on lithium showed significant volume reductions compared to HC. Importantly, the effect sizes from the BD1‐nonlithium versus the HC analysis were greater than the effect sizes from the main analysis comparing all BD subjects with HC, with largest effect sizes for the molecular layer (*d* = −0.32), GC ML DG (*d* = −0.30), CA1 (*d* = −0.27), CA4 (*d* = −0.27), presubiculum (*d* = −0.28), and whole hippocampus (*d* = −0.31) (Figure 3; Table S13). These effects were similar bilaterally (Figure S10).

Among individuals with BD1, antipsychotics users (*n* = 472) showed significantly smaller presubiculum volume than antipsychotics non‐users (*n* = 385) (*d* = −0.24, *p* = 7e−04) (Table S14). Significant volume reductions were found across subfields in individuals with BD1 who used antipsychotics relative to HC, with largest effects in presubiculum (*d* = −0.3), molecular layer (*d* = −0.28), GC ML DG (*d* = −0.27), and CA4 (*d* = −0.24) (Figure S11a; Table S15) and these effects were bilateral (Figure S12a). Individuals with BD1 who did not use antipsychotics had smaller volumes of the GC ML DG (*d* = −0.16) and CA4 (*d* = −0.15) compared to HC. The effect sizes for the volume reductions were larger in individuals with BD1 who used antipsychotics than in non‐users, when compared to HC.

Individuals with BD1 who used antiepileptics (*n* = 256) had significantly smaller hippocampal tail (*d* = −0.25) volumes compared to individuals with BD1 who did not use antiepileptics (*n* = 309) (Table S16). Compared to HC, both individuals with BD1 who used and who did not use antiepileptics showed volume alterations similar to those detected with the main model (All BD versus HC)—with larger effect sizes among individuals with BD1 who used antiepileptics (Figures S11b and S12b; Table S17).

Antidepressant use was not associated with specific subfield volume differences among individuals with BD1 (Table S18), and both antidepressant users (*n* = 155) and nonusers (*n* = 410) showed a similar pattern of smaller subfield volumes compared to HC (Figures S11c and S12c; Table S19).

Finally, to better understand the potential medication effects in individuals on more than one medication at the time of scan, a model in which all medications were included as fixed covariates was used to determine the potential effect of each medication while controlling for all other medications. We had complete medication information for a subset of individuals with BD1 (*n* = 565), of which *n* = 53 were unmedicated at the time of scanning. Of the medicated patients (*n* = 512), there were *n* = 181 lithium users, *n* = 343 antipsychotics users, *n* = 155 antiepileptics users, and *n* = 256 antidepressant users in different combinations (see Figure S13 for details). Antipsychotics users showed significantly smaller presubiculum volume (*d* = −0.28) compared to non‐users when adjusting for lithium, antiepileptics, and antidepressant use. No other medication showed a significant volume effect when simultaneously adjusting for all other medications (Table S20).

## 4 DISCUSSION

This largest study of hippocampal subfield volumes in BD to date, had five key findings: (a) individuals with BD had smaller volumes across most subfields compared to HC, (b) individuals with the BD1 subtype showed largest effect sizes when compared to HC, (c) volumes in lithium users did not differ from HC, (d) antipsychotics and antiepileptics users showed smaller volumes compared to non‐users, and (e) altered volumes were not associated with other clinical characteristics. By pooling data sets to include over 4,600 participants, these results confirm and extend our current knowledge of hippocampal structure in BD.

The main finding of smaller subfield volumes across the hippocampal subfields in BD is partly in line with the results from prior studies (Cao et al., 2017; Haukvik et al., 2015; Mathew et al., 2014) and one meta‐analysis (Haukvik et al., 2018). In accordance with previous heterogeneous results, the forest plots demonstrate a high degree of heterogeneity across the included sites, which may help explain the disparate findings across prior studies. We found largest effect sizes for volume differences between BD and HC for the molecular layer, presubiculum, GC ML DG, CA4, and CA1, in line with previous findings (Haukvik et al., 2018). The molecular layer was not delineated as an independent structure in the earlier version of the FreeSurfer hippocampal subfield segmentation algorithm (Van Leemput et al., 2009), which was used in most prior studies. However, Cao et al. found smaller molecular layer volumes in BD with the same subfield segmentation as used in the present study (Cao et al., 2017). The molecular layer stretches as a dark band from the DG along the CA subfields to the subiculum. It is relatively cell free (Iglesias et al., 2015) but contains dendrites from DG neurons (Amaral, Scharfman, & Lavenex, 2007). We may speculate that the smaller molecular layer could reflect loss of dendritic connections or DG neurons of which hyperexcitability has been linked to successful lithium treatment in BD (Mertens et al., 2015; Stern et al., 2018). Some (Mathew et al., 2014), but not all (Cao et al., 2017; Haukvik et al., 2015), prior studies have reported smaller presubiculum volumes in BD. The presubiculum is part of the outflow region of the hippocampus—together with the parasubiculum and the more well‐defined subiculum subfields—and is involved in visuospatial processing and orientation (Dalton & Maguire, 2017; Simonnet et al., 2017). As expected from prior findings, individuals with BD also showed lower volumes in the CA4, CA1, GC ML DG, and in the subiculum (Haukvik et al., 2018). Finally, we found significantly lower CA2/3 volume in BD, as hypothesized on the basis of previous studies (Cao et al., 2017; Haukvik et al., 2015; Mathew et al., 2014) and the presumed role of this subfield in the pattern completion thought to underlie the formation of psychotic thought content (Tamminga et al., 2010).

We found that the effect sizes for the case–control volume differences across the hippocampus were larger in BD1 than BD2. This is in line with the results from Cao et al. (2017), and may suggest a stronger hippocampus related neurobiological component in BD1 than in BD2. However, given the lack of significant differences in the direct BD1 versus BD2 comparison, it could also reflect the smaller number of individuals with BD2. The BD2 results may also be confounded by the greater clinical heterogeneity that is displayed by the BD2 compared to the BD1 subtype (Phillips & Kupfer, 2013).

Medication use, in particular the use of lithium, was associated with the subfield volumes across the hippocampus, which confirms and extends results from prior studies (Bearden et al., 2008; Hartberg et al., 2015; Simonetti et al., 2016). Lithium users did not show significant volume deficiencies compared to healthy controls, whereas the nonusers did. These results may reflect a neuroprotective effect of lithium on hippocampal subfield volumes, which is in line with previous whole brain MRI volumetric studies (Berk et al., 2017). In accordance with this understanding are also animal studies showing enhanced neural proliferation (Zanni et al., 2017), stem cell studies showing increased progenitor cell proliferation (Ferensztajn‐Rochowiak & Rybakowski, 2016), the increased numbers of neurons and glia in the DG (Rajkowska et al., 2016) following lithium use shown in postmortem studies. Furthermore, we observed that antipsychotic or antiepileptic medication use was associated with smaller subfield volumes, and the effects of antipsychotic use on the presubiculum volume remained significant after controlling for all other psychopharmacological use. The smaller volumes are consistent with previous BD studies showing a negative association between antipsychotics or antiepileptics and neurostructural measures (Fusar‐Poli et al., 2013; Hibar et al., 2016). Antipsychotics have been shown to increase hippocampal neurogenesis and cell proliferation (Balu & Lucki, 2009), but the effects on hippocampal volumes from human MRI (Bodnar et al., 2016) and animal (Crum et al., 2016; Schmitt et al., 2004) studies have been mixed. Our results, taken together with the lack of an association between duration of illness, current or lifetime psychosis, or age at illness onset on any of the subfield volumes, may suggest that putative neurogenic and cell‐proliferative effects of antipsychotic medication were not large enough to affect hippocampal subfield volumes. We did not observe any association between subfield volumes and anti‐depressant medication, despite previous reports of subfield‐volume enlargement in patients with major depressive disorder after selective serotonin reuptake inhibitor or serotonin nor‐adrenalin reuptake inhibitor treatment (Katsuki et al., 2020; Maller et al., 2018). This could be due to confounding by other medication use or symptom pathophysiology or severity characteristics within our sample.

Certain limitations of our study should be noted. We were not able to control for possible confounding factors such as alcohol or substance abuse, IQ, number of depressive or manic episodes, or current mood state, as these variables were only available for some subsets of the participants and were not directly comparable as they were obtained with different cognitive‐ and psychometric tests. We could not control for socioeconomic status or childhood trauma, or other comorbid brain disorders, which may also influence hippocampal volume (Aas et al., 2014; Teicher, Anderson, & Polcari, 2012). Medication effects were studied in current users versus current non‐users since information on treatment duration or dosages was not available. Information on current medication status was only available in a subsample, which reduced the power to detect differences among BD1 patients when stratified on medication group. The multisite MRI acquisition invariably includes the use of different scanners and scanning parameters, as well as different field strengths (1.5T and 3T). We only had available T1‐weighted MRI data, and a combination of T1‐ and T2‐weighted MRI data has been reported to increase segmentation accuracy (Iglesias et al., 2015). All participating sites included both BD patients and HC (except for the Medellín, one of the Deakin sites, and both Milan sites, which lacked HC). We controlled for scanners and magnetic field strengths by including them as random‐effects in the model, which may not fully account for differences across sites. While standardized processing pipelines have been shown to reduce cross‐site variability, true cross‐site harmonization is only possible through coordinated prospective data collection.

Major strengths of this study include the large sample size, the use of ENIGMA‐standardized processing pipelines to derive hippocampal subfield volumes across sites, and the mega‐analysis of pooled data. This study design helps to overcome some of the key limitations of classic, literature‐based meta‐analyses (e.g., the combination of effect sizes from studies that may differ widely with respect to processing and analysis methodology) and previous smaller‐scale studies with limited statistical power (Paulus & Thompson, 2019; Westlye, Alnaes, van der Meer, Kaufmann, & Andreassen, 2019). By pooling standardized brain measures across a diverse set of BD neuroimaging studies, we have created a more ecologically valid cohort, which may provide a more replicable picture of hippocampal subfield alterations in BD as the illness presents around the world.

In conclusion, lower overall hippocampal volumes in BD were traced to smaller volumes across the majority of the hippocampal subfields. The effects were largest in the BD1 group, not specific to current or lifetime psychosis, and influenced by medication use. The lack of detectable group differences between lithium users and HC supports the notion of a possible neuroprotective role of lithium in BD. These results demonstrate the power of large‐scale multisite efforts to disentangle clinical and methodological heterogeneity and address the need for replication studies. Given the overlapping findings of lower whole hippocampal volumes in the largest neuroimaging ENIGMA studies of BD (Hibar et al., 2016), major depression (Schmaal et al., 2016), and schizophrenia (van Erp et al., 2016), further studies of hippocampal subfields may allow for neurobiological differentiation across major mental illnesses.

## DISCLOSURE OF INTERESTS

UKH, TPG, SN, TNA, TH, MA, DA, DMC, SA, JB, BTB, FB, FB, CMB, PB, EJC, XC, OD, UD, GD, AMD, MF, SFF, KF, JMF, JMG, DG, OG, BCH, BH, BH, MAH, ELH, FMH, CH, KNJ, TK, BK, AK, RK, TVL, TML, RKL, VL, CLJ, CM, EMTM, GM, IM, PBM, LN, IN, VO, LO, NO, MCO, BJO, JAP, EP, JR, LR, RR, JR, MMR, GR, HGR, LES, RS, SS, JS, AHS, KS, MGS, MS, FS, CKT, HST, SIT, DvdM, DJV, TGMvE, EV, LW, LTW, HCW, PGS, and IA declare no conflicts of interest.

MB: was supported by an unrestricted grant from AstraZeneca; TE, UFM, EB: has received speaker's fees from Lundbeck AS, and Janssen Cilag; NJ, PMT: MPI of a research grant from Biogen, Inc. for work unrelated to the contents of this manuscript; CRKC: is partially funded by a Biogen Grant (to NJ and PMT) for research unrelated to the contents of this manuscript. DJS: has received research grants and/or honoraria from Lundbeck and Sun; OAA: has received speaker's honorarium from Lundbeck, and is a consultant to HealthLytix. AMM: has received research support from Eli Lilly, Janssen, and the Sackler Foundation, and has also received speaker fees from Illumina and Janssen. All other authors report no biomedical financial interests or potential conflicts of interest.

## AUTHOR CONTRIBUTIONS

*Cohort PI*: BTB, FB, DMC, XC, UD, TE, OG, BCMH, TH, FMH, TK, AK, CLJ, AM, IM, PBM, IN, EP, RS, JS, KS, MGS, EV, HCW, AMM, OAA, IA. *Project development*: UKH, TPG, SN, MA, SA, BTB, UD, MF, JMF, NJ, TK, RKL, VL, EP, RS, SS, HST, SIT, PMT, CRKC, IA. *Imaging methods*: PGS, UKH, TPG, SN, NJ. *Illustrations*: TPG. *Data interpretation*: UKH, TPG, FB, UD, KF, DG, TH, CH, BK, NO, RR, LW. *Manuscript preparation*: UKH, TPG, RR, CRKC, OAA, IA. *Imaging Data collection*: UKH, SN, TNA, TH, MA, SA, JB, MB, CB, PB, XC, UD, GD, AMD, TE, MF, SFF, KF, JMG, DG, OG, BCH, TH, BH, MAH, ELH, CH, FMH, KNJ, TK, RK, RKL, TVL, CM, GM, IM, EMTM, PBM, LN, VO, LO, NO, MCO, BJO, EP, RR, CMB, JR, MMR, GR, HGR, SS, JS, AHS, KS, MGS, MS, FS, HST, DJV, LW, OAA, IA, UFM, EB, AMM. *Manuscript revision*: UKH, MB, TPG, TE, MA, DA, SA, JB, FB, EJC, XC, UD, MF, KF, JMF, DG, OG, OD, BCMH, BCH, BTB, TH, MAH, CH, FMH, KNJ, BK, TVL, TML, RKL, IM, PBM, NO, EP, JR, GR, RS, HGR, SS, JS, KS, MGS, DJS, CKT, HST, DvdM, TGMvE, EV, LW, LTW, NJ, LES, PMT, CRKC, OAA, AMM, EB.

## Supporting information


**Data S1**: Supporting information.

## Data Availability

The datasets from this study will not be made publicly available as we do not have approvals for sharing clinical data.
