# Supplementary material for: In vivo hippocampal subfield volumes in bipolar disorder—A mega‐analysis from The Enhancing Neuro Imaging Genetics through Meta‐Analysis Bipolar Disorder Working Group
Source: Hum Brain Mapp. 2020 Oct 19;43(1):385–98. doi: 10.1002/hbm.25249 (PMC8675404; doi:10.1002/hbm.25249)
Supplement: Supplementary file 1 — Data S1: Supporting information. [file HBM-43-385-s001.pdf]

Supplemental Information

Table of contents

SUPPLEMENTAL FIGURES 5

Figure S1: Violin plots of the participants combined, left and right hippocampal volumes split on patients and healthy controls. 5

Figure S2-a: Violin plots of the participants combined hippocampal subfield volumes split on patients and controls. 6

Figure S2-b: Violin plots of the participants left hippocampal subfield volumes split on patients and controls. 7

Figure S2-c: Violin plots of the participants right hippocampal subfield volumes split on patients and controls. 8

Figure S3: Density plots of demographic variables – all patients and controls. 9

Figure S4: Medication use combinations among Bipolar 1 patients. 10

Figure S5: Left and right hippocampal subfield volume differences between patients with bipolar disorder and healthy controls (reference). 11

Figure S6-a: Forest plots with site-specific hippocampal subfield volume differences between bipolar disorder patients and healthy controls. 12

Figure S6-b: Forest plots with site-specific hippocampal subfield volume differences between bipolar disorder patients and healthy controls. 13

Figure S6-c: Forest plots with site-specific hippocampal subfield volume differences between bipolar disorder patients and healthy controls. 14

Figure S7-a: Forest plots with site-specific hippocampal subfield volume differences between bipolar disorder 1 patients and healthy controls. 15

Figure S7-b: Forest plots with site-specific hippocampal subfield volume differences between bipolar disorder 1 patients and healthy controls. 16

Figure S7-c: Forest plots with site-specific hippocampal subfield volume differences between bipolar disorder 1 patients and healthy controls. 17

Figure S8-a: Forest plots with site-specific hippocampal subfield volume differences between bipolar disorder 2 patients and healthy controls. 18

Figure S8-b: Forest plots with site-specific hippocampal subfield volume differences between bipolar disorder 2 patients and healthy controls. 19

Figure S8-c: Forest plots with site-specific hippocampal subfield volume differences between bipolar disorder 2 patients and healthy controls. 20

Figure S9: Left and right hippocampal subfield volume differences between bipolar disorder patients with and without a diagnosis of lifetime psychosis, and healthy controls (reference). 21

Figure S10: Left and right hippocampal subfield volume differences between lithium users and non-users among bipolar disorder 1 patients, and healthy controls (reference). 22

Figure S11: Effects of antipsychotic, antiepileptic and antidepressant medication on the hippocampal subfield volumes in bipolar disorder 1 (with controls as reference). 23

Figure S12-a: Left and right hippocampal subfield volume differences between antipsychotic users and non-users among bipolar disorder 1 patients, and healthy controls (reference). 24

Figure S12-b: Left and right hippocampal subfield volume differences between antiepileptic users and non-users among bipolar disorder 1 patients, and healthy controls (reference). 25

Figure S12-c: Left and right hippocampal subfield volume differences between antidepressant users and non-users among bipolar disorder 1 patients, and healthy controls (reference). 26

Figure S13: Medication use combinations among Bipolar 1 patients with known medication status for all four medication groups. 27

SUPPLEMENTAL TABLES 28

Table S1: Demographics for the 23 sites/27 scanners of the study. 28

Table S2: Image acquisition parameters for the 23 sites/27 scanners of the study. 30

Table S3: Demographic and clinical information for the bipolar 1 subgroup and controls. 34

Table S4: Hippocampal subfield volumes in bipolar disorder patients compared to controls (reference). 35

Table S5: Hippocampal subfield volumes in bipolar disorder 1 compared to bipolar disorder 2 patients (reference). 36

Table S6-a: Hippocampal subfield volumes in bipolar disorder 1 and bipolar disorder 2 patients compared to controls (reference) (both hemispheres combined). 37

Table S6-b: Hippocampal subfield volumes in bipolar disorder 1 and bipolar disorder 2 patients compared to controls (reference) (left hemisphere). 38

Table S6-c: Hippocampal subfield volumes in bipolar disorder 1 and bipolar disorder 2 patients compared to controls (reference) (right hemisphere). 39

Table S7-a: The effect of lifetime psychosis on hippocampal subfield volumes compared with controls (reference) (both hemispheres combined). 40

Table S7-b: The effect of lifetime psychosis on hippocampal subfield volumes compared with controls (reference) (left hemisphere). 41

Table S7-c: The effect of lifetime psychosis on hippocampal subfield volumes compared with controls (reference) (right hemisphere). 42

Table S8: Effect of age at illness onset on hippocampal subfields volumes across bipolar disorder 1 and bipolar disorder 2 patients. 43

Table S9: Effect of duration of illness on hippocampal subfields volumes across bipolar disorder 1 and bipolar disorder 2 patients. 44

Table S10: Effect of total PANSS positive scores on hippocampal subfield volumes across bipolar disorder 1 and bipolar disorder 2 patients. 45

Table S11: Effect of total PANSS negative score on hippocampal subfield volumes across bipolar disorder 1 and bipolar disorder 2 patients. 46

Table S12: Effect of lithium medication on hippocampal subfield volumes among bipolar disorder 1 patients (reference lithium non-users). 47

Table S13-a: Effect of lithium medication on hippocampal subfield volumes among bipolar disorder 1 patients (lithium users and non-users) with controls as reference (both hemispheres combined). 48

Table S13-b: Effect of lithium medication on hippocampal subfield volumes among bipolar disorder 1 patients (lithium users and non-users) with controls as reference (left hemisphere). 49

Table S13-c: Effect of lithium medication on hippocampal subfield volumes among bipolar disorder 1 patients (lithium users and non-users) with controls as reference (right hemisphere). 50

Table S14: Effects of antipsychotic medication on hippocampal subfield volumes among bipolar disorder 1 patients (reference antipsychotic non-users). 51

Table S15-a: Effect of antipsychotic medication on hippocampal subfield volumes among bipolar disorder 1 patients (antipsychotic users and non-users) with controls as reference (both hemispheres combined). 52

Table S15-b: Effect of antipsychotic medication on hippocampal subfield volumes among bipolar disorder 1 patients (antipsychotic users and non-users) with controls as reference (left hemisphere). 53

Table S15-c: Effect of antipsychotic medication on hippocampal subfield volumes among bipolar disorder 1 patients (antipsychotic users and non-users) with controls as reference (right hemisphere). 54

Table S16: Effect of antiepileptic medication on hippocampal subfield volumes among bipolar disorder 1 patients (reference antiepileptic non-users). 55

Table S17-a: Effect of antiepileptic medication on hippocampal subfield volumes among bipolar disorder 1 patients (antiepileptic users and non-users) with controls as reference (both hemispheres combined). 56

Table S17-b: Effect of antiepileptic medication on hippocampal subfield volumes among bipolar disorder 1 patients (antiepileptic users and non-users) with controls as reference (left hemisphere). 57

Table S17-c: Effect of antiepileptic medication on hippocampal subfield volumes among bipolar disorder 1 patients (antiepileptic users and non-users) with controls as reference (right hemisphere). 58

Table S18: Effect of antidepressant medication on hippocampal subfield volumes among bipolar disorder 1 patients (reference antidepressant non-users). 59

Table S19-a: Effect of antidepressant medication among bipolar disorder 1 patients (antidepressant users and non-users) with controls as reference (both hemispheres combined). 60

Table S19-b: Effect of antidepressant medication on hippocampal subfield volumes among bipolar disorder 1 patients (antidepressant users and non-users) with controls as reference (left hemisphere). 61

Table S19-c: Effect of antidepressant medication on hippocampal subfield volumes among bipolar disorder 1 patients (antidepressant users and non-users) with controls as reference (right hemisphere). 62

Table S20-a: Effect of lithium, antipsychotic, antidepressant, and antiepileptic medication among bipolar disorder 1 patients (reference non-user of the respective medication; both hemispheres combined). 63

Table S20-b: Effect of lithium, antipsychotic, antidepressant, and antiepileptic medication among bipolar disorder 1 patients (reference non-user of the respective medication; left hemisphere). 64

Table S20-c: Effect of lithium, antipsychotic, antidepressant, and antiepileptic medication among bipolar disorder 1 patients (reference non-user of the respective medication; right hemisphere). 65

SUPPLEMENTAL NOTES 66

Note S1: Quality control procedure 66

Note S2: Statistical analyses 66

Note S3: Forest plots 67

REFERENCES 67

Supplemental Figures

Figure S1: Violin plots of the participants combined, left and right hippocampal volumes split on patients and healthy controls.

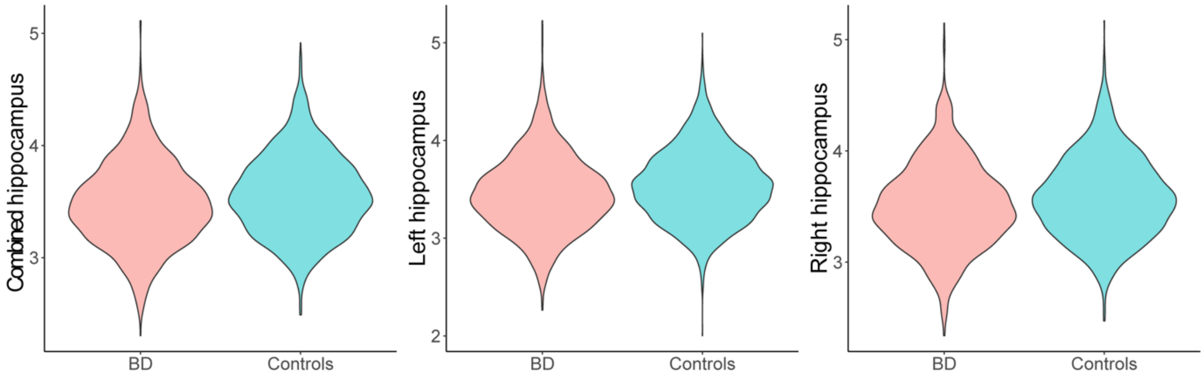

Notes: Combined, left and right whole hippocampus volumes. Distributions are presented in ml. *Abbreviations:* BD - Bipolar disorder.

Figure S2-a: Violin plots of the participants combined hippocampal subfield volumes split on patients and controls.

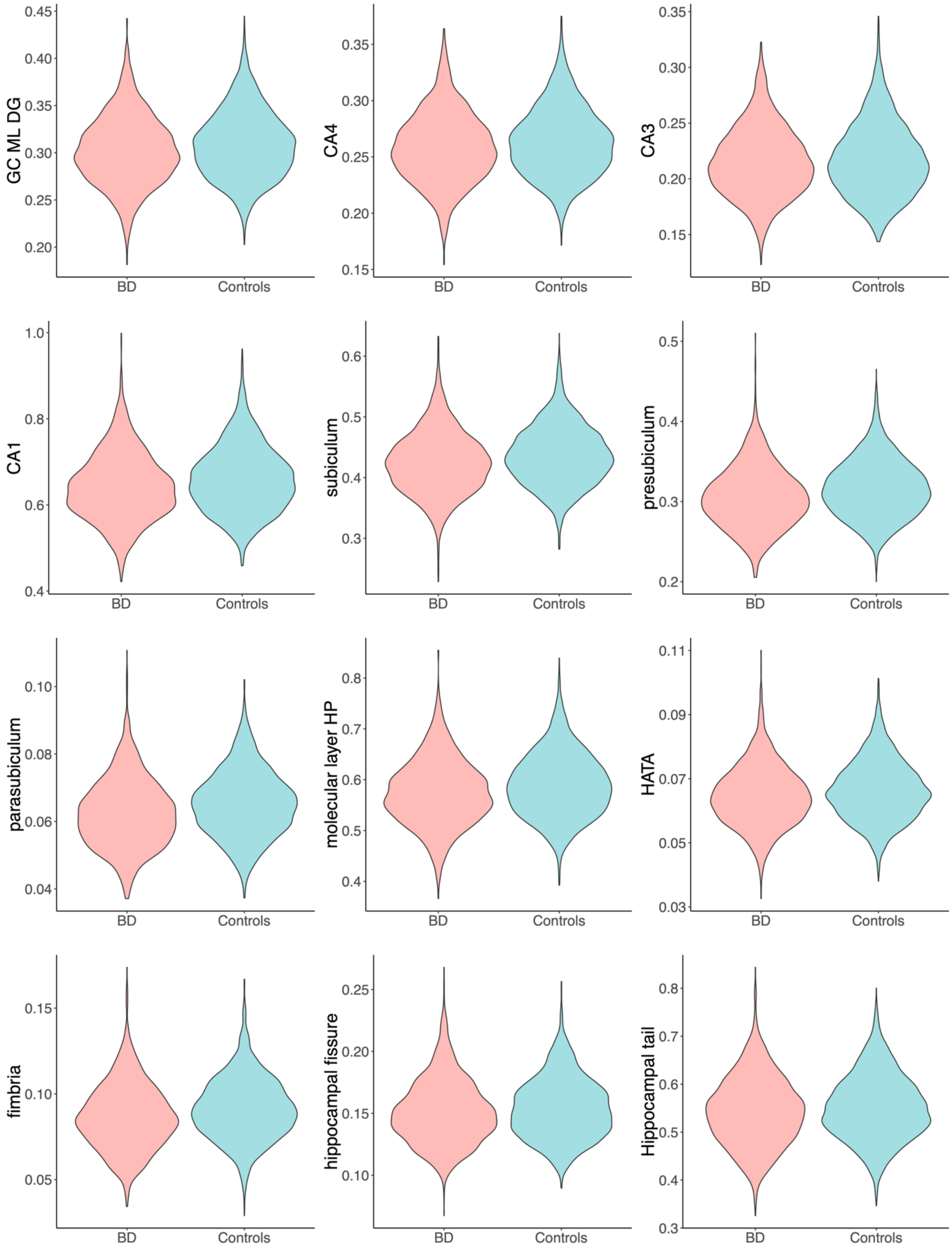

Notes: Combined (left + right) subfields volumes. Distributions are presented in ml. *Abbreviations:* BD - Bipolar disorder.

Figure S2-b: Violin plots of the participants left hippocampal subfield volumes split on patients and controls.

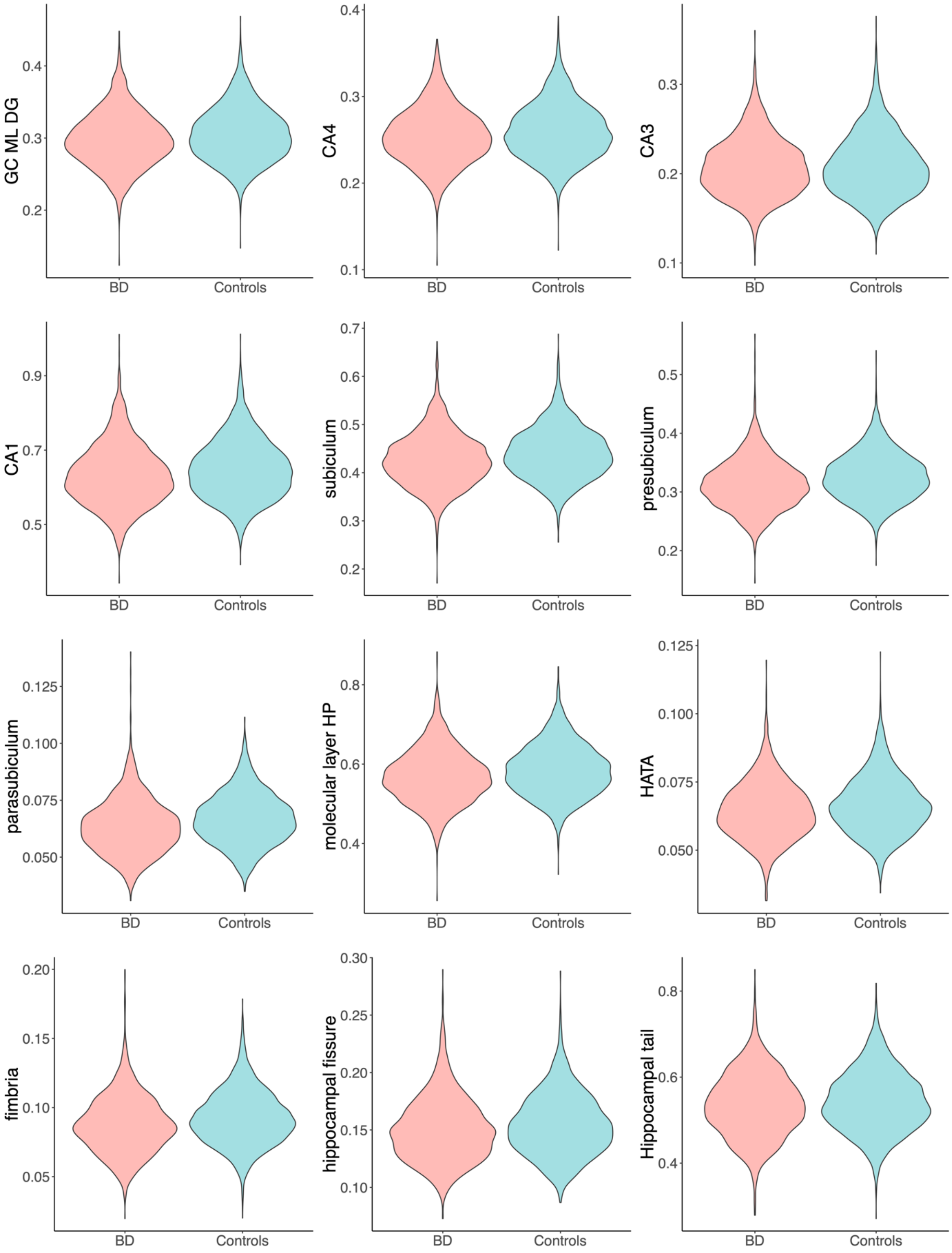

Notes: Left subfield volumes. Distributions are presented in ml. *Abbreviations:* BD - Bipolar disorder.

Figure S2-c: Violin plots of the participants right hippocampal subfield volumes split on patients and controls.

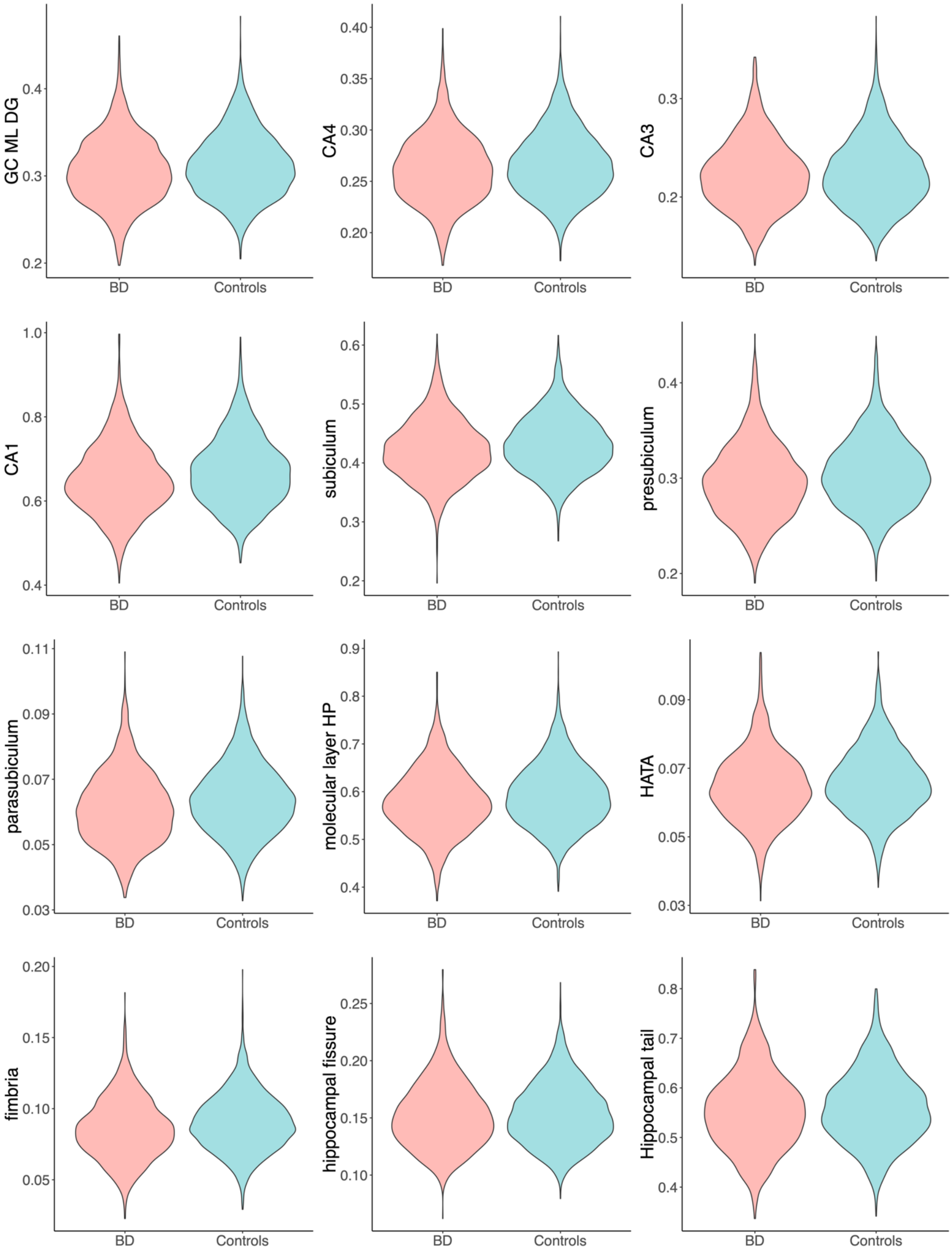

Notes: Right subfield volumes. Distributions are presented in ml. *Abbreviations:* BD - Bipolar disorder.

Figure S3: Density plots of demographic variables – all patients and controls.

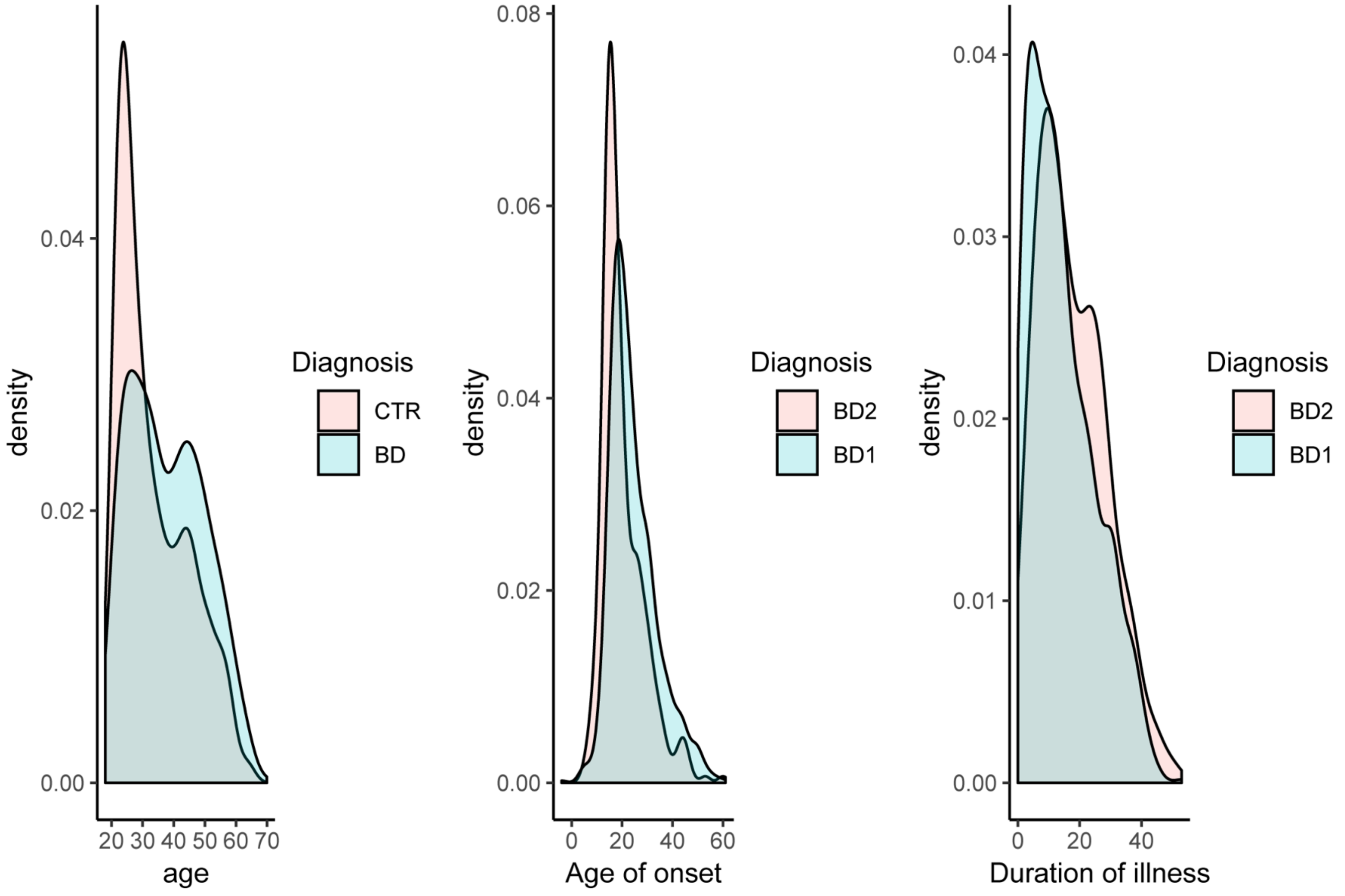

Abbreviations: BD – bipolar disorder; CTR – controls.

Figure S4: Medication use combinations among Bipolar 1 patients.

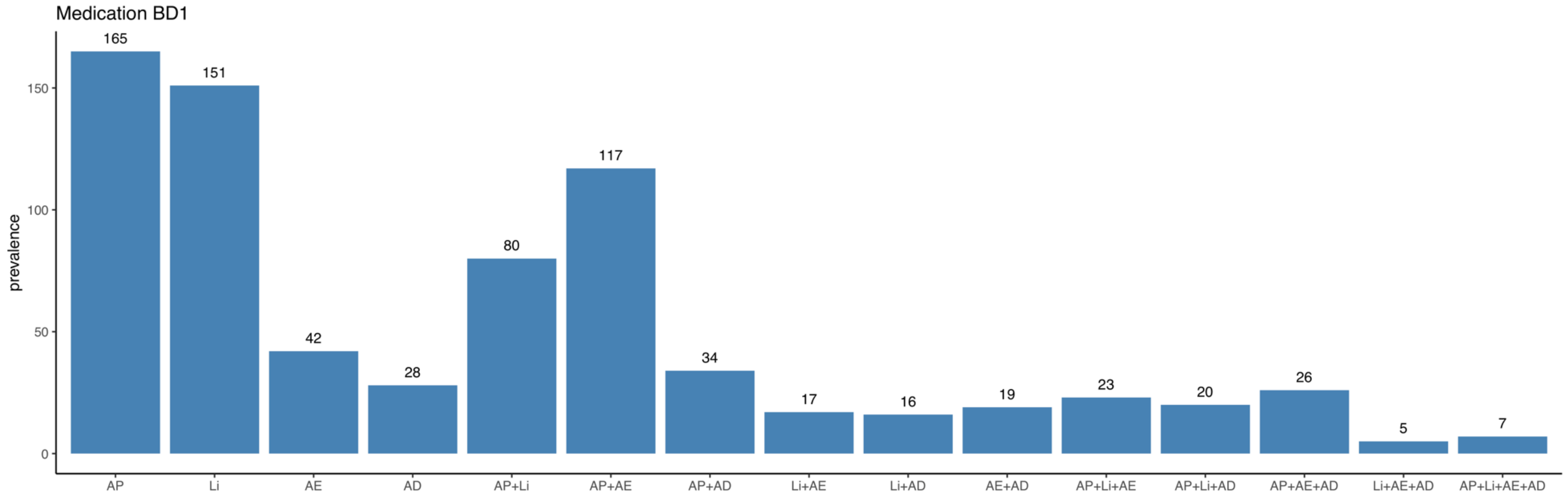

Abbreviations: AP: Antipsychotics, Li: Lithium, AE: Antiepileptics, AD: Antidepressants.

Figure S5: Left and right hippocampal subfield volume differences between patients with bipolar disorder and healthy controls (reference).

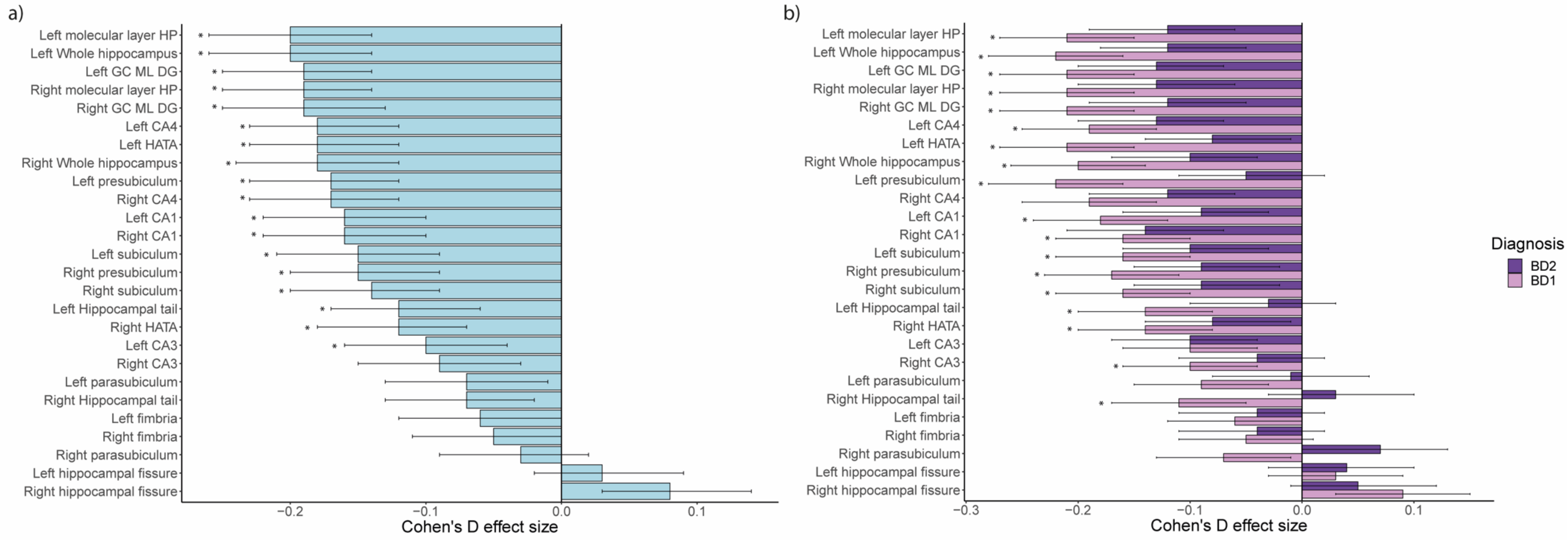

Notes: a) all bipolar disorder patients compared to healthy controls, b) patients with bipolar disorder 1 (n=1079) and bipolar 2 (n=353) compared to healthy controls. Effect sizes are ordered based on ranked effect sizes (rounded to two decimal points) from a), i.e. all patients compared to controls. Significant differences indicated by *. CA3 implies CA2/3. *Abbreviations:* CA – cornu ammonis; GC ML DG - granule cell layer of dentate gyrus; HATA - hippocampal amygdala transition area; HP - hippocampus.

Figure S6-a: Forest plots with site-specific hippocampal subfield volume differences between bipolar disorder patients and healthy controls.

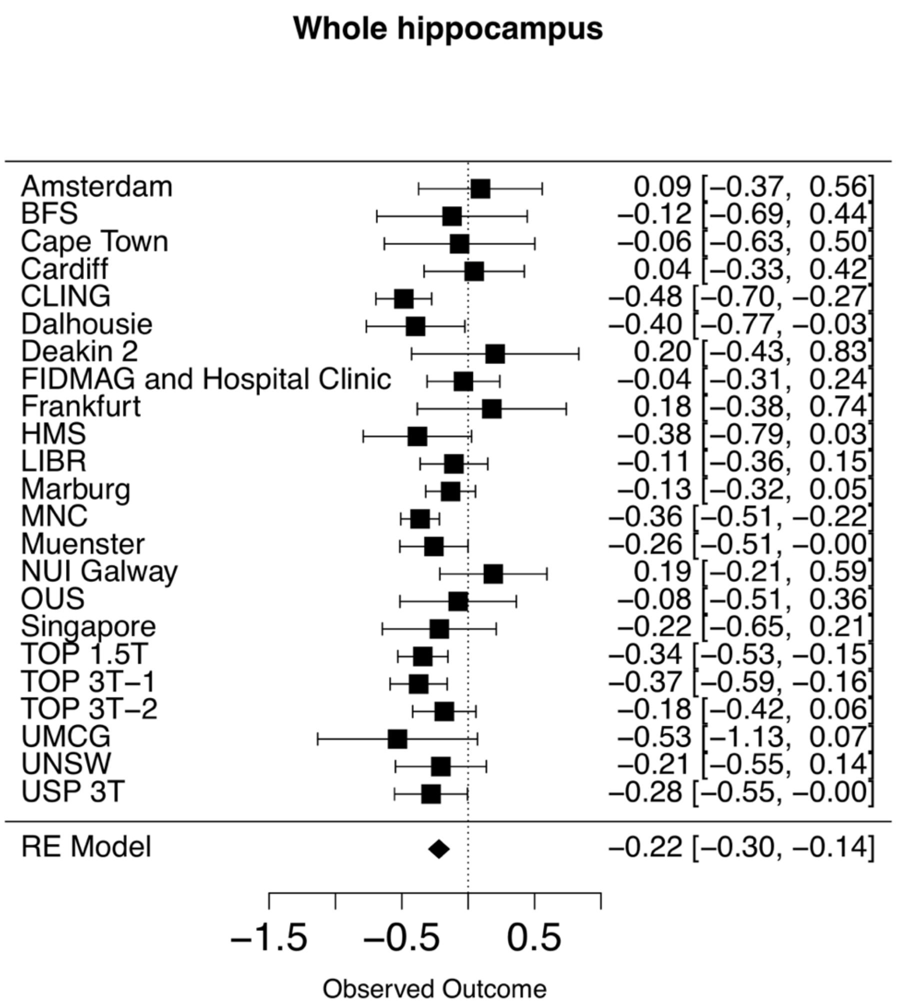

Notes: Sites/scanners that did not acquire healthy control data are not represented in these figures.

Figure S6-b: Forest plots with site-specific hippocampal subfield volume differences between bipolar disorder patients and healthy controls.

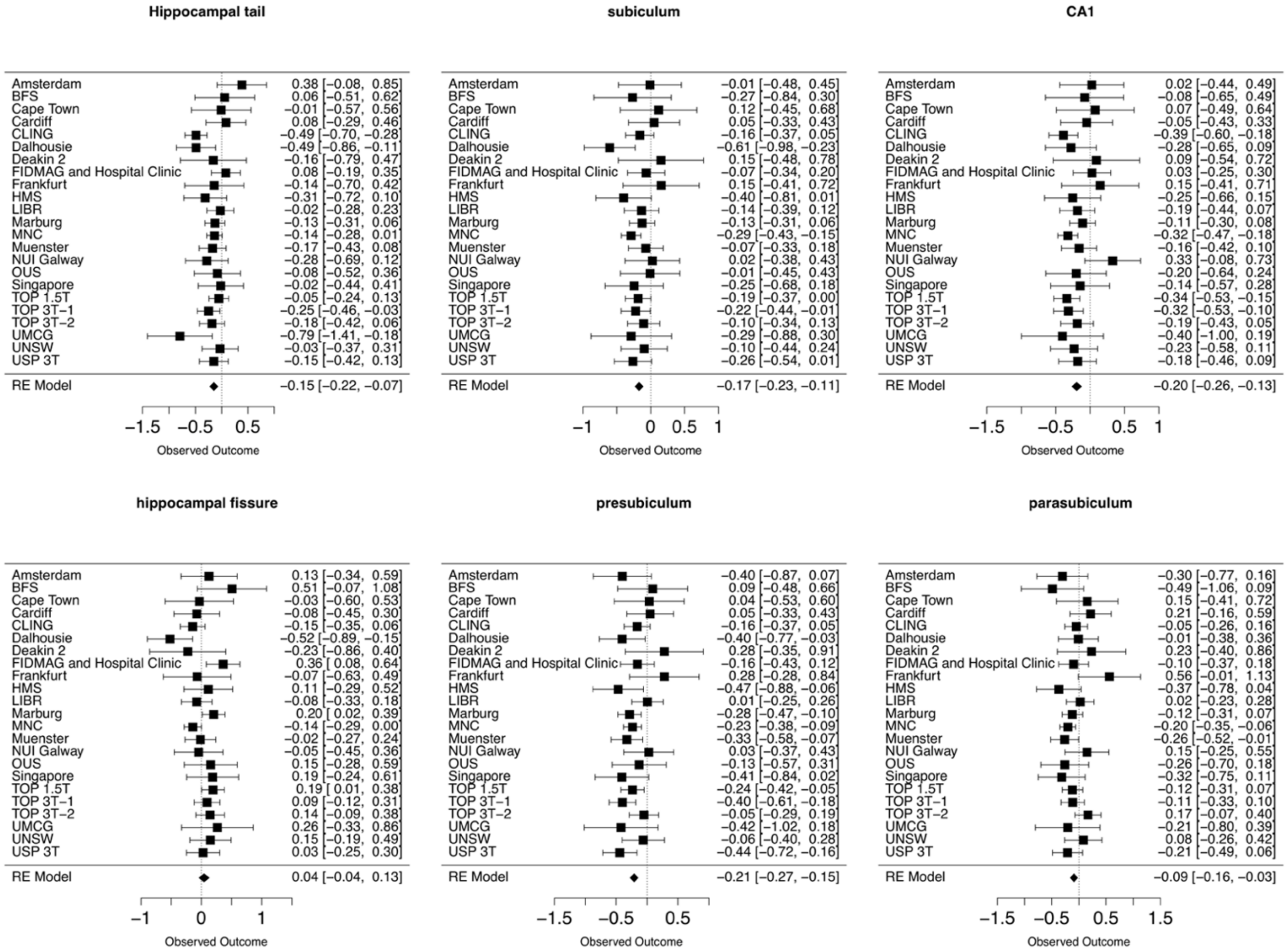

Notes: Sites/scanners that did not acquire healthy control data are not represented in these figures. *Abbreviations:* CA – cornu ammonis.

Figure S6-c: Forest plots with site-specific hippocampal subfield volume differences between bipolar disorder patients and healthy controls.

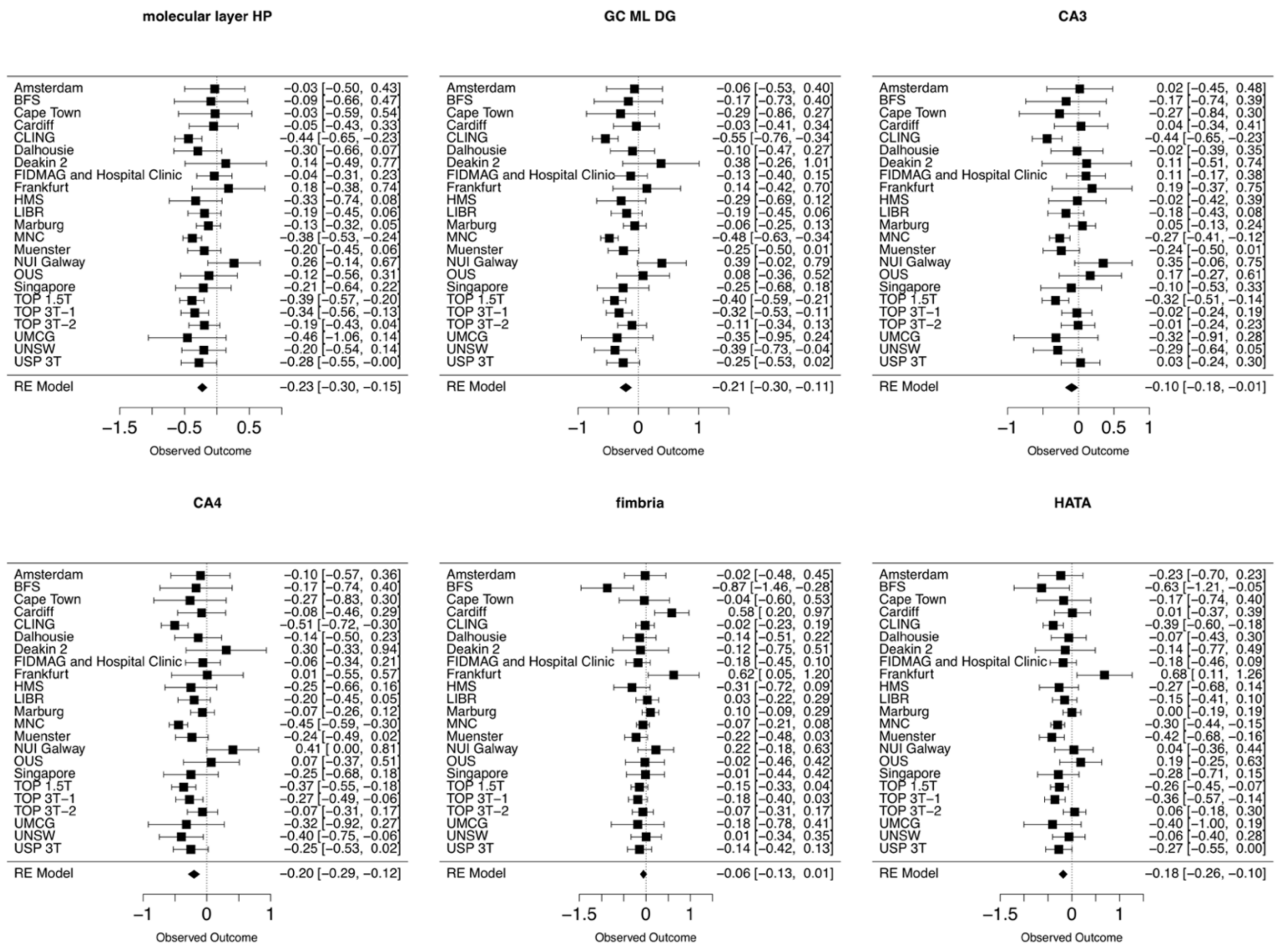

Notes: Sites/scanners that did not acquire healthy control data are not represented in these figures. CA3 implies CA2/3. *Abbreviations:* CA – cornu ammonis; GC ML DG - granule cell layer of dentate gyrus; HATA - hippocampal amygdala transition area; HP - hippocampus.

Figure S7-a: Forest plots with site-specific hippocampal subfield volume differences between bipolar disorder 1 patients and healthy controls.

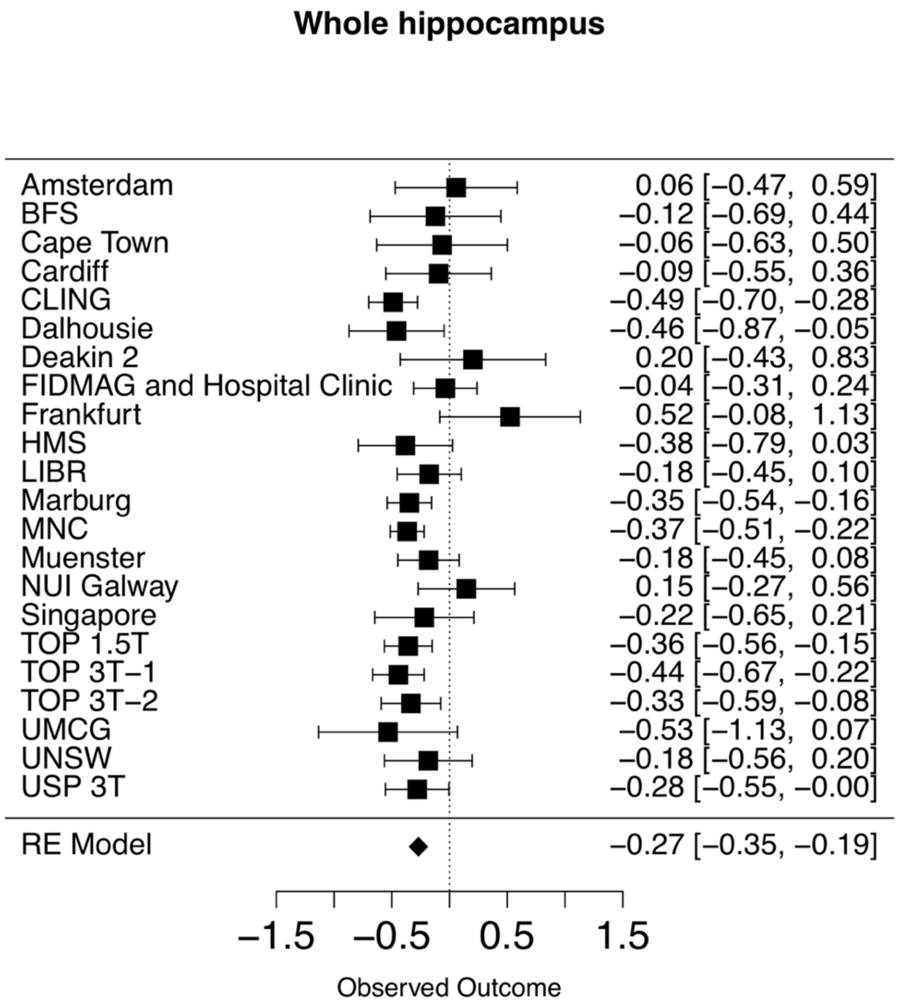

Notes: Sites/scanners that did not acquire healthy control data are not represented in these figures.

Figure S7-b: Forest plots with site-specific hippocampal subfield volume differences between bipolar disorder 1 patients and healthy controls.

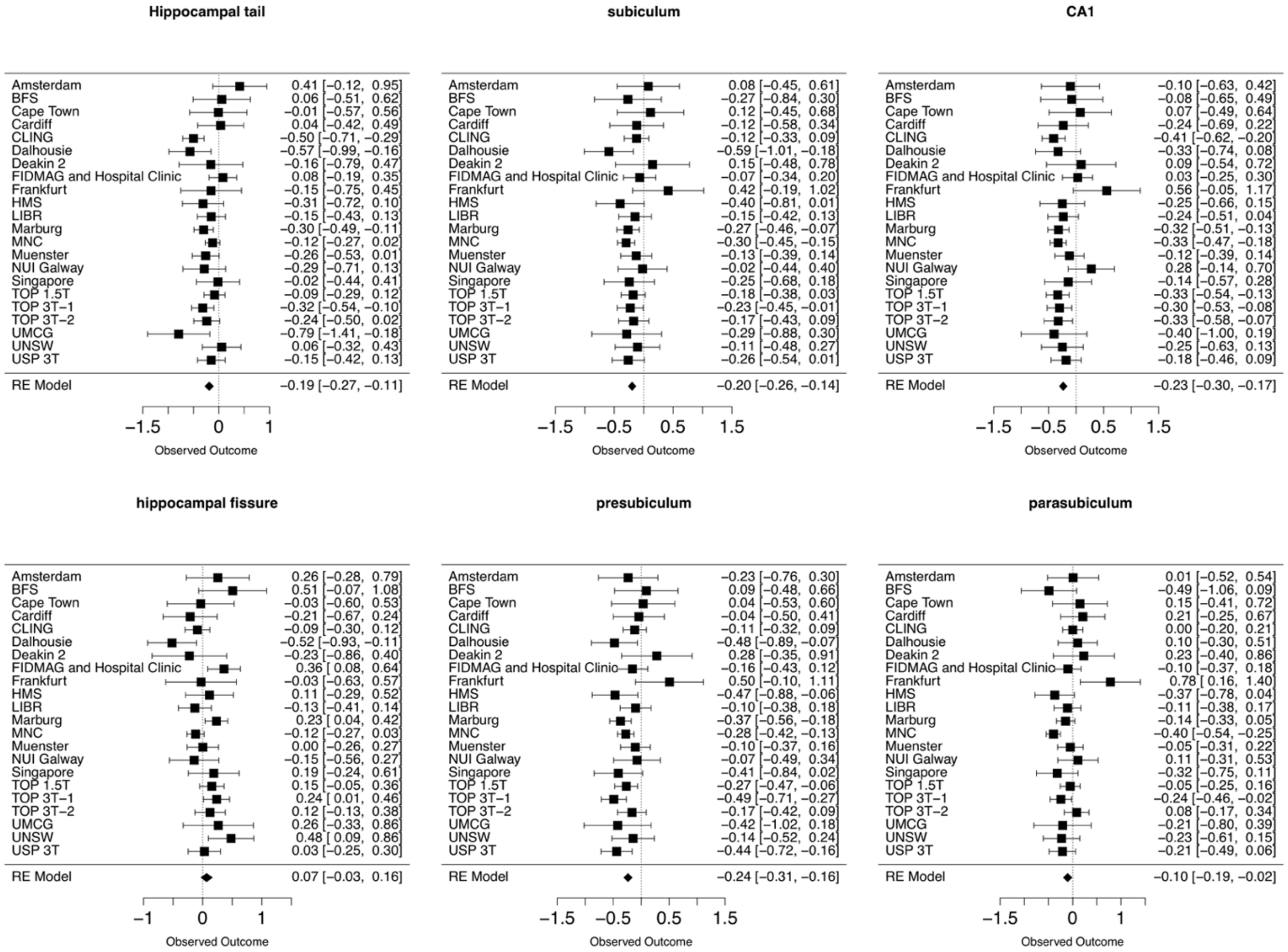

Notes: Sites/scanners that did not acquire healthy control data are not represented in these figures. *Abbreviations:* CA – cornu ammonis.

Figure S7-c: Forest plots with site-specific hippocampal subfield volume differences between bipolar disorder 1 patients and healthy controls.

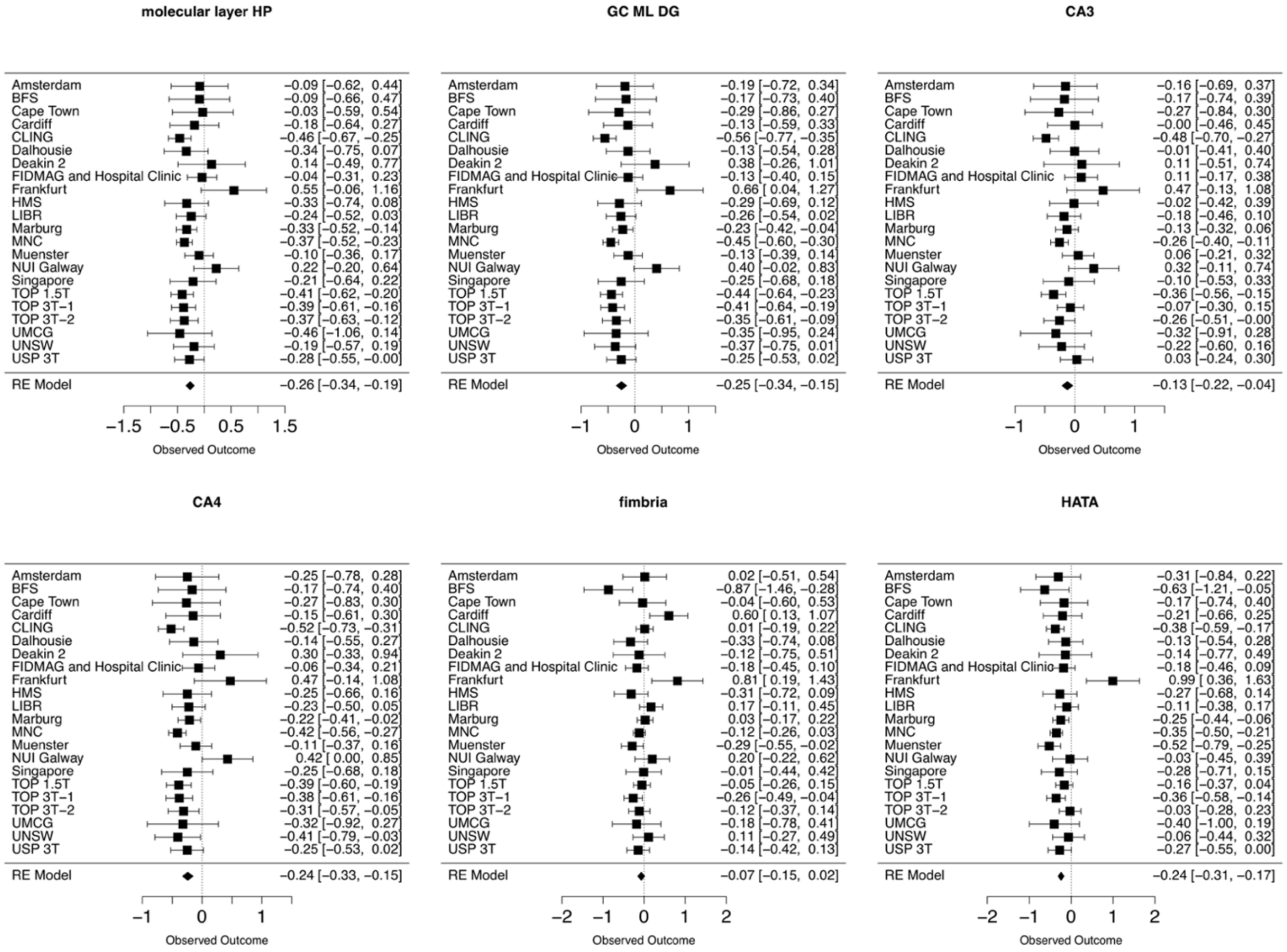

Notes: Sites/scanners that did not acquire healthy control data are not represented in these figures. CA3 implies CA2/3. *Abbreviations:* CA – cornu ammonis; GC ML DG - granule cell layer of dentate gyrus; HATA - hippocampal amygdala transition area; HP - hippocampus.

Figure S8-a: Forest plots with site-specific hippocampal subfield volume differences between bipolar disorder 2 patients and healthy controls.

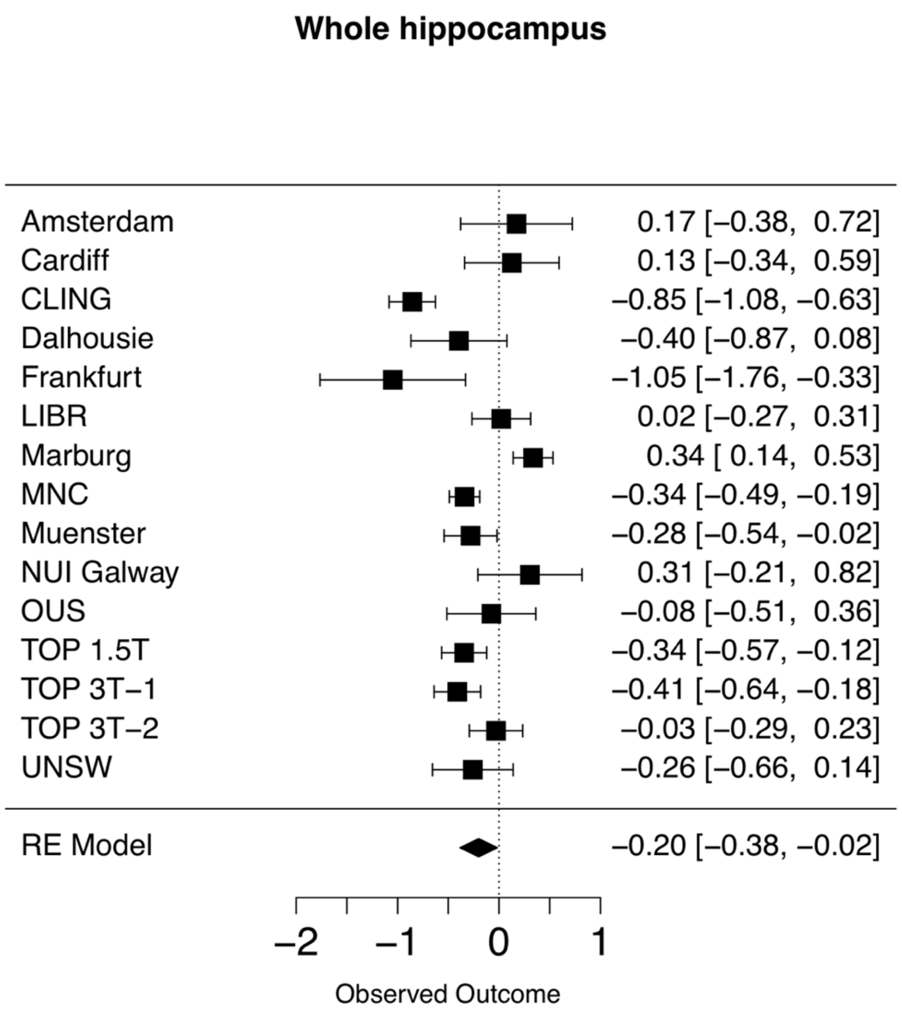

Notes: Sites/scanners that did not acquire healthy control data are not represented in these figures.

Figure S8-b: Forest plots with site-specific hippocampal subfield volume differences between bipolar disorder 2 patients and healthy controls.

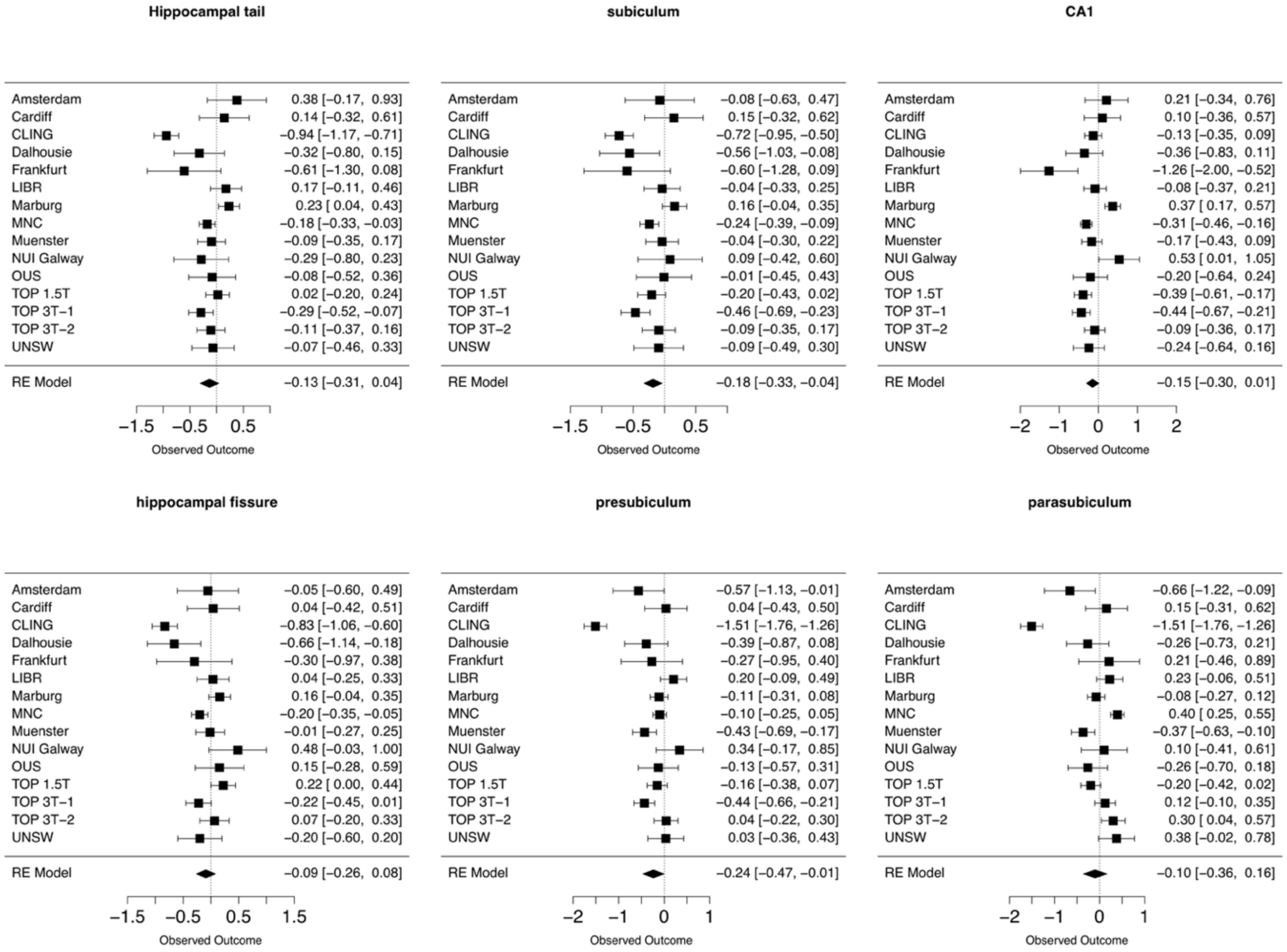

Notes: Sites/scanners that did not acquire healthy control data are not represented in these figures. *Abbreviations:* CA – cornu ammonis.

Figure S8-c: Forest plots with site-specific hippocampal subfield volume differences between bipolar disorder 2 patients and healthy controls.

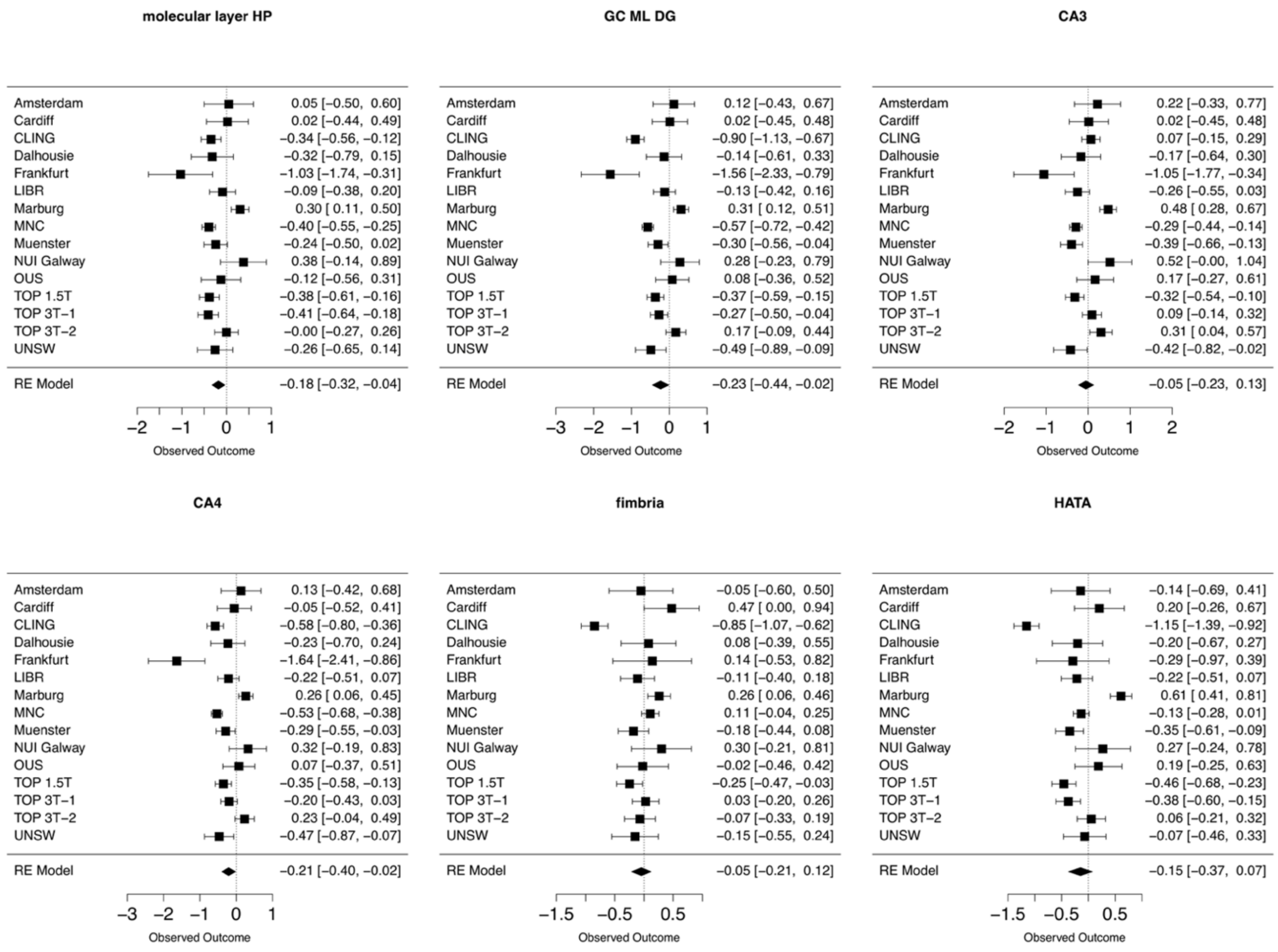

Notes: Sites/scanners that did not acquire healthy control data are not represented in these figures. CA3 implies CA2/3. *Abbreviations:* CA – cornu ammonis; GC ML DG - granule cell layer of dentate gyrus; HATA - hippocampal amygdala transition area; HP - hippocampus.

Figure S9: Left and right hippocampal subfield volume differences between bipolar disorder patients with and without a diagnosis of lifetime psychosis, and healthy controls (reference).

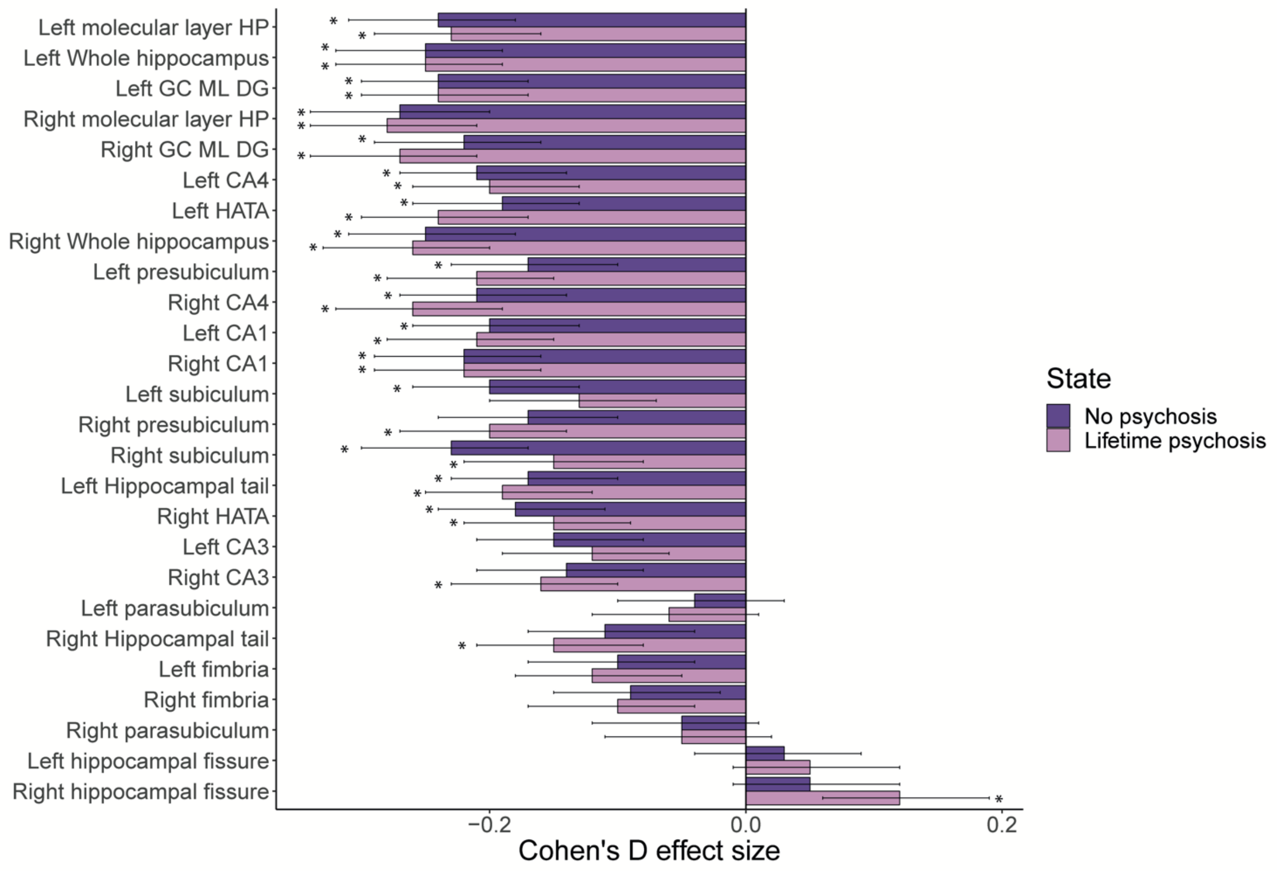

Notes: Effect sizes are ordered based on ranked effect sizes (rounded to two decimal points) from **Figure S5-a**, i.e. all patients compared to controls. Significant differences indicated by *. CA3 implies CA2/3. *Abbreviations:* CA – cornu ammonis; GC ML DG - granule cell layer of dentate gyrus; HATA - hippocampal amygdala transition area; HP - hippocampus.

Figure S10: Left and right hippocampal subfield volume differences between lithium users and non-users among bipolar disorder 1 patients, and healthy controls (reference).

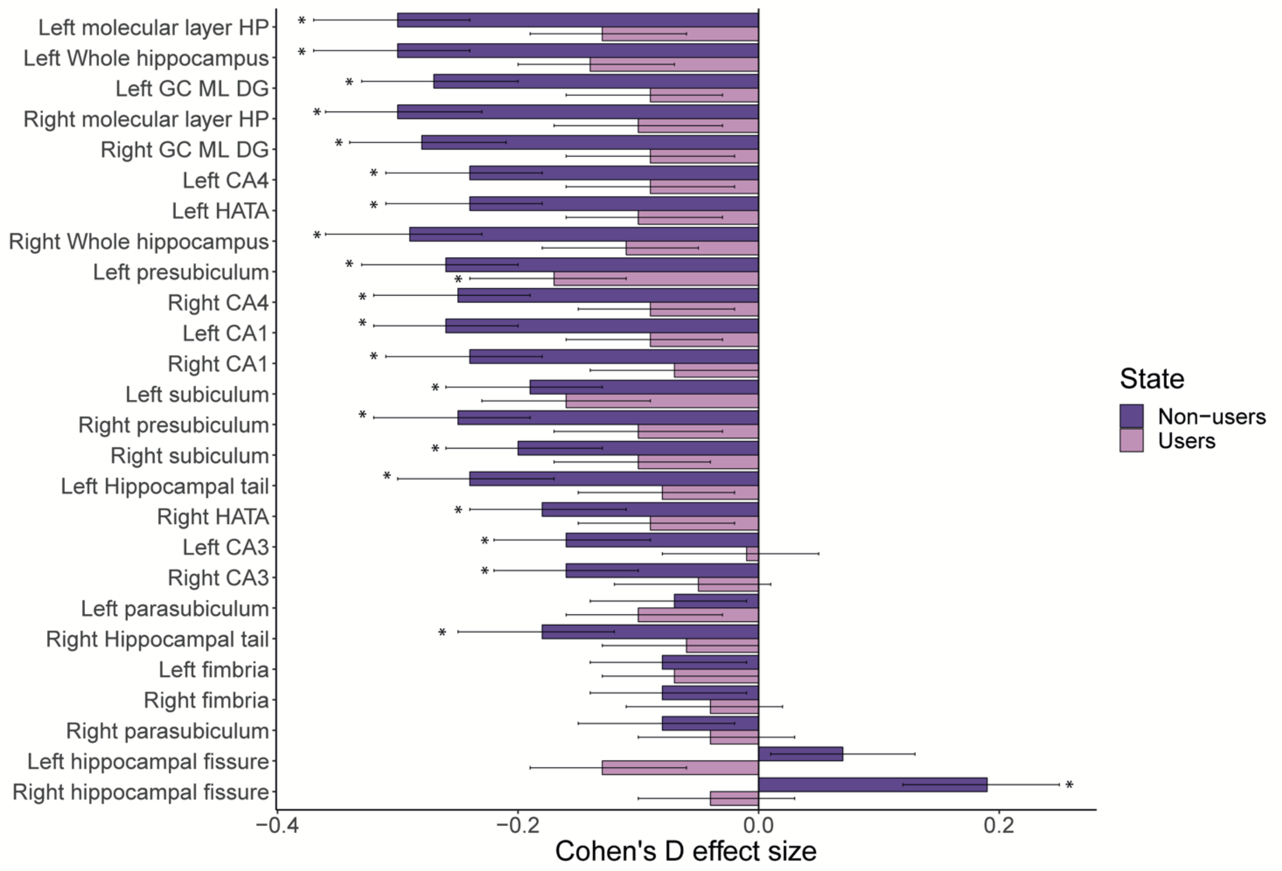

Notes: Effect sizes are ordered based on ranked effect sizes (rounded to two decimal points) from **Figure S5-a**, i.e. all patients compared to controls. Significant differences indicated by *. CA3 implies CA2/3. *Abbreviations:* CA – cornu ammonis; GC ML DG - granule cell layer of dentate gyrus; HATA - hippocampal amygdala transition area; HP - hippocampus.

Figure S11: Effects of antipsychotic, antiepileptic and antidepressant medication on the hippocampal subfield volumes in bipolar disorder 1 (with controls as reference).

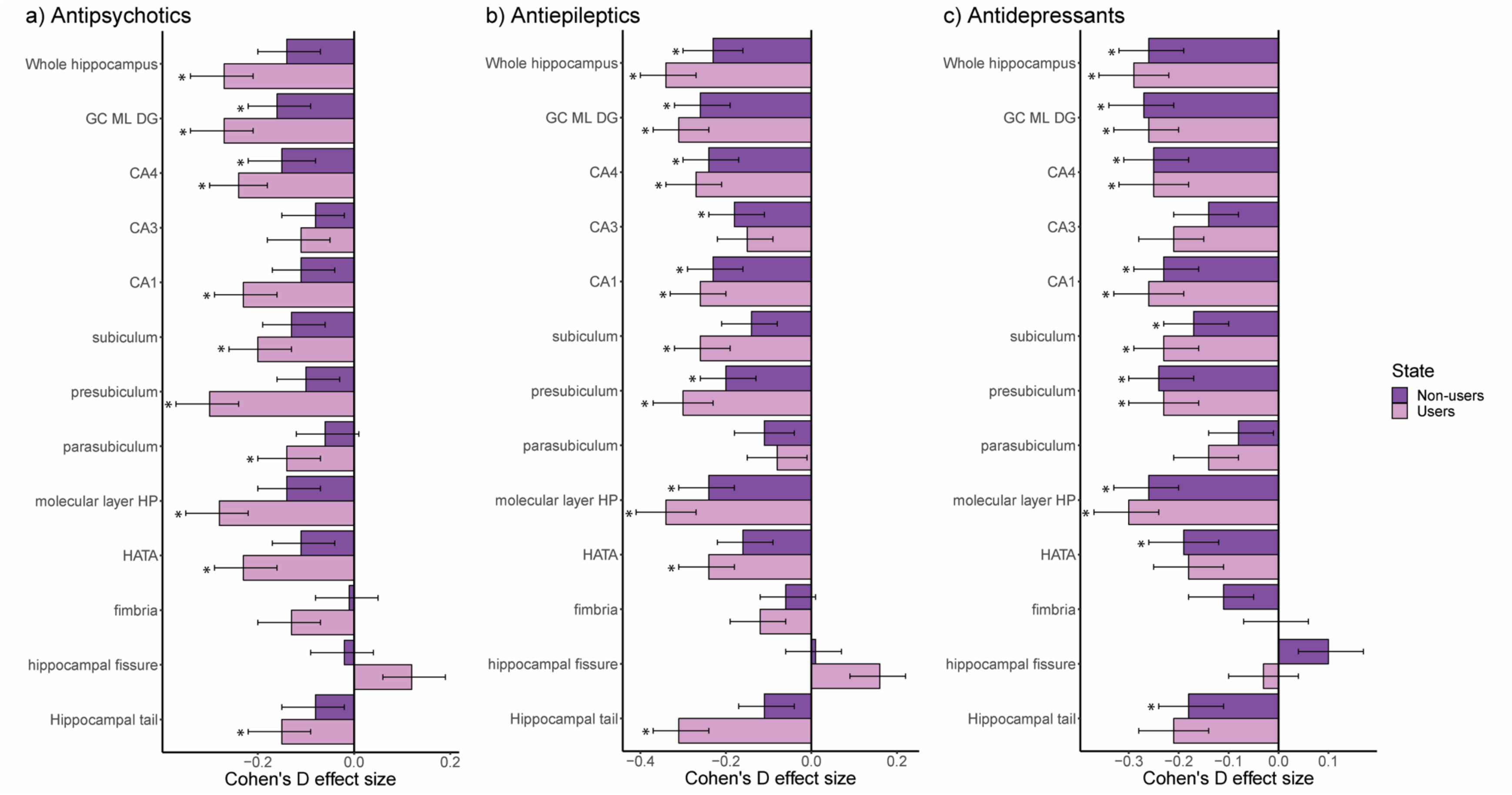

Notes: Significant differences indicated by *. CA3 implies CA2/3. *Abbreviations:* CA – cornu ammonis; GC ML DG - granule cell layer of dentate gyrus; HATA - hippocampal amygdala transition area; HP - hippocampus.

Figure S12-a: Left and right hippocampal subfield volume differences between antipsychotic users and non-users among bipolar disorder 1 patients, and healthy controls (reference).

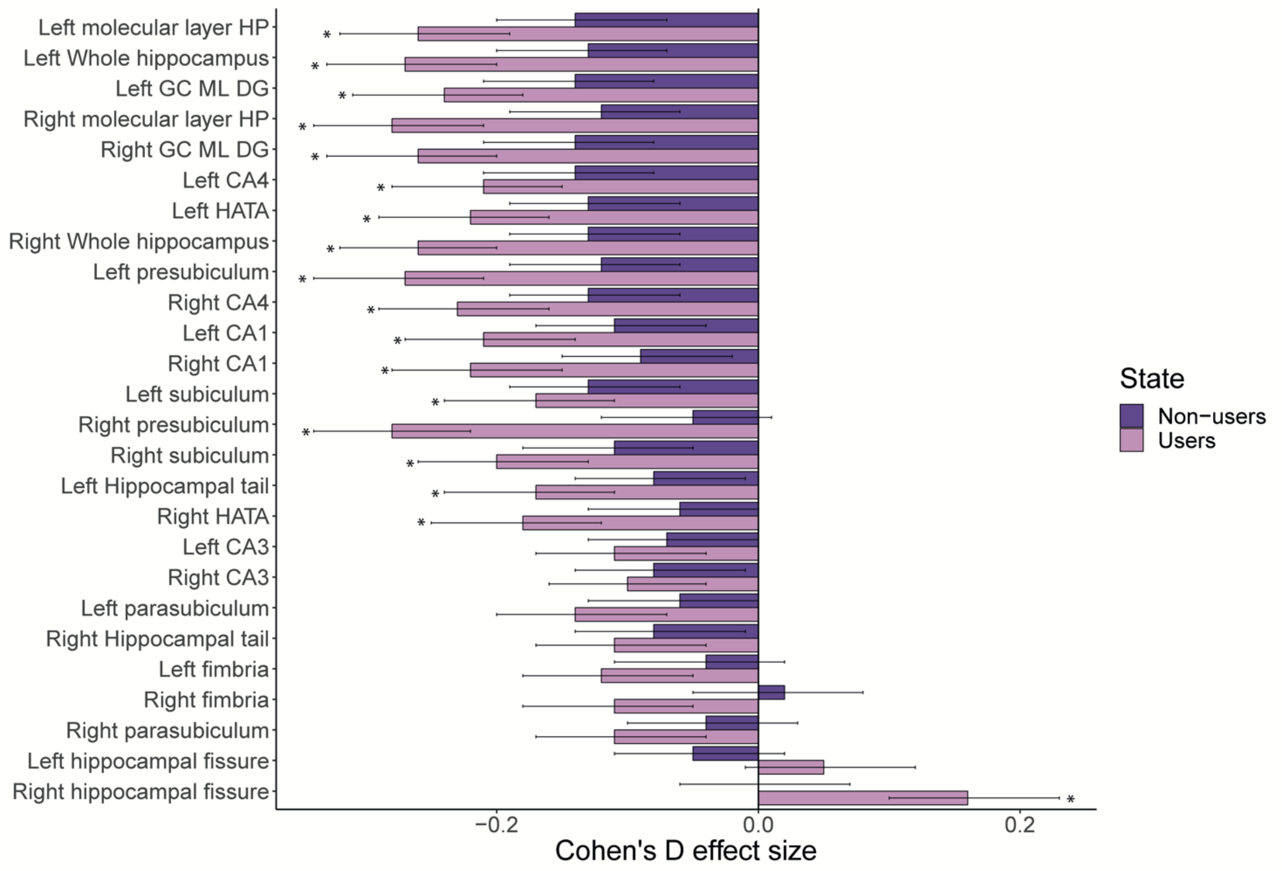

Notes: Effect sizes are ordered based on ranked effect sizes (rounded to two decimal points) from **Figure S5-a**, i.e. all patients compared to controls. Significant differences indicated by *. CA3 implies CA2/3. *Abbreviations:* CA – cornu ammonis; GC ML DG - granule cell layer of dentate gyrus; HATA - hippocampal amygdala transition area; HP - hippocampus.

Figure S12-b: Left and right hippocampal subfield volume differences between antiepileptic users and non-users among bipolar disorder 1 patients, and healthy controls (reference).

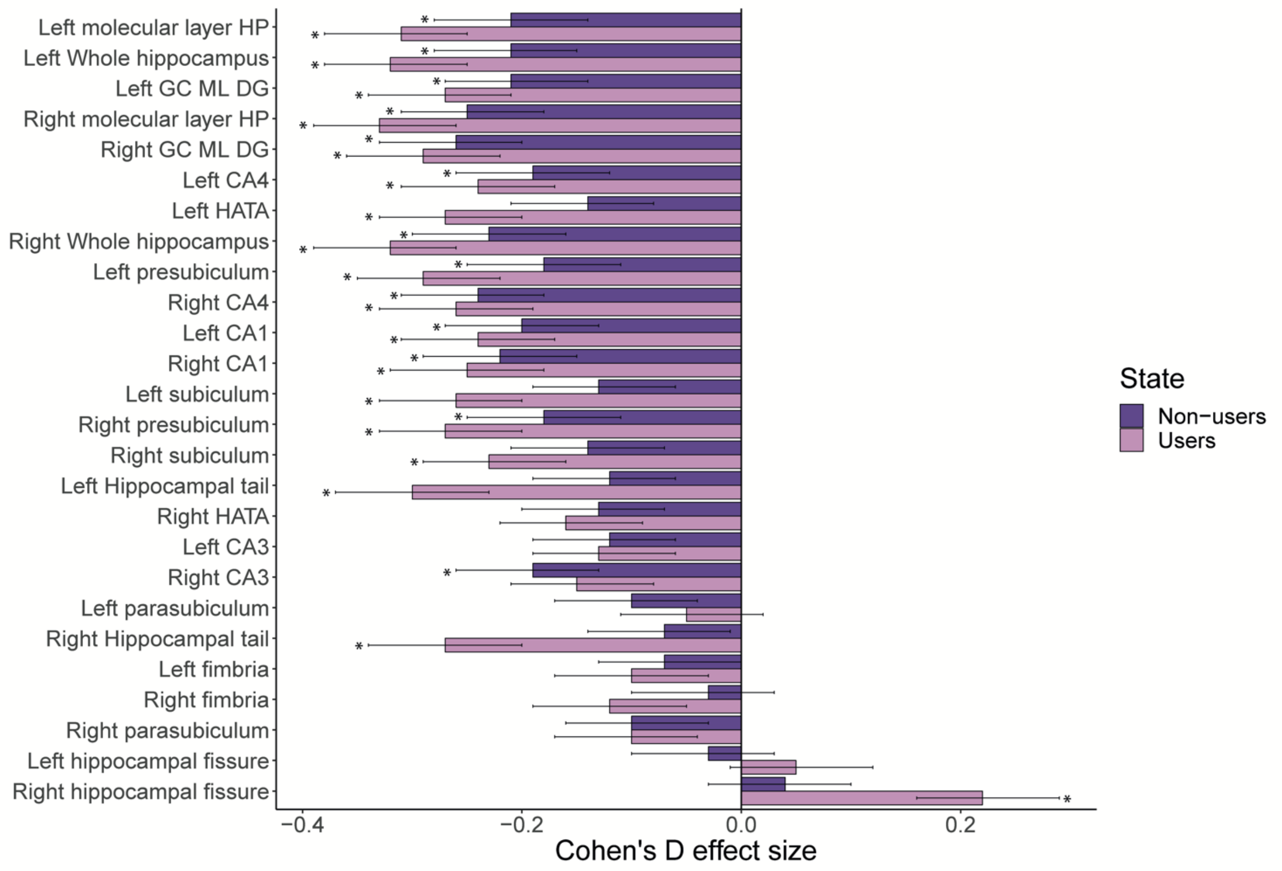

Notes: Effect sizes are ordered based on ranked effect sizes (rounded to two decimal points) from **Figure S5-a**, i.e. all patients compared to controls. Significant differences indicated by *. CA3 implies CA2/3. *Abbreviations:* CA – cornu ammonis; GC ML DG - granule cell layer of dentate gyrus; HATA - hippocampal amygdala transition area; HP - hippocampus.

Figure S12-c: Left and right hippocampal subfield volume differences between antidepressant users and non-users among bipolar disorder 1 patients, and healthy controls (reference).

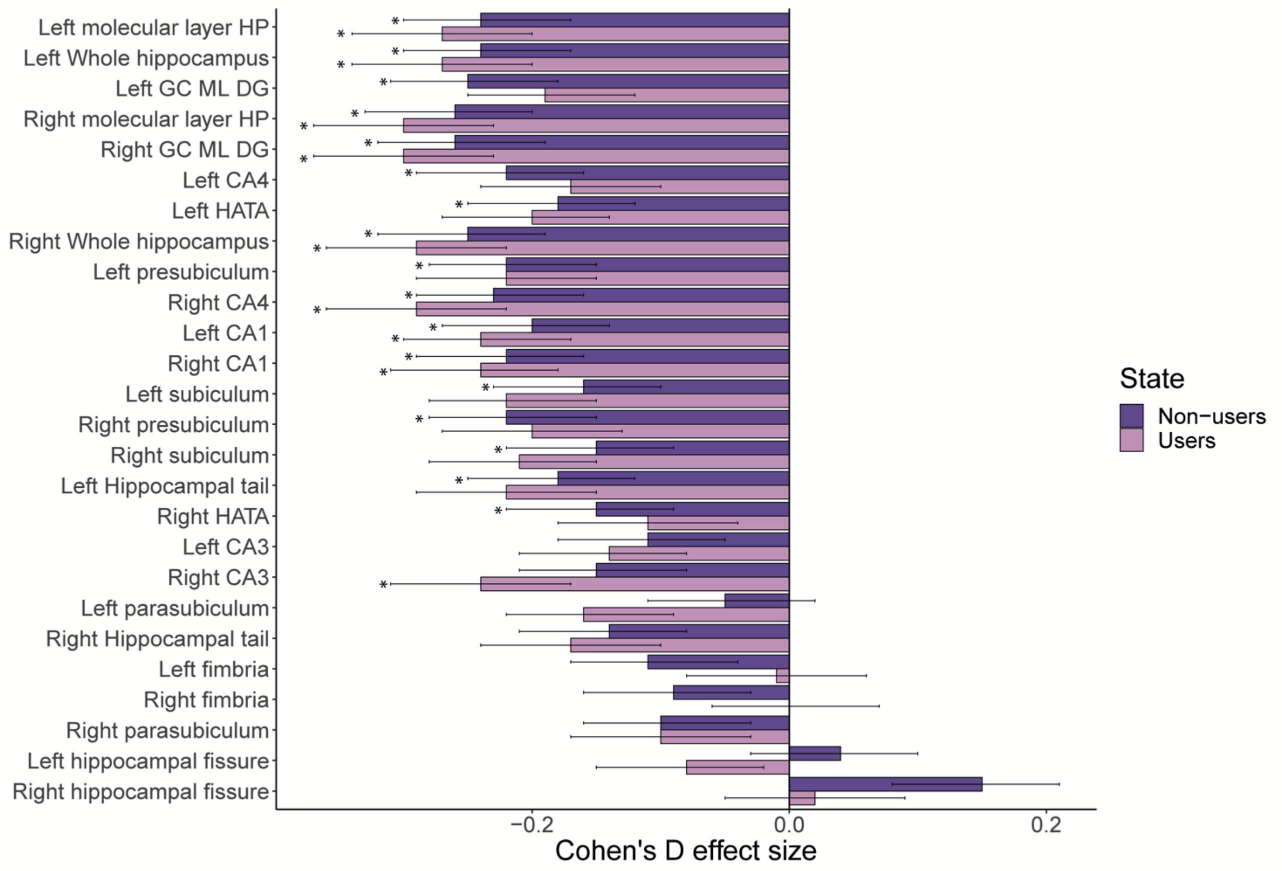

Notes: Effect sizes are ordered based on ranked effect sizes (rounded to two decimal points) from **Figure S5-a**, i.e. all patients compared to controls. Significant differences indicated by *. CA3 implies CA2/3. *Abbreviations:* CA – cornu ammonis; GC ML DG - granule cell layer of dentate gyrus; HATA - hippocampal amygdala transition area; HP - hippocampus.

Figure S13: Medication use combinations among Bipolar 1 patients with known medication status for all four medication groups.

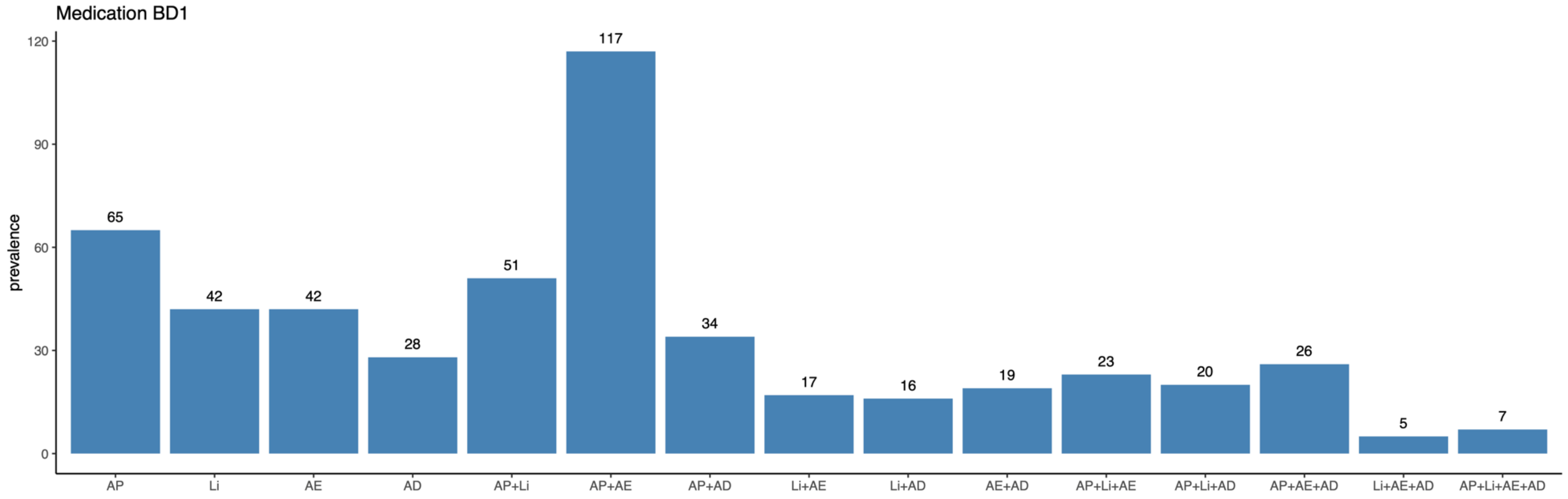

Abbreviations: AP: Antipsychotics, Li: Lithium, AE: Antiepileptics, AD: Antidepressants.

Supplemental Tables

Table S1: Demographics for the 23 sites/27 scanners of the study.

Site / scanner	N Total	N BD	N HC	N BD1/ BD2/ BD-NOS	F BD N (%)	F HC N (%)	Age BD	Age HC	DOI	PANSS POS	PANSS NEG	SANS	SAPS
**NUI Galway**	97	43	54	36/7/0	20 (46.5)	30 (55.6)	43.28±12.43	40.22±13.6					
**Singapore**	86	42	44	42/0/0	24 (57.1)	23 (52. 7)	34.81±10.85	34.07±10.86	3.67±4.54	8.5±2.83	7.07±0.34		
**Groningen**	46	22	24	22/0/0	12 (54.6)	12 (50.0)	44.5±10.52	38.49±16.35	24.23±12.1				
**Cape Town**	50	22	28	22/0/0	10 (45.5)	13 (46.4)	30.14±4.89	27.32±4.55	8.53±5.24	8.86±4.32	8.91±3.01		
**TOP 1.5T**	441	187	254	113/64/9	110 (58.8)	114 (44.9)	35.03±11.32	34.93±9.53	12.76±9.67	10.06±3.65	10.11±3.62		
**TOP 3T-1**	340	58	282	36/18/4	32 (55.2)	115 (40.8)	31.81±11.76	31.95±7.49	10.69±9.43	9.19±2.81	9.45±3.29		
**TOP 3T-2**	274	84	190	46/33/5	51 (60.7)	88 (46.3)	33.09±11.01	35.03±9.42	13.79±8.47	9.43±2.86	10.17±2.87		
**Cardiff**	110	70	40	36/33/0	45 (64.3)	25 (62.5)	43.46±6.64	43.28±5.39	23.91±8.41				
**Frankfurt**	51	20	31	14/5/0	9 (45.0)	16 (51.6)	34.8±10.17	39.19±10.7	7.7±5.9				
**LIBR**	240	84	156	46/30/8	68 (81.0)	98 (62.8)	40.52±11.7	35.68±11.46	22.62±10.33				
**Dalhousie**	116	65	51	44/21/0	44 (67.7)	31 (60. 8)	46.16±12.1	28.52±10.26	24.08±11.81				
**CLING**	359	36	323	34/1/1	21 (58.3)	191 (59.1)	40.64±10.54	25.18±5.28	14.97±9.07				
**HMS**	96	41	55	41/0/0	20 (48. 8)	34 (61.8)	43.24±12.2	39.56±12.17	13.9±10.58	7.89±1.49	9.21±4.95		

**FIDMAG & Hospital Clinic**	207	96	111	96/0/0	53 (55.2)	57 (51.4)	41.65±9.58	41.22±9.6	15.82±9.74	7.45±0.98	9.08±2.89		
**USP 3T**	207	126	81	126/0/0	83 (65.9)	41 (50.6)	32.31±9.46	28.2±7.85	7.07±6.09				
**Amsterdam**	73	36	37	20/16/0	22 (61.1)	25 (67.6)	41.61±10.79	40.19±10.62	19.06±10.82				
**Deakin-2**	41	14	27	14/0/0	4 (28.6)	16 (59.3)	21.86±2.07	21.67±1.98					
**Deakin-1**	18	18	0	18/0/0	3 (16. 7)		21.56±2.18						
**UNSW**	134	58	76	33/24/0	42 (72.4)	41 (54.0)	25.21±3.54	22.83±3.28					
**BFS**	50	25	25	25/0/0	16 (64.0)	14 (56.0)	46.32±5.03	43.74±5.09		7.64±1.44	7.12±0.33		
**Medellín/GIPSI**	75	75	0	69/6/0	49 (65.3)		42.2±11.53		19.76±11.09				
**OUS**	82	39	43	0/39/0	29 (74.4)	25 (58.1)	34.85±7.52	31.33±9.17	18.74±6.43				
**Milan 3T**	63	63	0	46/9/8	39 (61.9)		43.22±12.7		16.2±11.28				
**Milan 1.5T**	22	22	0	18/3/1	12 (54.6)		44.27±11.83		18.9±7.4				
**Marburg**	441	49	392	34/15/0	31 (63.3)	246 (62.8)	43.65±10.66	34.7±12.82	19.6±12.18			1.58±2.62	0.33±0.86
**Muenster**	237	24	213	8/16/0	7 (29.2)	142 (66.7)	42.46±12.91	28.21±10.16	16.33±12.1			3.04±3.33	0.54±0.98
**MNC**	742	53	689	40/13/0	28 (52.8)	396 (57.5)	38.06±11.78	35.49±12.05	12.25±9.62				

Abbreviations: BD: bipolar disorder, HC: healthy control, DOI: duration of illness, F: female, PANSS: Positive and Negative Syndrome Scale, PANSS POS: Total PANSS positive score, PANSS NEG: Total PANSS negative score, SANS: Scale for the Assessment of Negative Symptoms, SAPS: Scale for the Assessment of Positive Symptoms.

Table S2: Image acquisition parameters for the 23 sites/27 scanners of the study.

Site/ scanner	Sequence	Field strength	Acq. dir.	# of Slices	Slice gap	Voxel size (mm^3)	TI	TE	TR	Flip angle	Citation
**NUI Galway**	3D T1-weighted turbo field sequence	3T Philips Achieva	Axial	256	0mm	1×1×1	600ms	3.9ms	8.5ms	15	
**Singapore**	3D T1-weighted magnetization prepared rapid acquisition gradient echo (MPRAGE)	3T Philips Achieva	Axial	180	0mm	1×1×1		3.3ms	7.2ms	8	
**Groningen**	3D T1-weighted ultrafast spoiled gradient echo sequence (SPGR)	3T Philips	Axial			.9×.9×1.2		4.59ms	9.8ms	8	[Haarman et al., 2016]
**Cape Town**	3D T1-weighted magnetization prepared rapid acquisition using multiple gradient echos (MEMPRAGE)	3T Siemens Allegra	Sagittal	128	0mm	1.3×1×1.3	1100ms	1.53ms; 3.21ms; 4.89ms; 6.57ms		7	[van der Kouwe et al., 2008]
**TOP 1.5T**	3D T1-weighted magnetization prepared rapid acquisition gradient echo (MPRAGE)	1.5T Siemens Sonata	Sagittal	160	0mm	1.3×.9×1	1000ms	3.93ms	2730ms	7	[Haukvik et al., 2015]
**TOP 3T - 1**	3D T1-weighted fast spoiled gradient echo (FSPGR) sequence	3T General Electric (GE) Healthcare Signa HDxt	Sagittal	166		1×1×1.2	450ms	Min Full	7.8s	12	[Gurholt et al., 2018]

**TOP 3T - 2**	3D T1-weighted BRAVO sequence	3T GE Discovery MR 750	Sagittal	192		1×1×1		3.18	450	12	[Tesli et al., 2020]
**Cardiff**	3D T1-weighted fast spoiled gradient recall (3D FSPGR)	3T GE HDx	Axial	172	0mm	1×1×1	450ms	3ms	7.9ms	20	
**Frankfurt**	3D Modified Driven Equilibrium Fourier Transform (MDEFT)-sequence	3T Siemens Magnetom Allegra 3 Tesla MRI	Sagittal	176	0mm	1×1×1		2.4ms	7.92ms	15	
**LIBR**	3D T1-weighted magnetization prepared rapid acquisition gradient echo (MPRAGE)	3T GE MR750	Axial	186	0.9mm	.9×.9×.9	725ms	2.008; 2.012	5.952; 5.96; 5.992; 6.008	8	
**Dalhousie**	T1-weighted spoiled gradient recall scan	1.5T GE Signa	Coronal	124	0mm	1×1×1.5		5ms	25ms	40	[Van Gestel et al., 2019]
**CLING**	3D T1-weighted magnetization prepared rapid acquisition gradient echo (MPRAGE)	3T Siemens Tim Trio	Sagittal	176	0mm	1×1×1	900ms	3.26ms	2250ms	9	
**HMS**	3D T1-weighted magnetization prepared rapid acquisition gradient echo (MPRAGE)	1.5T Siemens Sonata	Sagittal	176	0mm	1×1×1	700ms	3.42ms	1900ms	15	
**FIDMAG & Hospital Clinic**	3D T1-weighted enhanced fast	1.5T GE Signa	Axial	180	0mm	.5×.5×1	710ms	3.93ms	2000ms	15	

	gradient echo (EFGRE 3D)										
**USP 3T**	3D T1-weighted fast-field echo sequence	3T Philips Intera Achieva	Sagittal			1×1×1	900ms	3.2ms	7ms	8	[Soeiro-de-Souza et al., 2017]
**Amsterdam**	3D T1-weighted turbo field echo (TFE)	3T scanner Philips Gyroscan Intera	Coronal	182	0mm	1×1×1.2	0ms	4.6ms	9.6ms	8	
**Deakin-2**	3D T1-weighted	3T Siemens TrioTim	Sagittal			.9×.9×.9	900ms	2.24ms		9	
**Deakin-1**	3D T1-weighted	3T Siemens TrioTim	Sagittal			.5×.5×.9	900ms	2.24ms		9	
**UNSW**	3D T1-weighted sequence	3T GE MR750	Sagittal	180	0mm	1×1×1	0ms	2.5ms	5.5ms	8	[Roberts et al., 2016]
**BFS**	3D T1-weighted magnetization prepared rapid acquisition gradient echo (MPRAGE)	1.5T GE Signa Horizon HDX	Coronal	180	0mm	1.3×1.3×1.2	500ms	4ms	9.7ms	8	[Sprooten et al., 2011]
**Medellín/ GIPSI**	T1-weighted	3T Philips Ingenia	Axial	160	1mm	1×1×1		2.1	4.9	8	
**OUS**	3D T1-weighted turbo field echo (TFE)	3T Philips Achieva	Sagittal	220	0mm	1×1×1		2.3ms	8.4ms	7	
**Milan 3T**	3D T1-weighted TFE	3T Philips Achieva	Sagittal	185	0mm	.9×.9×1		4.6ms	9.8ms	8	
**Milan 1.5T**	3D T1-weighted TFE	1.5T Philips Achieva	Sagittal	162	0mm	1×1×1		3.2ms	7.2ms	8	
**Marburg**	3D T1-weighted magnetization prepared rapid acquisition gradient echo (MPRAGE)	3T Siemens Magnetom TiroTim syngo	Sagittal	176	0.5mm	1×1×1	900ms	2.26ms	1900ms	9	[Vogelbacher et al., 2018]

**Muenster**	3D T1-weighted magnetization prepared rapid acquisition gradient echo (MPRAGE)	3T Siemens PRISMA	Sagittal	192	0 mm	1×1×1	900ms	2.28ms	2130ms	8	[Vogelbacher et al., 2018]
**MNC**	3D fast gradient echo sequence	3T scanner Gyroscan Intera, Philips Medical Systems	Sagittal	320	0mm	.5×.5×.5	814.5ms	3.4ms	7.4ms	9	[Dannlowski et al., 2015]

Abbreviations: Acq. dir. – acquisition direction; TE – Echo time; TI – Inversion time; TR – Repetition time.

Table S3: Demographic and clinical information for the bipolar 1 subgroup and controls.

	**Cases (N = 1079)**	**Controls (N = 3226)**	**χ^2-test/Wilcoxon rank-sum test**	**p-value**
N Females	627 (58.1%)	1793 (55.6%)	2	0.1572
Age†	38.3±11.9	33.3±11.2	5.0	7.7e−36
AAO‡	24.3±9.3			
DOI‡	14.3±10.7			
N Lifetime psychosis§	347/191/541			
PANSS Positive¶	9±3.2			
PANSS Negative¶	9.2±3.4			
**Medication**				
N Lithium§	319/464/296			
N Antipsychotics§	472/385/222			
N Antiepileptics§	256/309/514			
N Antidepressants§	155/410/514			

Abbreviations: AAO: age at onset, BD: bipolar disorder, CTR: controls, DOI: duration of illness, NOS: not otherwise specified, PANSS: Positive and Negative Syndrome Scale.

† Not normal - applied two-sided Wilcoxon rank sum test.

‡ 169 patients with missing AAO/DOI.

§ Yes/No/Missing.

¶ 701 patients missing PANSS score.

Table S4: Hippocampal subfield volumes in bipolar disorder patients compared to controls (reference).

	Combined structures				Left hemisphere				Right hemisphere			
**Structure**	**d**	**L CI**	**U CI**	**p-value**	**d**	**L CI**	**U CI**	**p-value**	**d**	**L CI**	**U CI**	**p-value**
**Hippocampal tail**	−0.1	−0.16	−0.04	**0.0012**	−0.12	−0.17	−0.06	**2e−04**	−0.07	−0.13	−0.02	0.0182
**Subiculum**	−0.15	−0.21	−0.1	**1.1e−06**	−0.15	−0.21	−0.09	**1.9e−06**	−0.14	−0.2	−0.09	**5.3e−06**
**CA1**	−0.18	−0.23	−0.12	**2.8e−08**	−0.16	−0.22	−0.1	**3.3e−07**	−0.16	−0.22	−0.1	**3.1e−07**
**Hippocampal fissure**	0.07	0.01	0.12	0.0342	0.03	−0.02	0.09	0.2828	0.08	0.03	0.14	0.0076
**Presubiculum**	−0.18	−0.23	−0.12	**2.6e−08**	−0.17	−0.23	−0.12	**3.4e−08**	−0.15	−0.2	−0.09	**3.1e−06**
**Parasubiculum**	−0.06	−0.12	0	0.0574	−0.07	−0.13	−0.01	0.0234	−0.03	−0.09	0.02	0.2865
**Molecular layer HP**	−0.21	−0.27	−0.15	**4.0e−11**	−0.2	−0.26	−0.14	**3.6e−10**	−0.19	−0.25	−0.14	**7.6e−10**
**GC ML DG**	−0.21	−0.26	−0.15	**5.6e−11**	−0.19	−0.25	−0.14	**1.0e−09**	−0.19	−0.25	−0.13	**2.4e−09**
**CA2/3**	−0.11	−0.16	−0.05	**8e−04**	−0.1	−0.16	−0.04	**0.0013**	−0.09	−0.15	−0.03	0.0047
**CA4**	−0.19	−0.25	−0.14	**1.1e−09**	−0.18	−0.23	−0.12	**2.0e−08**	−0.17	−0.23	−0.12	**3.3e−08**
**Fimbria**	−0.07	−0.12	−0.01	0.0369	−0.06	−0.12	0	0.0541	−0.05	−0.11	0	0.0876
**HATA**	−0.17	−0.23	−0.11	**7.5e−08**	−0.18	−0.23	−0.12	**2.6e−08**	−0.12	−0.18	−0.07	**1e−04**
**Whole hippocampus**	−0.2	−0.26	−0.14	**3.1e−10**	−0.2	−0.26	−0.14	**3.7e−10**	−0.18	−0.24	−0.12	**8.7e−09**

Notes: Combined structures imply left and right hemisphere combined. Significant differences indicated in bold. *Abbreviations:* CI – confidence interval; CA – cornu ammonis; d – Cohen's d; GC ML DG - granule cell layer of dentate gyrus; HATA - hippocampal amygdala transition area; HP – hippocampus; L CI – Lower CI; U CI – Upper CI.

Table S5: Hippocampal subfield volumes in bipolar disorder 1 compared to bipolar disorder 2 patients (reference).

	**Combined structures**				**Left structures**				**Right structures**			
**Structure**	**Cohen's d**	**Lower CI**	**Upper CI**	**p-value**	**Cohen's d**	**Lower CI**	**Upper CI**	**p-value**	**Cohen's d**	**Lower CI**	**Upper CI**	**p-value**
**Hippocampal tail**	−0.14	−0.25	−0.04	0.0202	−0.12	−0.22	−0.02	0.0558	−0.15	−0.26	−0.05	0.0143
**Subiculum**	−0.06	−0.16	0.05	0.3534	−0.06	−0.17	0.04	0.3195	−0.04	−0.15	0.06	0.4686
**CA1**	−0.09	−0.19	0.02	0.1657	−0.12	−0.22	−0.01	0.064	−0.04	−0.15	0.06	0.4935
**Hippocampal fissure**	0	−0.1	0.1	0.9903	−0.05	−0.15	0.06	0.4349	0.04	−0.06	0.15	0.4691
**Presubiculum**	−0.14	−0.25	−0.04	0.0199	−0.18	−0.28	−0.07	0.0041	−0.08	−0.19	0.02	0.1795
**Parasubiculum**	−0.14	−0.24	−0.03	0.0264	−0.09	−0.19	0.02	0.1571	−0.16	−0.27	−0.06	0.0085
**Molecular layer HP**	−0.09	−0.2	0.01	0.1293	−0.1	−0.2	0.01	0.114	−0.08	−0.18	0.02	0.1996
**GC ML DG**	−0.06	−0.17	0.04	0.3041	−0.05	−0.15	0.06	0.4657	−0.07	−0.18	0.03	0.2446
**CA2/3**	−0.04	−0.14	0.07	0.5692	0.02	−0.09	0.12	0.7897	−0.08	−0.18	0.03	0.2253
**CA4**	−0.04	−0.15	0.06	0.4709	−0.03	−0.13	0.08	0.682	−0.06	−0.16	0.05	0.3691
**Fimbria**	0.03	−0.08	0.13	0.6721	0.03	−0.07	0.13	0.6309	0.01	−0.09	0.12	0.8252
**HATA**	−0.07	−0.17	0.03	0.2611	−0.09	−0.2	0.01	0.1316	−0.03	−0.14	0.07	0.5921
**Whole hippocampus**	−0.1	−0.21	0	0.0938	−0.1	−0.2	0	0.1042	−0.1	−0.2	0	0.1054

Notes: Combined structures imply left and right hemisphere combined. Significant differences indicated in bold. *Abbreviations:* CI – confidence interval; CA – cornu ammonis; GC ML DG - granule cell layer of dentate gyrus; HATA - hippocampal amygdala transition area; HP - hippocampus.

Table S6-a: Hippocampal subfield volumes in bipolar disorder 1 and bipolar disorder 2 patients compared to controls (reference) (both hemispheres combined).

	**Bipolar disorder 1**				**Bipolar disorder 2**			
**Structure**	**Cohen's d**	**Lower CI**	**Upper CI**	**p-value**	**Cohen's d**	**Lower CI**	**Upper CI**	**p-value**
**Hippocampal tail**	−0.14	−0.2	−0.08	**6.9e−05**	0	−0.06	0.07	0.9775
**Subiculum**	−0.17	−0.23	−0.11	**1.0e−06**	−0.1	−0.16	−0.03	0.0527
**CA1**	−0.18	−0.24	−0.12	**6.1e−08**	−0.13	−0.19	−0.06	0.0106
**Hippocampal fissure**	0.07	0.01	0.13	0.0341	0.05	−0.02	0.11	0.3295
**Presubiculum**	−0.21	−0.27	−0.15	**6.9e−10**	−0.07	−0.14	−0.01	0.1525
**Parasubiculum**	−0.09	−0.15	−0.03	0.0095	0.03	−0.03	0.1	0.5269
**Molecular layer HP**	−0.23	−0.29	−0.17	**3.6e−11**	−0.14	−0.2	−0.07	0.006
**GC ML DG**	−0.23	−0.29	−0.17	**3.6e−11**	−0.14	−0.2	−0.07	0.0055
**CA2/3**	−0.11	−0.17	−0.05	**0.001**	−0.08	−0.14	−0.01	0.1108
**CA4**	−0.2	−0.26	−0.14	**1.8e−09**	−0.14	−0.21	−0.07	0.0045
**Fimbria**	−0.06	−0.12	0	0.0681	−0.05	−0.11	0.02	0.3186
**HATA**	−0.19	−0.25	−0.14	**9.9e−09**	−0.09	−0.15	−0.02	0.0709
**Whole hippocampus**	−0.22	−0.28	−0.16	**8.9e−11**	−0.11	−0.18	−0.05	0.02

Notes: Significant differences indicated in bold. *Abbreviations:* CI – confidence interval; CA – cornu ammonis; GC ML DG - granule cell layer of dentate gyrus; HATA - hippocampal amygdala transition area; HP - hippocampus.

Table S6-b: Hippocampal subfield volumes in bipolar disorder 1 and bipolar disorder 2 patients compared to controls (reference) (left hemisphere).

	**Bipolar disorder 1**				**Bipolar disorder 2**			
**Structure**	**Cohen's d**	**Lower CI**	**Upper CI**	**p-value**	**Cohen's d**	**Lower CI**	**Upper CI**	**p-value**
**Hippocampal tail**	−0.14	−0.2	−0.08	**2.5e−05**	−0.03	−0.1	0.03	0.5125
**Subiculum**	−0.16	−0.22	−0.1	**3.0e−06**	−0.1	−0.16	−0.03	0.0464
**CA1**	−0.18	−0.24	−0.12	**2.3e−07**	−0.09	−0.16	−0.03	0.0593
**Hippocampal fissure**	0.03	−0.03	0.09	0.3457	0.04	−0.03	0.1	0.4473
**Presubiculum**	−0.22	−0.28	−0.16	**1.7e−10**	−0.05	−0.11	0.02	0.3444
**Parasubiculum**	−0.09	−0.15	−0.03	0.0105	−0.01	−0.08	0.06	0.8445
**Molecular layer HP**	−0.21	−0.27	−0.15	**2.9e−10**	−0.12	−0.19	−0.06	0.0115
**GC ML DG**	−0.21	−0.27	−0.15	**9.0e−10**	−0.13	−0.2	−0.07	0.0074
**CA2/3**	−0.1	−0.16	−0.04	0.0039	−0.1	−0.17	−0.04	0.0365
**CA4**	−0.19	−0.25	−0.13	**4.1e−08**	−0.13	−0.2	−0.07	0.0068
**Fimbria**	−0.06	−0.12	0	0.0821	−0.04	−0.11	0.02	0.3866
**HATA**	−0.21	−0.27	−0.15	**1.6e−09**	−0.08	−0.14	−0.01	0.1127
**Whole hippocampus**	−0.22	−0.28	−0.16	**1.4e−10**	−0.12	−0.18	−0.05	0.0176

Notes: Significant differences indicated in bold. *Abbreviations:* CI – confidence interval; CA – cornu ammonis; GC ML DG - granule cell layer of dentate gyrus; HATA - hippocampal amygdala transition area; HP - hippocampus.

Table S6-c: Hippocampal subfield volumes in bipolar disorder 1 and bipolar disorder 2 patients compared to controls (reference) (right hemisphere).

	Bipolar disorder 1				Bipolar disorder 2			
**Structure**	**Cohen's d**	**Lower CI**	**Upper CI**	**p-value**	**Cohen's d**	**Lower CI**	**Upper CI**	**p-value**
**Hippocampal tail**	−0.11	−0.17	−0.05	**0.0011**	0.03	−0.03	0.1	0.4805
**Subiculum**	−0.16	−0.22	−0.1	**3.6e−06**	−0.09	−0.15	−0.02	0.083
**CA1**	−0.16	−0.22	−0.1	**1.7e−06**	−0.14	−0.21	−0.07	0.0047
**Hippocampal fissure**	0.09	0.03	0.15	0.0057	0.05	−0.01	0.12	0.3042
**Presubiculum**	−0.17	−0.23	−0.11	**1.1e−06**	−0.09	−0.15	−0.02	0.0847
**Parasubiculum**	−0.07	−0.13	−0.01	0.0448	0.07	0	0.13	0.1858
**Molecular layer HP**	−0.21	−0.27	−0.15	**8.0e−10**	−0.13	−0.2	−0.06	0.0084
**GC ML DG**	−0.21	−0.27	−0.15	**1.3e−09**	−0.12	−0.19	−0.05	0.0151
**CA2/3**	−0.1	−0.16	−0.04	**0.0024**	−0.04	−0.11	0.02	0.3993
**CA4**	−0.19	−0.25	−0.13	**4.2e−08**	−0.12	−0.19	−0.06	0.013
**Fimbria**	−0.05	−0.11	0.01	0.1501	−0.04	−0.11	0.02	0.3929
**HATA**	−0.14	−0.2	−0.08	**5.4e−05**	−0.08	−0.14	−0.01	0.1125
**Whole hippocampus**	−0.2	−0.26	−0.14	**2.3e−09**	−0.1	−0.17	−0.04	0.0412

Notes: Significant differences indicated in bold. *Abbreviations:* CI – confidence interval; CA – cornu ammonis; GC ML DG - granule cell layer of dentate gyrus; HATA - hippocampal amygdala transition area; HP - hippocampus.

Table S7-a: The effect of lifetime psychosis on hippocampal subfield volumes compared with controls (reference) (both hemispheres combined).

	**Without lifetime psychosis**				**With lifetime psychosis**			
**Structure**	**Cohen's d**	**Lower CI**	**Upper CI**	**p-value**	**Cohen's d**	**Lower CI**	**Upper CI**	**p-value**
**Hippocampal tail**	−0.15	−0.21	−0.08	0.0044	−0.18	−0.25	−0.11	**4e−04**
**Subiculum**	−0.23	−0.29	−0.16	**1.5e−05**	−0.15	−0.21	−0.08	**0.0038**
**CA1**	−0.23	−0.3	−0.16	**1.1e−05**	−0.24	−0.31	−0.18	**1.9e−06**
**Hippocampal fissure**	0.04	−0.02	0.11	0.4204	0.1	0.03	0.16	0.0485
**Presubiculum**	−0.18	−0.25	−0.12	**5e−04**	−0.22	−0.29	−0.16	**9.0e−06**
**Parasubiculum**	−0.05	−0.12	0.01	0.3221	−0.06	−0.13	0	0.2271
**Molecular layer HP**	−0.27	−0.34	−0.21	**1.8e−07**	−0.27	−0.33	−0.2	**1.1e−07**
**GC ML DG**	−0.25	−0.32	−0.18	**1.8e−06**	−0.28	−0.34	−0.21	**3.8e−08**
**CA2/3**	−0.16	−0.22	−0.09	**0.0024**	−0.16	−0.22	−0.09	**0.0016**
**CA4**	−0.23	−0.29	−0.16	**1.4e−05**	−0.25	−0.31	−0.18	**1.1e−06**
**Fimbria**	−0.11	−0.17	−0.04	0.0408	−0.12	−0.19	−0.06	0.0137
**HATA**	−0.21	−0.27	−0.14	**6.5e−05**	−0.22	−0.29	−0.16	**1.2e−05**
**Whole hippocampus**	−0.26	−0.33	−0.2	**4.9e−07**	−0.27	−0.34	−0.2	**9.9e−08**

Notes: Significant differences indicated in bold. *Abbreviations:* CI – confidence interval; CA – cornu ammonis; GC ML DG - granule cell layer of dentate gyrus; HATA - hippocampal amygdala transition area; HP - hippocampus.

Table S7-b: The effect of lifetime psychosis on hippocampal subfield volumes compared with controls (reference) (left hemisphere).

	**Without Lifetime psychosis**				**With lifetime psychosis**			
**Structure**	**Cohen's d**	**Lower CI**	**Upper CI**	**p-value**	**Cohen's d**	**Lower CI**	**Upper CI**	**p-value**
**Hippocampal tail**	−0.17	−0.23	−0.1	**0.0012**	−0.19	−0.25	−0.12	**2e−04**
**Subiculum**	−0.2	−0.26	−0.13	**2e−04**	−0.13	−0.2	−0.07	0.0098
**CA1**	−0.2	−0.26	−0.13	**1e−04**	−0.21	−0.28	−0.15	**2.3e−05**
**Hippocampal fissure**	0.03	−0.04	0.09	0.6152	0.05	−0.01	0.12	0.2845
**Presubiculum**	−0.17	−0.23	−0.1	**0.0015**	−0.21	−0.28	−0.15	**3.2e−05**
**Parasubiculum**	−0.04	−0.1	0.03	0.4769	−0.06	−0.12	0.01	0.2402
**Molecular layer HP**	−0.24	−0.31	−0.18	**3.0e−06**	−0.23	−0.29	−0.16	**6.3e−06**
**GC ML DG**	−0.24	−0.3	−0.17	**6.5e−06**	−0.24	−0.3	−0.17	**2.3e−06**
**CA2/3**	−0.15	−0.21	−0.08	0.0055	−0.12	−0.19	−0.06	0.0146
**CA4**	−0.21	−0.27	−0.14	**7.6e−05**	−0.2	−0.26	−0.13	**1e−04**
**Fimbria**	−0.1	−0.17	−0.04	0.0477	−0.12	−0.18	−0.05	0.0185
**HATA**	−0.19	−0.26	−0.13	**2e−04**	−0.24	−0.3	−0.17	**2.8e−06**
**Whole hippocampus**	−0.25	−0.32	−0.19	**1.5e−06**	−0.25	−0.32	−0.19	**6.9e−07**

Notes: Significant differences indicated in bold. *Abbreviations:* CI – confidence interval; CA – cornu ammonis; GC ML DG - granule cell layer of dentate gyrus; HATA - hippocampal amygdala transition area; HP - hippocampus.

Table S7-c: The effect of lifetime psychosis on hippocampal subfield volumes compared with controls (reference) (right hemisphere).

	**Without Lifetime psychosis**				**With lifetime psychosis**			
**Structure**	**Cohen's d**	**Lower CI**	**Upper CI**	**p-value**	**Cohen's d**	**Lower CI**	**Upper CI**	**p-value**
**Hippocampal tail**	−0.11	−0.17	−0.04	0.0397	−0.15	−0.21	−0.08	**0.0033**
**Subiculum**	−0.23	−0.3	−0.17	**7.5e−06**	−0.15	−0.22	−0.08	**0.003**
**CA1**	−0.22	−0.29	−0.16	**1.8e−05**	−0.22	−0.29	−0.16	**9.1e−06**
**Hippocampal fissure**	0.05	−0.01	0.12	0.3225	0.12	0.06	0.19	0.0138
**Presubiculum**	−0.17	−0.24	−0.1	0.0012	−0.2	−0.27	−0.14	**1e−04**
**Parasubiculum**	−0.05	−0.12	0.01	0.3274	−0.05	−0.11	0.02	0.3374
**Molecular layer HP**	−0.27	−0.34	−0.2	**2.8e−07**	−0.28	−0.34	−0.21	**4.3e−08**
**GC ML DG**	−0.22	−0.29	−0.16	**1.9e−05**	−0.27	−0.34	−0.21	**6.1e−08**
**CA2/3**	−0.14	−0.21	−0.08	0.0064	−0.16	−0.23	−0.1	**0.0014**
**CA4**	−0.21	−0.27	−0.14	**6.6e−05**	−0.26	−0.32	−0.19	**4.5e−07**
**Fimbria**	−0.09	−0.15	−0.02	0.1035	−0.1	−0.17	−0.04	0.0438
**HATA**	−0.18	−0.24	−0.11	**8e−04**	−0.15	−0.22	−0.09	**0.0028**
**Whole hippocampus**	−0.25	−0.31	−0.18	**2.1e−06**	−0.26	−0.33	−0.2	**2.6e−07**

Notes: Significant differences indicated in bold. *Abbreviations:* CI – confidence interval; CA – cornu ammonis; GC ML DG - granule cell layer of dentate gyrus; HATA - hippocampal amygdala transition area; HP - hippocampus.

Table S8: Effect of age at illness onset on hippocampal subfields volumes across bipolar disorder 1 and bipolar disorder 2 patients.

	Combined structures		Left hemisphere		Right hemisphere	
**Structure**	**Cohen's d**	**p-value**	**Cohen's d**	**p-value**	**Cohen's d**	**p-value**
**Hippocampal tail**	0.09	0.1095	0.08	0.1512	0.09	0.1135
**Subiculum**	−0.03	0.6597	−0.03	0.5703	−0.01	0.8262
**CA1**	0	0.9417	−0.02	0.7955	0	0.9393
**Hippocampal fissure**	0.06	0.3185	0.05	0.3436	0.05	0.3749
**Presubiculum**	−0.06	0.3269	−0.07	0.2618	−0.04	0.5108
**Parasubiculum**	0.01	0.8496	0.02	0.7179	0	0.9543
**Molecular layer HP**	−0.03	0.6342	−0.04	0.4528	−0.01	0.8587
**GC ML DG**	−0.03	0.622	−0.05	0.4328	−0.01	0.8626
**CA2/3**	0	0.9808	0	0.9447	0	0.9497
**CA4**	−0.02	0.6962	−0.04	0.4707	0	0.9641
**Fimbria**	0	0.9428	−0.01	0.8482	0.01	0.9249
**HATA**	−0.07	0.231	−0.03	0.6036	−0.1	0.0881
**Whole hippocampus**	0	0.9743	−0.01	0.8335	0.01	0.9034

Notes: The LME-models were adjusted for BD1/BD2 status. Combined structures imply left and right hemisphere combined. Significant differences indicated in bold. *Abbreviations:* CI – confidence interval; CA – cornu ammonis; GC ML DG - granule cell layer of dentate gyrus; HATA - hippocampal amygdala transition area; HP - hippocampus.

Table S9: Effect of duration of illness on hippocampal subfields volumes across bipolar disorder 1 and bipolar disorder 2 patients.

	**Combined structures**		**Left hemisphere**		**Right hemisphere**	
**Structure**	**Cohen's d**	**p-value**	**Cohen's d**	**p-value**	**Cohen's d**	**p-value**
**Hippocampal tail**	−0.09	0.1095	−0.08	0.1512	−0.09	0.1135
**Subiculum**	0.03	0.6597	0.03	0.5703	0.01	0.8262
**CA1**	0	0.9417	0.02	0.7955	0	0.9393
**Hippocampal fissure**	−0.06	0.3185	−0.05	0.3436	−0.05	0.3749
**Presubiculum**	0.06	0.3269	0.07	0.2618	0.04	0.5108
**Parasubiculum**	−0.01	0.8496	−0.02	0.7179	0	0.9543
**Molecular layer HP**	0.03	0.6342	0.04	0.4528	0.01	0.8587
**GC ML DG**	0.03	0.622	0.05	0.4328	0.01	0.8626
**CA2/3**	0	0.9808	0	0.9447	0	0.9497
**CA4**	0.02	0.6962	0.04	0.4707	0	0.9641
**Fimbria**	0	0.9428	0.01	0.8482	−0.01	0.9249
**HATA**	0.07	0.231	0.03	0.6036	0.1	0.0881
**Whole hippocampus**	0	0.9743	0.01	0.8335	−0.01	0.9034

Notes: The LME-models were adjusted for BD1/BD2 status. Combined structures imply left and right hemisphere combined. Significant differences indicated in bold. *Abbreviations:* CI – confidence interval; CA – cornu ammonis; GC ML DG - granule cell layer of dentate gyrus; HATA - hippocampal amygdala transition area; HP - hippocampus.

Table S10: Effect of total PANSS positive scores on hippocampal subfield volumes across bipolar disorder 1 and bipolar disorder 2 patients.

	**Combined structures**		**Left hemisphere**		**Right hemisphere**	
**Structure**	**Cohen's d**	**p-value**	**Cohen's d**	**p-value**	**Cohen's d**	**p-value**
**Hippocampal tail**	−0.09	0.333	−0.01	0.8831	−0.15	0.1045
**Subiculum**	0.02	0.7938	0.02	0.8211	0.03	0.7502
**CA1**	0.05	0.5603	0.08	0.3877	0.02	0.816
**Hippocampal fissure**	0.12	0.1977	0.1	0.2987	0.11	0.2253
**Presubiculum**	0.03	0.7226	0.01	0.8997	0.05	0.5894
**Parasubiculum**	0.1	0.2524	0.06	0.492	0.12	0.1751
**Molecular layer HP**	0.04	0.6475	0.07	0.4681	0.01	0.9066
**GC ML DG**	0.08	0.4025	0.07	0.4334	0.07	0.4633
**CA2/3**	0.01	0.8835	0.03	0.739	−0.01	0.9516
**CA4**	0.06	0.4859	0.06	0.5478	0.06	0.5168
**Fimbria**	0.11	0.2271	0.06	0.4993	0.13	0.1458
**HATA**	−0.07	0.4576	−0.04	0.6897	−0.08	0.3673
**Whole hippocampus**	0.02	0.8047	0.04	0.674	0.01	0.9489

Notes: The LME-models were adjusted for BD1/BD2 status. Combined structures imply left and right hemisphere combined. Significant differences indicated in bold. *Abbreviations:* CI – confidence interval; CA – cornu ammonis; GC ML DG - granule cell layer of dentate gyrus; HATA - hippocampal amygdala transition area; HP - hippocampus.

Table S11: Effect of total PANSS negative score on hippocampal subfield volumes across bipolar disorder 1 and bipolar disorder 2 patients.

	**Combined structures**		**Left hemisphere**		**Right hemisphere**	
**Structure**	**Cohen's d**	**p-value**	**Cohen's d**	**p-value**	**Cohen's d**	**p-value**
**Hippocampal tail**	−0.04	0.6619	−0.03	0.7373	−0.04	0.6618
**Subiculum**	−0.2	0.03	−0.19	0.0439	−0.19	0.0415
**CA1**	−0.2	0.0312	−0.15	0.1143	−0.22	0.0188
**Hippocampal fissure**	−0.14	0.1218	0.01	0.8952	−0.27	0.003
**Presubiculum**	−0.24	0.0094	−0.24	0.0104	−0.2	0.0306
**Parasubiculum**	−0.01	0.8962	−0.05	0.6079	0.04	0.6735
**Molecular layer HP**	−0.19	0.0356	−0.13	0.1717	−0.24	0.0105
**GC ML DG**	−0.18	0.0498	−0.13	0.1495	−0.2	0.0322
**CA2/3**	−0.09	0.3249	−0.01	0.9014	−0.15	0.1099
**CA4**	−0.17	0.0631	−0.11	0.2286	−0.2	0.029
**Fimbria**	0	0.9955	−0.04	0.6574	0.04	0.6834
**HATA**	−0.09	0.3154	−0.14	0.1307	−0.02	0.8326
**Whole hippocampus**	−0.18	0.053	−0.14	0.1211	−0.2	0.0321

Notes: The LME-models were adjusted for BD1/BD2 status. Combined structures imply left and right hemisphere combined. Significant differences indicated in bold. *Abbreviations:* CI – confidence interval; CA – cornu ammonis; GC ML DG - granule cell layer of dentate gyrus; HATA - hippocampal amygdala transition area; HP - hippocampus.

Table S12: Effect of lithium medication on hippocampal subfield volumes among bipolar disorder 1 patients (reference lithium non-users).

	**Combined structures**				**Left structures**				**Right structures**			
**Structure**	**Cohen's d**	**Lower CI**	**Upper CI**	**p-value**	**Cohen's d**	**Lower CI**	**Upper CI**	**p-value**	**Cohen's d**	**Lower CI**	**Upper CI**	**p-value**
**Hippocampal tail**	0.19	0.05	0.33	0.0114	0.2	0.06	0.34	0.0082	0.15	0.01	0.29	0.0395
**Subiculum**	0.1	−0.04	0.24	0.1672	0.06	−0.08	0.2	0.3971	0.13	−0.01	0.28	0.0692
**CA1**	0.2	0.06	0.34	0.006	0.21	0.07	0.35	0.0052	0.18	0.04	0.32	0.017
**Hippocampal fissure**	−0.24	−0.38	−0.1	**0.0011**	−0.21	−0.35	−0.07	0.0043	−0.22	−0.36	−0.08	**0.0029**
**Presubiculum**	0.17	0.03	0.31	0.019	0.13	−0.01	0.27	0.0872	0.19	0.05	0.33	0.0106
**Parasubiculum**	0.04	−0.1	0.18	0.5886	0.01	−0.13	0.15	0.8802	0.07	−0.07	0.21	0.3689
**Molecular layer HP**	0.23	0.09	0.37	**0.002**	0.22	0.08	0.36	**0.0036**	0.22	0.07	0.36	**0.0037**
**GC ML DG**	0.22	0.08	0.36	**0.0034**	0.21	0.07	0.35	0.0039	0.19	0.05	0.33	0.0112
**CA2/3**	0.15	0.01	0.29	0.0383	0.18	0.04	0.32	0.0136	0.1	−0.04	0.24	0.1806
**CA4**	0.2	0.06	0.34	0.0079	0.19	0.05	0.33	0.01	0.17	0.03	0.31	0.0209
**Fimbria**	0.04	−0.1	0.18	0.578	0.03	−0.11	0.17	0.7311	0.05	−0.09	0.19	0.534
**HATA**	0.18	0.04	0.32	0.0131	0.22	0.08	0.36	**0.0035**	0.11	−0.03	0.25	0.1263
**Whole hippocampus**	0.22	0.08	0.36	**0.0028**	0.22	0.07	0.36	**0.0038**	0.21	0.07	0.35	0.0045

Notes: Combined structures imply left and right hemisphere combined. Significant differences indicated in bold. *Abbreviations:* CI – confidence interval; CA – cornu ammonis; GC ML DG - granule cell layer of dentate gyrus; HATA - hippocampal amygdala transition area; HP - hippocampus.

Table S13-a: Effect of lithium medication on hippocampal subfield volumes among bipolar disorder 1 patients (lithium users and non-users) with controls as reference (both hemispheres combined).

	Lithium users				Lithium non-users			
**Structure**	**Cohen's d**	**Lower CI**	**Upper CI**	**p-value**	**Cohen's d**	**Lower CI**	**Upper CI**	**p-value**
**Hippocampal tail**	−0.08	−0.14	−0.01	0.162	−0.22	−0.29	−0.16	**3.7e−06**
**Subiculum**	−0.14	−0.21	−0.07	0.0116	−0.2	−0.27	−0.14	**2.0e−05**
**CA1**	−0.09	−0.15	−0.02	0.1144	−0.27	−0.33	−0.2	**1.9e−08**
**Hippocampal fissure**	−0.09	−0.16	−0.02	0.1058	0.15	0.08	0.21	**0.0023**
**Presubiculum**	−0.15	−0.22	−0.08	0.0065	−0.28	−0.35	−0.22	**3.9e−09**
**Parasubiculum**	−0.07	−0.14	−0.01	0.1773	−0.08	−0.15	−0.02	0.0788
**Molecular layer HP**	−0.12	−0.19	−0.05	0.0293	−0.32	−0.39	−0.26	**2.1e−11**
**GC ML DG**	−0.1	−0.17	−0.03	0.0689	−0.3	−0.36	−0.23	**4.6e−10**
**CA2/3**	−0.04	−0.1	0.03	0.5003	−0.18	−0.24	−0.11	**2e−04**
**CA4**	−0.1	−0.16	−0.03	0.0793	−0.27	−0.34	−0.21	**1.3e−08**
**Fimbria**	−0.06	−0.13	0.01	0.2776	−0.09	−0.15	−0.02	0.0731
**HATA**	−0.1	−0.17	−0.04	0.059	−0.24	−0.3	−0.17	**7.0e−07**
**Whole hippocampus**	−0.13	−0.2	−0.06	0.0188	−0.31	−0.38	−0.25	**9.4e−11**

Notes: Significant differences indicated in bold. *Abbreviations:* CI – confidence interval; CA – cornu ammonis; GC ML DG - granule cell layer of dentate gyrus; HATA - hippocampal amygdala transition area; HP - hippocampus.

Table S13-b: Effect of lithium medication on hippocampal subfield volumes among bipolar disorder 1 patients (lithium users and non-users) with controls as reference (left hemisphere).

	**Lithium users**				**Lithium non-users**			
**Structure**	**Cohen's d**	**Lower CI**	**Upper CI**	**p-value**	**Cohen's d**	**Lower CI**	**Upper CI**	**p-value**
**Hippocampal tail**	−0.08	−0.15	−0.02	0.1349	−0.24	−0.3	−0.17	**9.1e−07**
**Subiculum**	−0.16	−0.23	−0.09	0.0039	−0.19	−0.26	−0.13	**5.0e−05**
**CA1**	−0.09	−0.16	−0.03	0.0971	−0.26	−0.32	−0.2	**5.9e−08**
**Hippocampal fissure**	−0.13	−0.19	−0.06	0.0228	0.07	0.01	0.13	0.1422
**Presubiculum**	−0.17	−0.24	−0.11	**0.0018**	−0.26	−0.33	−0.2	**3.4e−08**
**Parasubiculum**	−0.1	−0.16	−0.03	0.0832	−0.07	−0.14	−0.01	0.1381
**Molecular layer HP**	−0.13	−0.19	−0.06	0.0232	−0.3	−0.37	−0.24	**2.1e−10**
**GC ML DG**	−0.09	−0.16	−0.03	0.0909	−0.27	−0.33	−0.2	**1.9e−08**
**CA2/3**	−0.01	−0.08	0.05	0.8416	−0.16	−0.22	−0.09	**0.0011**
**CA4**	−0.09	−0.16	−0.02	0.1058	−0.24	−0.31	−0.18	**4.6e−07**
**Fimbria**	−0.07	−0.13	0	0.2393	−0.08	−0.14	−0.01	0.1122
**HATA**	−0.1	−0.16	−0.03	0.0771	−0.24	−0.31	−0.18	**4.1e−07**
**Whole hippocampus**	−0.14	−0.2	−0.07	0.0137	−0.3	−0.37	−0.24	**3.2e−10**

Notes: Significant differences indicated in bold. *Abbreviations:* CI – confidence interval; CA – cornu ammonis; GC ML DG - granule cell layer of dentate gyrus; HATA - hippocampal amygdala transition area; HP - hippocampus.

Table S13-c: Effect of lithium medication on hippocampal subfield volumes among bipolar disorder 1 patients (lithium users and non-users) with controls as reference (right hemisphere).

	Lithium users				Lithium non-users			
**Structure**	**Cohen's d**	**Lower CI**	**Upper CI**	**p-value**	**Cohen's d**	**Lower CI**	**Upper CI**	**p-value**
**Hippocampal tail**	−0.06	−0.13	0	0.2676	−0.18	−0.25	−0.12	**2e−04**
**Subiculum**	−0.1	−0.17	−0.04	0.0681	−0.2	−0.26	−0.13	**4.7e−05**
**CA1**	−0.07	−0.14	0	0.2119	−0.24	−0.31	−0.18	**5.1e−07**
**Hippocampal fissure**	−0.04	−0.1	0.03	0.4916	0.19	0.12	0.25	**8.1e−05**
**Presubiculum**	−0.1	−0.17	−0.03	0.0712	−0.25	−0.32	−0.19	**1.5e−07**
**Parasubiculum**	−0.04	−0.1	0.03	0.523	−0.08	−0.15	−0.02	0.0859
**Molecular layer HP**	−0.1	−0.17	−0.03	0.071	−0.3	−0.36	−0.23	**4.5e−10**
**GC ML DG**	−0.09	−0.16	−0.02	0.1053	−0.28	−0.34	−0.21	**5.5e−09**
**CA2/3**	−0.05	−0.12	0.01	0.3437	−0.16	−0.22	−0.1	**9e−04**
**CA4**	−0.09	−0.15	−0.02	0.1235	−0.25	−0.32	−0.19	**1.1e−07**
**Fimbria**	−0.04	−0.11	0.02	0.4544	−0.08	−0.14	−0.01	0.1124
**HATA**	−0.09	−0.15	−0.02	0.1209	−0.18	−0.24	−0.11	**2e−04**
**Whole hippocampus**	−0.11	−0.18	−0.05	0.0448	−0.29	−0.36	−0.23	**1.1e−09**

Notes: Significant differences indicated in bold. *Abbreviations:* CI – confidence interval; CA – cornu ammonis; GC ML DG - granule cell layer of dentate gyrus; HATA - hippocampal amygdala transition area; HP - hippocampus.

Table S14: Effects of antipsychotic medication on hippocampal subfield volumes among bipolar disorder 1 patients (reference antipsychotic non-users).

	Combined structured				Left structures				Right structures			
**Structure**	**Cohen's d**	**Lower CI**	**Upper CI**	**p-value**	**Cohen's d**	**Lower CI**	**Upper CI**	**p-value**	**Cohen's d**	**Lower CI**	**Upper CI**	**p-value**
**Hippocampal tail**	−0.09	−0.22	0.05	0.2074	−0.12	−0.25	0.02	0.0898	−0.04	−0.18	0.09	0.5252
**Subiculum**	−0.09	−0.22	0.04	0.1967	−0.06	−0.19	0.07	0.3842	−0.11	−0.25	0.02	0.1108
**CA1**	−0.14	−0.28	−0.01	0.0425	−0.12	−0.26	0.01	0.082	−0.14	−0.28	−0.01	0.0402
**Hippocampal fissure**	0.14	0.01	0.27	0.0457	0.1	−0.04	0.23	0.1666	0.15	0.02	0.29	0.0274
**Presubiculum**	−0.24	−0.37	−0.1	**7e−04**	−0.17	−0.31	−0.04	0.014	−0.26	−0.39	−0.13	**2e−04**
**Parasubiculum**	−0.1	−0.23	0.03	0.1486	−0.09	−0.23	0.04	0.189	−0.09	−0.22	0.05	0.2217
**Molecular layer HP**	−0.16	−0.3	−0.03	0.0189	−0.14	−0.28	−0.01	0.0399	−0.17	−0.3	−0.03	0.0181
**GC ML DG**	−0.13	−0.26	0.01	0.069	−0.12	−0.25	0.01	0.0861	−0.12	−0.25	0.02	0.097
**CA2/3**	−0.02	−0.15	0.12	0.7866	−0.04	−0.17	0.1	0.5759	0	−0.13	0.14	0.9822
**CA4**	−0.1	−0.23	0.04	0.168	−0.09	−0.22	0.04	0.2007	−0.09	−0.22	0.05	0.2043
**Fimbria**	−0.14	−0.28	−0.01	0.0392	−0.09	−0.22	0.05	0.2148	−0.16	−0.3	−0.03	0.0204
**HATA**	−0.14	−0.28	−0.01	0.043	−0.13	−0.26	0.01	0.0672	−0.13	−0.26	0.01	0.0668
**Whole hippocampus**	−0.16	−0.29	−0.03	0.0218	−0.16	−0.3	−0.03	0.0197	−0.15	−0.28	−0.02	0.0321

Notes: Combined structures imply left and right hemisphere combined. Significant differences indicated in bold. *Abbreviations:* CI – confidence interval; CA – cornu ammonis; GC ML DG - granule cell layer of dentate gyrus; HATA - hippocampal amygdala transition area; HP - hippocampus.

Table S15-a: Effect of antipsychotic medication on hippocampal subfield volumes among bipolar disorder 1 patients (antipsychotic users and non-users) with controls as reference (both hemispheres combined).

	**Antipsychotic users**				**Antipsychotic non-users**			
**Structure**	**Cohen's d**	**Lower CI**	**Upper CI**	**p-value**	**Cohen's d**	**Lower CI**	**Upper CI**	**p-value**
**Hippocampal tail**	−0.15	−0.22	−0.09	**0.0014**	−0.08	−0.15	−0.02	0.1086
**Subiculum**	−0.2	−0.26	−0.13	**3.4e−05**	−0.13	−0.19	−0.06	0.0126
**CA1**	−0.23	−0.29	−0.16	**1.4e−06**	−0.11	−0.17	−0.04	0.0377
**Hippocampal fissure**	0.12	0.06	0.19	0.0092	−0.02	−0.09	0.04	0.6804
**Presubiculum**	−0.3	−0.37	−0.24	**2.0e−10**	−0.1	−0.16	−0.03	0.0582
**Parasubiculum**	−0.14	−0.2	−0.07	**0.0037**	−0.06	−0.12	0.01	0.2721
**Molecular layer HP**	−0.28	−0.35	−0.22	**1.7e−09**	−0.14	−0.2	−0.07	0.0067
**GC ML DG**	−0.27	−0.34	−0.21	**6.7e−09**	−0.16	−0.22	−0.09	**0.0022**
**CA2/3**	−0.11	−0.18	−0.05	0.0159	−0.08	−0.15	−0.02	0.1064
**CA4**	−0.24	−0.3	−0.18	**3.6e−07**	−0.15	−0.22	−0.08	**0.0032**
**Fimbria**	−0.13	−0.2	−0.07	0.0043	−0.01	−0.08	0.05	0.7707
**HATA**	−0.23	−0.29	−0.16	**1.2e−06**	−0.11	−0.17	−0.04	0.0336
**Whole hippocampus**	−0.27	−0.34	−0.21	**6.4e−09**	−0.14	−0.2	−0.07	0.008

Notes: Significant differences indicated in bold. *Abbreviations:* CI – confidence interval; CA – cornu ammonis; GC ML DG - granule cell layer of dentate gyrus; HATA - hippocampal amygdala transition area; HP - hippocampus.

Table S15-b: Effect of antipsychotic medication on hippocampal subfield volumes among bipolar disorder 1 patients (antipsychotic users and non-users) with controls as reference (left hemisphere).

	Antipsychotic users				Antipsychotic non-users			
**Structure**	**Cohen's d**	**Lower CI**	**Upper CI**	**p-value**	**Cohen's d**	**Lower CI**	**Upper CI**	**p-value**
**Hippocampal tail**	−0.17	−0.24	−0.11	**2e−04**	−0.08	−0.14	−0.01	0.1293
**Subiculum**	−0.17	−0.24	−0.11	**2e−04**	−0.13	−0.19	−0.06	0.0119
**CA1**	−0.21	−0.27	−0.14	**1.1e−05**	−0.11	−0.17	−0.04	0.0331
**Hippocampal fissure**	0.05	−0.01	0.12	0.2566	−0.05	−0.11	0.02	0.3633
**Presubiculum**	−0.27	−0.34	−0.21	**9.1e−09**	−0.12	−0.19	−0.06	0.0157
**Parasubiculum**	−0.14	−0.2	−0.07	0.004	−0.06	−0.13	0	0.214
**Molecular layer HP**	−0.26	−0.32	−0.19	**4.8e−08**	−0.14	−0.2	−0.07	0.007
**GC ML DG**	−0.24	−0.31	−0.18	**2.9e−07**	−0.14	−0.21	−0.08	0.0044
**CA2/3**	−0.11	−0.17	−0.04	0.0257	−0.07	−0.13	0	0.1806
**CA4**	−0.21	−0.28	−0.15	**7.0e−06**	−0.14	−0.21	−0.08	0.005
**Fimbria**	−0.12	−0.18	−0.05	0.0117	−0.04	−0.11	0.02	0.417
**HATA**	−0.22	−0.29	−0.16	**2.3e−06**	−0.13	−0.19	−0.06	0.0117
**Whole hippocampus**	−0.27	−0.33	−0.2	**1.8e−08**	−0.13	−0.2	−0.07	0.0101

Notes: Significant differences indicated in bold. *Abbreviations:* CI – confidence interval; CA – cornu ammonis; GC ML DG - granule cell layer of dentate gyrus; HATA - hippocampal amygdala transition area; HP - hippocampus.

Table S15-c: Effect of antipsychotic medication on hippocampal subfield volumes among bipolar disorder 1 patients (antipsychotic users and non-users) with controls as reference (right hemisphere).

	**Antipsychotic users**				**Antipsychotic non-users**			
**Structure**	**Cohen's d**	**Lower CI**	**Upper CI**	**p-value**	**Cohen's d**	**Lower CI**	**Upper CI**	**p-value**
**Hippocampal tail**	−0.11	−0.17	−0.04	0.0213	−0.08	−0.14	−0.01	0.1276
**Subiculum**	−0.2	−0.26	−0.13	**2.3e−05**	−0.11	−0.18	−0.05	0.0287
**CA1**	−0.22	−0.28	−0.15	**4.9e−06**	−0.09	−0.15	−0.02	0.0896
**Hippocampal fissure**	0.16	0.1	0.23	**6e−04**	0	−0.06	0.07	0.9311
**Presubiculum**	−0.28	−0.34	−0.22	**3.0e−09**	−0.05	−0.12	0.01	0.3119
**Parasubiculum**	−0.11	−0.17	−0.04	0.0203	−0.04	−0.1	0.03	0.4559
**Molecular layer HP**	−0.28	−0.34	−0.21	**4.6e−09**	−0.12	−0.19	−0.06	0.0171
**GC ML DG**	−0.26	−0.33	−0.2	**3.1e−08**	−0.14	−0.21	−0.08	0.0057
**CA2/3**	−0.1	−0.16	−0.04	0.0347	−0.08	−0.14	−0.01	0.1255
**CA4**	−0.23	−0.29	−0.16	**1.8e−06**	−0.13	−0.19	−0.06	0.0114
**Fimbria**	−0.11	−0.18	−0.05	0.0147	0.02	−0.05	0.08	0.7367
**HATA**	−0.18	−0.25	−0.12	**1e−04**	−0.06	−0.13	0	0.2335
**Whole hippocampus**	−0.26	−0.32	−0.2	**3.7e−08**	−0.13	−0.19	−0.06	0.0136

Notes: Significant differences indicated in bold. *Abbreviations:* CI – confidence interval; CA – cornu ammonis; GC ML DG - granule cell layer of dentate gyrus; HATA - hippocampal amygdala transition area; HP - hippocampus.

Table S16: Effect of antiepileptic medication on hippocampal subfield volumes among bipolar disorder 1 patients (reference antiepileptic non-users).

	Combined structures				Left structures				Right structures			
**Structure**	**Cohen's d**	**Lower CI**	**Upper CI**	**p-value**	**Cohen's d**	**Lower CI**	**Upper CI**	**p-value**	**Cohen's d**	**Lower CI**	**Upper CI**	**p-value**
**Hippocampal tail**	−0.25	−0.42	−0.09	**0.0032**	−0.22	−0.38	−0.05	0.0112	−0.25	−0.42	−0.09	**0.0035**
**Subiculum**	−0.16	−0.33	0	0.0604	−0.19	−0.35	−0.02	0.0308	−0.11	−0.28	0.05	0.1839
**CA1**	−0.04	−0.21	0.12	0.6164	−0.05	−0.21	0.12	0.6001	−0.04	−0.2	0.13	0.6767
**Hippocampal fissure**	0.12	−0.04	0.29	0.1587	0.04	−0.12	0.21	0.6236	0.18	0.01	0.34	0.0382
**Presubiculum**	−0.15	−0.31	0.02	0.09	−0.13	−0.3	0.03	0.1205	−0.14	−0.3	0.03	0.1105
**Parasubiculum**	0.04	−0.13	0.2	0.6777	0.07	−0.09	0.24	0.4105	−0.03	−0.19	0.14	0.7443
**Molecular layer HP**	−0.11	−0.28	0.05	0.1974	−0.12	−0.29	0.05	0.1629	−0.09	−0.25	0.08	0.3098
**GC ML DG**	−0.04	−0.21	0.12	0.6219	−0.07	−0.23	0.1	0.4283	−0.01	−0.17	0.16	0.9163
**CA2/3**	0.06	−0.1	0.23	0.4796	0.02	−0.14	0.19	0.7792	0.09	−0.08	0.25	0.3158
**CA4**	−0.02	−0.19	0.14	0.7851	−0.05	−0.21	0.12	0.5828	0.01	−0.16	0.17	0.9453
**Fimbria**	−0.06	−0.22	0.11	0.4951	−0.01	−0.17	0.16	0.9106	−0.09	−0.26	0.07	0.2709
**HATA**	−0.05	−0.22	0.11	0.5345	−0.09	−0.26	0.07	0.2775	0	−0.16	0.17	0.9863
**Whole hippocampus**	−0.12	−0.29	0.04	0.1552	−0.12	−0.29	0.05	0.1639	−0.11	−0.28	0.05	0.1974

Notes: Combined structures imply left and right hemisphere combined. Significant differences indicated in bold. *Abbreviations:* CI – confidence interval; CA – cornu ammonis; GC ML DG - granule cell layer of dentate gyrus; HATA - hippocampal amygdala transition area; HP - hippocampus.

Table S17-a: Effect of antiepileptic medication on hippocampal subfield volumes among bipolar disorder 1 patients (antiepileptic users and non-users) with controls as reference (both hemispheres combined).

	**Antiepileptic users**				**Antiepileptic non-users**			
**Structure**	**Cohen's d**	**Lower CI**	**Upper CI**	**p-value**	**Cohen's d**	**Lower CI**	**Upper CI**	**p-value**
**Hippocampal tail**	−0.31	−0.37	−0.24	**9.9e−07**	−0.11	−0.17	−0.04	0.0676
**Subiculum**	−0.26	−0.32	−0.19	**3.8e−05**	−0.14	−0.21	−0.08	0.0145
**CA1**	−0.26	−0.33	−0.2	**2.4e−05**	−0.23	−0.29	−0.16	**9.2e−05**
**Hippocampal fissure**	0.16	0.09	0.22	0.0124	0.01	−0.06	0.07	0.9216
**Presubiculum**	−0.3	−0.37	−0.23	**1.5e−06**	−0.2	−0.26	−0.13	**7e−04**
**Parasubiculum**	−0.08	−0.15	−0.01	0.2067	−0.11	−0.18	−0.04	0.0568
**Molecular layer HP**	−0.34	−0.41	−0.27	**5.5e−08**	−0.24	−0.31	−0.18	**2.8e−05**
**GC ML DG**	−0.31	−0.37	−0.24	**9.7e−07**	−0.26	−0.32	−0.19	**1.0e−05**
**CA2/3**	−0.15	−0.22	−0.09	0.0149	−0.18	−0.24	−0.11	**0.0022**
**CA4**	−0.27	−0.34	−0.21	**1.2e−05**	−0.24	−0.3	−0.17	**3.8e−05**
**Fimbria**	−0.12	−0.19	−0.06	0.0476	−0.06	−0.12	0.01	0.3202
**HATA**	−0.24	−0.31	−0.18	**1e−04**	−0.16	−0.22	−0.09	0.0069
**Whole hippocampus**	−0.34	−0.4	−0.27	**7.8e−08**	−0.23	−0.3	−0.16	**6.6e−05**

Notes: Significant differences indicated in bold. *Abbreviations:* CI – confidence interval; CA – cornu ammonis; GC ML DG - granule cell layer of dentate gyrus; HATA - hippocampal amygdala transition area; HP - hippocampus.

Table S17-b: Effect of antiepileptic medication on hippocampal subfield volumes among bipolar disorder 1 patients (antiepileptic users and non-users) with controls as reference (left hemisphere).

	**Antiepileptic users**				**Antiepileptic non-users**			
**Structure**	**Cohen's d**	**Lower CI**	**Upper CI**	**p-value**	**Cohen's d**	**Lower CI**	**Upper CI**	**p-value**
**Hippocampal tail**	−0.3	−0.37	−0.23	**1.5e−06**	−0.12	−0.19	−0.06	0.0317
**Subiculum**	−0.26	−0.33	−0.2	**2.4e−05**	−0.13	−0.19	−0.06	0.0265
**CA1**	−0.24	−0.31	−0.17	**1e−04**	−0.2	−0.27	−0.13	**5e−04**
**Hippocampal fissure**	0.05	−0.01	0.12	0.3941	−0.03	−0.1	0.03	0.5794
**Presubiculum**	−0.29	−0.35	−0.22	**5.0e−06**	−0.18	−0.25	−0.11	**0.0019**
**Parasubiculum**	−0.05	−0.11	0.02	0.4486	−0.1	−0.17	−0.04	0.0771
**Molecular layer HP**	−0.31	−0.38	−0.25	**5.9e−07**	−0.21	−0.28	−0.14	**3e−04**
**GC ML DG**	−0.27	−0.34	−0.21	**1.2e−05**	−0.21	−0.27	−0.14	**4e−04**
**CA2/3**	−0.13	−0.19	−0.06	0.0451	−0.12	−0.19	−0.06	0.034
**CA4**	−0.24	−0.31	−0.17	**1e−04**	−0.19	−0.26	−0.12	**0.001**
**Fimbria**	−0.1	−0.17	−0.03	0.1098	−0.07	−0.13	0	0.245
**HATA**	−0.27	−0.33	−0.2	**2.0e−05**	−0.14	−0.21	−0.08	0.0143
**Whole hippocampus**	−0.32	−0.38	−0.25	**4.8e−07**	−0.21	−0.28	−0.15	**2e−04**

Notes: Significant differences indicated in bold. *Abbreviations:* CI – confidence interval; CA – cornu ammonis; GC ML DG - granule cell layer of dentate gyrus; HATA - hippocampal amygdala transition area; HP - hippocampus.

Table S17-c: Effect of antiepileptic medication on hippocampal subfield volumes among bipolar disorder 1 patients (antiepileptic users and non-users) with controls as reference (right hemisphere).

	**Antiepileptic users**				**Antiepileptic non-users**			
**Structure**	**Cohen's d**	**Lower CI**	**Upper CI**	**p-value**	**Cohen's d**	**Lower CI**	**Upper CI**	**p-value**
**Hippocampal tail**	−0.27	−0.34	−0.2	**1.6e−05**	−0.07	−0.14	−0.01	0.1949
**Subiculum**	−0.23	−0.29	−0.16	**3e−04**	−0.14	−0.21	−0.07	0.0149
**CA1**	−0.25	−0.32	−0.18	**5.9e−05**	−0.22	−0.29	−0.15	**2e−04**
**Hippocampal fissure**	0.22	0.16	0.29	**4e−04**	0.04	−0.03	0.1	0.5048
**Presubiculum**	−0.27	−0.33	−0.2	**1.9e−05**	−0.18	−0.25	−0.11	**0.0019**
**Parasubiculum**	−0.1	−0.17	−0.04	0.0932	−0.1	−0.16	−0.03	0.0883
**Molecular layer HP**	−0.33	−0.39	−0.26	**1.7e−07**	−0.25	−0.31	−0.18	**2.2e−05**
**GC ML DG**	−0.29	−0.36	−0.22	**3.5e−06**	−0.26	−0.33	−0.2	**5.1e−06**
**CA2/3**	−0.15	−0.21	−0.08	0.0177	−0.19	−0.26	−0.13	**7e−04**
**CA4**	−0.26	−0.33	−0.19	**3.3e−05**	−0.24	−0.31	−0.18	**2.3e−05**
**Fimbria**	−0.12	−0.19	−0.05	0.0559	−0.03	−0.1	0.03	0.5592
**HATA**	−0.16	−0.22	−0.09	0.0115	−0.13	−0.2	−0.07	0.0197
**Whole hippocampus**	−0.32	−0.39	−0.26	**2.2e−07**	−0.23	−0.3	−0.16	**6.9e−05**

Notes: Significant differences indicated in bold. *Abbreviations:* CI – confidence interval; CA – cornu ammonis; GC ML DG - granule cell layer of dentate gyrus; HATA - hippocampal amygdala transition area; HP - hippocampus.

Table S18: Effect of antidepressant medication on hippocampal subfield volumes among bipolar disorder 1 patients (reference antidepressant non-users).

	**Combined structures**				**Left structures**				**Right structures**			
**Structure**	**Cohen's d**	**Lower CI**	**Upper CI**	**p-value**	**Cohen's d**	**Lower CI**	**Upper CI**	**p-value**	**Cohen's d**	**Lower CI**	**Upper CI**	**p-value**
**Hippocampal tail**	−0.02	−0.19	0.14	0.8227	−0.02	−0.18	0.15	0.8528	−0.02	−0.19	0.14	0.8025
**Subiculum**	−0.04	−0.21	0.12	0.6667	−0.03	−0.19	0.14	0.7637	−0.05	−0.21	0.12	0.6351
**CA1**	0	−0.16	0.17	0.9687	−0.01	−0.18	0.15	0.9131	0.02	−0.15	0.18	0.8587
**Hippocampal fissure**	−0.13	−0.29	0.04	0.1901	−0.11	−0.28	0.05	0.2395	−0.12	−0.29	0.05	0.2124
**Presubiculum**	0.01	−0.15	0.18	0.8795	−0.01	−0.18	0.15	0.8849	0.05	−0.12	0.21	0.6362
**Parasubiculum**	−0.08	−0.24	0.09	0.4338	−0.12	−0.28	0.05	0.2163	0	−0.16	0.17	0.9596
**Molecular layer HP**	0	−0.17	0.16	0.9822	0	−0.17	0.16	0.9613	0	−0.16	0.17	0.9916
**GC ML DG**	0.05	−0.11	0.22	0.5994	0.1	−0.06	0.27	0.2759	−0.01	−0.18	0.15	0.9067
**CA2/3**	−0.05	−0.22	0.11	0.5717	−0.01	−0.17	0.16	0.9328	−0.09	−0.25	0.08	0.3627
**CA4**	0.03	−0.13	0.2	0.7251	0.1	−0.07	0.26	0.3096	−0.03	−0.2	0.13	0.7199
**Fimbria**	0.11	−0.06	0.27	0.2569	0.09	−0.08	0.25	0.3624	0.11	−0.06	0.27	0.2642
**HATA**	0.04	−0.13	0.2	0.6995	0	−0.17	0.16	0.9934	0.07	−0.09	0.24	0.4501
**Whole hippocampus**	−0.01	−0.17	0.16	0.9377	−0.01	−0.17	0.16	0.9292	−0.01	−0.17	0.16	0.9406

Notes: Combined structures imply left and right hemisphere combined. Significant differences indicated in bold. *Abbreviations:* CI – confidence interval; CA – cornu ammonis; GC ML DG - granule cell layer of dentate gyrus; HATA - hippocampal amygdala transition area; HP - hippocampus.

Table S19-a: Effect of antidepressant medication among bipolar disorder 1 patients (antidepressant users and non-users) with controls as reference (both hemispheres combined).

	**Antidepressant users**				**Antidepressant non-users**			
**Structure**	**Cohen's d**	**Lower CI**	**Upper CI**	**p-value**	**Cohen's d**	**Lower CI**	**Upper CI**	**p-value**
**Hippocampal tail**	−0.21	−0.28	−0.14	0.0077	−0.18	−0.24	−0.11	**7e−04**
**Subiculum**	−0.23	−0.29	−0.16	**0.0036**	−0.17	−0.23	−0.1	**0.0013**
**CA1**	−0.26	−0.33	−0.19	**9e−04**	−0.23	−0.29	−0.16	**1.1e−05**
**Hippocampal fissure**	−0.03	−0.1	0.04	0.6811	0.1	0.04	0.17	0.0438
**Presubiculum**	−0.23	−0.3	−0.16	**0.0034**	−0.24	−0.3	−0.17	**5.0e−06**
**Parasubiculum**	−0.14	−0.21	−0.08	0.067	−0.08	−0.14	−0.01	0.1379
**Molecular layer HP**	−0.3	−0.37	−0.24	**1e−04**	−0.26	−0.33	−0.2	**3.1e−07**
**GC ML DG**	−0.26	−0.33	−0.2	**7e−04**	−0.27	−0.34	−0.21	**1.3e−07**
**CA2/3**	−0.21	−0.28	−0.15	0.0063	−0.14	−0.21	−0.08	0.0055
**CA4**	−0.25	−0.32	−0.18	**0.0014**	−0.25	−0.31	−0.18	**1.9e−06**
**Fimbria**	0	−0.07	0.06	0.967	−0.11	−0.18	−0.05	0.0268
**HATA**	−0.18	−0.25	−0.11	0.0224	−0.19	−0.26	−0.12	**2e−04**
**Whole hippocampus**	−0.29	−0.36	−0.22	**2e−04**	−0.26	−0.32	−0.19	**6.4e−07**

Notes: Significant differences indicated in bold. *Abbreviations:* CI – confidence interval; CA – cornu ammonis; GC ML DG - granule cell layer of dentate gyrus; HATA - hippocampal amygdala transition area; HP - hippocampus.

Table S19-b: Effect of antidepressant medication on hippocampal subfield volumes among bipolar disorder 1 patients (antidepressant users and non-users) with controls as reference (left hemisphere).

	Antidepressant users				Antidepressant non-users			
**Structure**	**Cohen's d**	**Lower CI**	**Upper CI**	**p-value**	**Cohen's d**	**Lower CI**	**Upper CI**	**p-value**
**Hippocampal tail**	−0.22	−0.29	−0.15	0.005	−0.18	−0.25	−0.12	**3e−04**
**Subiculum**	−0.22	−0.28	−0.15	0.0058	−0.16	−0.23	−0.1	**0.0015**
**CA1**	−0.24	−0.3	−0.17	**0.0026**	−0.2	−0.27	−0.14	**9.5e−05**
**Hippocampal fissure**	−0.08	−0.15	−0.02	0.2861	0.04	−0.03	0.1	0.4743
**Presubiculum**	−0.22	−0.29	−0.15	0.005	−0.22	−0.28	−0.15	**2.5e−05**
**Parasubiculum**	−0.16	−0.22	−0.09	0.0469	−0.05	−0.11	0.02	0.3555
**Molecular layer HP**	−0.27	−0.34	−0.2	**5e−04**	−0.24	−0.3	−0.17	**5.0e−06**
**GC ML DG**	−0.19	−0.25	−0.12	0.0177	−0.25	−0.31	−0.18	**2.0e−06**
**CA2/3**	−0.14	−0.21	−0.08	0.0639	−0.11	−0.18	−0.05	0.0306
**CA4**	−0.17	−0.24	−0.1	0.0307	−0.22	−0.29	−0.16	**1.9e−05**
**Fimbria**	−0.01	−0.08	0.06	0.9206	−0.11	−0.17	−0.04	0.0375
**HATA**	−0.2	−0.27	−0.14	0.0093	−0.18	−0.25	−0.12	**4e−04**
**Whole hippocampus**	−0.27	−0.34	−0.2	**5e−04**	−0.24	−0.3	−0.17	**3.8e−06**

Notes: Significant differences indicated in bold. *Abbreviations:* CI – confidence interval; CA – cornu ammonis; GC ML DG - granule cell layer of dentate gyrus; HATA - hippocampal amygdala transition area; HP - hippocampus.

Table S19-c: Effect of antidepressant medication on hippocampal subfield volumes among bipolar disorder 1 patients (antidepressant users and non-users) with controls as reference (right hemisphere).

	**Antidepressant users**				**Antidepressant non-users**			
**Structure**	**Cohen's d**	**Lower CI**	**Upper CI**	**p-value**	**Cohen's d**	**Lower CI**	**Upper CI**	**p-value**
**Hippocampal tail**	−0.17	−0.24	−0.1	0.0272	−0.14	−0.21	−0.08	0.0056
**Subiculum**	−0.21	−0.28	−0.15	0.0061	−0.15	−0.22	−0.09	**0.0028**
**CA1**	−0.24	−0.31	−0.18	**0.0018**	−0.22	−0.29	−0.16	**2.0e−05**
**Hippocampal fissure**	0.02	−0.05	0.09	0.7933	0.15	0.08	0.21	0.0048
**Presubiculum**	−0.2	−0.27	−0.13	0.0104	−0.22	−0.28	−0.15	**3.0e−05**
**Parasubiculum**	−0.1	−0.17	−0.03	0.1976	−0.1	−0.16	−0.03	0.0556
**Molecular layer HP**	−0.3	−0.37	−0.23	**1e−04**	−0.26	−0.33	−0.2	**4.0e−07**
**GC ML DG**	−0.3	−0.37	−0.23	**1e−04**	−0.26	−0.32	−0.19	**7.1e−07**
**CA2/3**	−0.24	−0.31	−0.17	**0.0023**	−0.15	−0.21	−0.08	0.0046
**CA4**	−0.29	−0.36	−0.22	**2e−04**	−0.23	−0.29	−0.16	**9.7e−06**
**Fimbria**	0	−0.06	0.07	0.9687	−0.09	−0.16	−0.03	0.0658
**HATA**	−0.11	−0.18	−0.04	0.1609	−0.15	−0.22	−0.09	**0.0028**
**Whole hippocampus**	−0.29	−0.36	−0.22	**2e−04**	−0.25	−0.32	−0.19	**1.2e−06**

Notes: Significant structures indicated in bold. *Abbreviations:* CI – confidence interval; CA – cornu ammonis; GC ML DG - granule cell layer of dentate gyrus; HATA - hippocampal amygdala transition area; HP - hippocampus.

Table S20-a: Effect of lithium, antipsychotic, antidepressant, and antiepileptic medication among bipolar disorder 1 patients (reference non-user of the respective medication; both hemispheres combined).

	**Lithium users**				**Antipsychotic users**				**Antidepressant users**				**Antiepileptic user**			
**Structure**	**Cohen's d**	**Lower CI**	**Upper CI**	**p-value**	**Cohen's d**	**Lower CI**	**Upper CI**	**p-value**	**Cohen's d**	**Lower CI**	**Upper CI**	**p-value**	**Cohen's d**	**Lower CI**	**Upper CI**	**p-value**
**Hippocampal tail**	0.04	−0.13	0.2	0.6902	−0.06	−0.22	0.11	0.5075	−0.03	−0.2	0.13	0.7341	−0.23	−0.4	−0.07	0.0075
**Subiculum**	0.02	−0.14	0.19	0.7869	−0.12	−0.29	0.04	0.1659	−0.04	−0.21	0.12	0.6633	−0.15	−0.31	0.02	0.0926
**CA1**	0.18	0.02	0.35	0.0498	−0.08	−0.25	0.08	0.338	0.01	−0.16	0.17	0.9418	0.01	−0.15	0.18	0.8813
**Hippocampal fissure**	−0.21	−0.37	−0.04	0.0264	0.22	0.05	0.38	0.0142	−0.13	−0.3	0.03	0.1678	0.05	−0.12	0.21	0.5799
**Presubiculum**	0.13	−0.04	0.29	0.1646	−0.28	−0.44	−0.11	**0.0017**	0.02	−0.15	0.18	0.8452	−0.09	−0.26	0.07	0.2862
**Parasubiculum**	0	−0.17	0.16	0.9804	−0.22	−0.39	−0.06	0.0116	−0.07	−0.23	0.1	0.4854	0.04	−0.12	0.21	0.6377
**Molecular layer HP**	0.19	0.02	0.35	0.0403	−0.16	−0.33	0	0.0682	0	−0.16	0.17	0.9857	−0.05	−0.21	0.12	0.5805
**GC ML DG**	0.19	0.03	0.36	0.0387	−0.11	−0.28	0.05	0.2047	0.06	−0.11	0.22	0.5672	0.02	−0.15	0.18	0.8276
**CA3**	0.17	0.01	0.34	0.0642	−0.02	−0.19	0.14	0.8142	−0.05	−0.22	0.11	0.6005	0.11	−0.06	0.27	0.2189
**CA4**	0.17	0.01	0.34	0.0632	−0.06	−0.23	0.1	0.4648	0.04	−0.13	0.2	0.7037	0.03	−0.14	0.19	0.7317
**Fimbria**	0.09	−0.08	0.25	0.3304	−0.22	−0.39	−0.06	0.0111	0.12	−0.05	0.28	0.216	−0.02	−0.19	0.14	0.8049
**HATA**	0.19	0.03	0.36	0.0369	−0.14	−0.3	0.03	0.1224	0.04	−0.12	0.21	0.6588	0.01	−0.16	0.17	0.9159
**Whole hippocampus**	0.16	0	0.33	0.0813	−0.16	−0.33	0	0.065	0	−0.17	0.16	0.9615	−0.07	−0.23	0.1	0.4395

Notes: Significant structures indicated in bold. *Abbreviations:* CI – confidence interval; CA – cornu ammonis; GC ML DG - granule cell layer of dentate gyrus; HATA - hippocampal amygdala transition area; HP - hippocampus.

Table S20-b: Effect of lithium, antipsychotic, antidepressant, and antiepileptic medication among bipolar disorder 1 patients (reference non-user of the respective medication; left hemisphere).

	Lithium users				Antipsychotic users				Antidepressant users				Antiepileptic users			
**Structure**	**Cohen's d**	**Lower CI**	**Upper CI**	**p-value**	**Cohen's d**	**Lower CI**	**Upper CI**	**p-value**	**Cohen's d**	**Lower CI**	**Upper CI**	**p-value**	**Cohen's d**	**Lower CI**	**Upper CI**	**p-value**
**Hippocampal tail**	0.06	−0.11	0.22	0.5179	−0.1	−0.27	0.06	0.249	−0.03	−0.19	0.14	0.786	−0.19	−0.35	−0.02	0.0288
**Subiculum**	−0.03	−0.19	0.14	0.7842	−0.12	−0.29	0.04	0.1692	−0.03	−0.2	0.13	0.7475	−0.18	−0.35	−0.02	0.0351
**CA1**	0.17	0	0.33	0.0682	−0.06	−0.22	0.11	0.5168	−0.01	−0.17	0.16	0.928	0.01	−0.16	0.17	0.9453
**Hippocampal fissure**	−0.19	−0.35	−0.02	0.0439	0.15	−0.02	0.31	0.0968	−0.12	−0.28	0.05	0.2179	−0.02	−0.19	0.14	0.8058
**Presubiculum**	0.08	−0.09	0.25	0.3857	−0.19	−0.36	−0.03	0.027	−0.01	−0.18	0.15	0.893	−0.1	−0.26	0.07	0.258
**Parasubiculum**	−0.01	−0.17	0.16	0.9402	−0.2	−0.36	−0.03	0.0251	−0.11	−0.28	0.05	0.2499	0.07	−0.09	0.24	0.4104
**Molecular layer HP**	0.16	−0.01	0.32	0.0844	−0.14	−0.3	0.03	0.1187	0	−0.17	0.16	0.9778	−0.07	−0.23	0.1	0.4464
**GC ML DG**	0.17	0.01	0.34	0.0609	−0.11	−0.27	0.06	0.2179	0.11	−0.06	0.27	0.2603	−0.01	−0.17	0.16	0.913
**CA3**	0.19	0.02	0.35	0.0429	−0.04	−0.21	0.12	0.6462	0	−0.17	0.16	0.9635	0.08	−0.09	0.24	0.3682
**CA4**	0.15	−0.01	0.32	0.1007	−0.07	−0.23	0.1	0.4353	0.1	−0.07	0.27	0.2993	0	−0.16	0.17	0.9701
**Fimbria**	0.08	−0.09	0.24	0.4094	−0.17	−0.33	0	0.0556	0.1	−0.07	0.26	0.3175	0.02	−0.15	0.18	0.8189
**HATA**	0.18	0.02	0.35	0.0458	−0.11	−0.28	0.05	0.2061	0	−0.16	0.17	0.9903	−0.03	−0.2	0.13	0.6984
**Whole hippocampus**	0.13	−0.03	0.3	0.1466	−0.17	−0.33	0	0.0561	−0.01	−0.17	0.16	0.9504	−0.07	−0.24	0.09	0.4073

Notes: Significant structures indicated in bold. *Abbreviations:* CI – confidence interval; CA – cornu ammonis; GC ML DG - granule cell layer of dentate gyrus; HATA - hippocampal amygdala transition area; HP - hippocampus.

Table S20-c: Effect of lithium, antipsychotic, antidepressant, and antiepileptic medication among bipolar disorder 1 patients (reference non-user of the respective medication; right hemisphere).

	Lithium users				Antipsychotic users				Antidepressant users				Antiepileptic users			
**Structure**	**Cohen's d**	**Lower CI**	**Upper CI**	**p-value**	**Cohen's d**	**Lower CI**	**Upper CI**	**p-value**	**Cohen's d**	**Lower CI**	**Upper CI**	**p-value**	**Cohen's d**	**Lower CI**	**Upper CI**	**p-value**
**Hippocampal tail**	0.01	−0.16	0.17	0.9402	0	−0.17	0.16	0.9813	−0.04	−0.2	0.13	0.7091	−0.24	−0.41	−0.07	0.0055
**Subiculum**	0.08	−0.08	0.25	0.3735	−0.11	−0.27	0.06	0.2285	−0.05	−0.21	0.12	0.6396	−0.08	−0.25	0.08	0.3277
**CA1**	0.17	0	0.34	0.0659	−0.1	−0.26	0.07	0.2578	0.02	−0.14	0.19	0.8248	0.02	−0.15	0.18	0.8398
**Hippocampal fissure**	−0.18	−0.34	−0.01	0.0554	0.24	0.08	0.41	0.0057	−0.13	−0.29	0.04	0.1903	0.11	−0.05	0.28	0.2006
**Presubiculum**	0.15	−0.02	0.31	0.1075	−0.31	−0.48	−0.15	**4e−04**	0.05	−0.12	0.21	0.6095	−0.07	−0.24	0.09	0.3917
**Parasubiculum**	0	−0.17	0.16	0.9849	−0.19	−0.35	−0.02	0.0324	0.01	−0.16	0.17	0.941	−0.02	−0.18	0.15	0.8395
**Molecular layer HP**	0.2	0.03	0.36	0.0334	−0.16	−0.33	0	0.0637	0.01	−0.16	0.17	0.9503	−0.02	−0.19	0.14	0.7911
**GC ML DG**	0.18	0.02	0.35	0.0496	−0.1	−0.26	0.07	0.2672	−0.01	−0.17	0.16	0.9478	0.05	−0.12	0.21	0.5959
**CA3**	0.13	−0.04	0.29	0.1655	0	−0.17	0.17	0.9997	−0.08	−0.25	0.08	0.3865	0.12	−0.05	0.28	0.1781
**CA4**	0.17	0	0.33	0.0717	−0.05	−0.21	0.12	0.5724	−0.03	−0.2	0.13	0.7458	0.05	−0.11	0.22	0.5348
**Fimbria**	0.08	−0.09	0.24	0.392	−0.23	−0.39	−0.06	0.0103	0.12	−0.05	0.28	0.2302	−0.06	−0.22	0.11	0.4983
**HATA**	0.16	0	0.33	0.078	−0.14	−0.3	0.03	0.1214	0.08	−0.09	0.25	0.4058	0.05	−0.11	0.22	0.5275
**Whole hippocampus**	0.17	0.01	0.34	0.064	−0.14	−0.31	0.02	0.1034	0	−0.17	0.16	0.9609	−0.05	−0.22	0.11	0.5347

Notes: Significant structures indicated in bold. *Abbreviations:* CI – confidence interval; CA – cornu ammonis; GC ML DG - granule cell layer of dentate gyrus; HATA - hippocampal amygdala transition area; HP - hippocampus.

Supplemental Notes

Note S1: Quality control procedure

The goal of the quality control (QC) was to identify subjects where the hippocampal subfield segmentation had failed. For each site, we computed a QC list of outlying structures for the total hippocampal and subfield volumes, bilaterally, using standardized quality control scripts. This list consisted of subjects where one or more hippocampal volumes were either outliers or showed rank violations. Outliers were defined as falling outside the range [Q1 - 1.5*IQR, Q3 + 1.5*IQR] where Q1 and Q3 are the first and third quartiles and IQR is the interquartile range, which assuming a normal distribution is equivalent to measurements more than 2.698 standard deviations (SD) from the mean. Rank violations were defined as either unusually low hippocampal tail volume, the subiculum not being ranked as the fourth largest subfield, or CA1 not being ranked as the largest subfield.

For each subject in the QC list, the segmentation quality was visually inspected, and subjects were excluded if the segmentation was inaccurate. Given the difficulty in visual inspection of individual subfields, the most useful indications that segmentation had failed were the placements of the overall hippocampal mask and the hippocampal fissure. Below is a figure showing an acceptable segmentation (left) despite the presence of a hole in the hippocampal fissure label (purple label, purple arrow) and an unacceptable segmentation (right) due to the presence of a hole in the CA1 label (red label, red arrow) leading to subject exclusion.

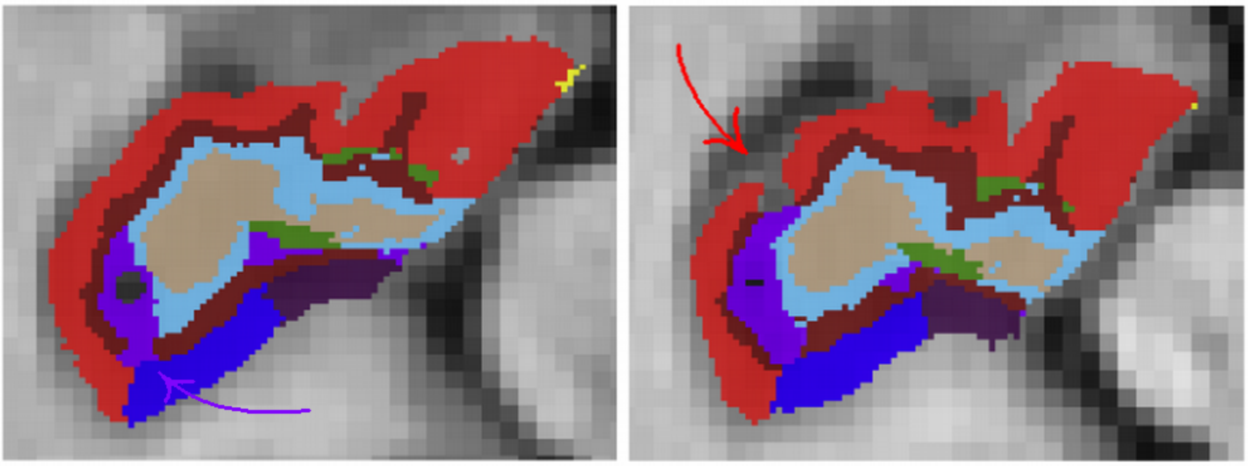

For detailed documentation of the quality control procedure see: https://pgc-ptsd.com/wp-content/uploads/2017/08/PTSD_Instructions_Subfields_part_IR_II.pdf.

Note S2: Statistical analyses

We used the Wilcoxon rank-sum test, also referred to as the Mann-Whitney U test, which is a nonparametric alternative to the t-test for two independent samples that does not assume normality. It is a nonparametric method that uses ranks and not the actual observed value, and computes the cumulative distribution functions of ranks

prior to finding the median shift *Δ* between the two groups. If *Δ*=0 the cumulative distribution functions do not differ between the groups [Rosner, 2011]. We used the *wilcox.test*-function in R (version 3.5.2, www.R-project.org) to extract the estimated median shift (*Δ*) and the corresponding p-value.

Note S3: Forest plots

To generate the forest plots, we used R (version 3.5.2, www.R-project.org). Firstly, we applied linear regression (*lm-function)* for each hippocampal subfield structure (dependent variable) with diagnosis, age, age^2, sex, age*sex, and sex*age^2, and ICV (covariates) and computed Cohen's d effect size and its standard error [Nakagawa and Cuthill, 2007] for diagnosis (the covariate of interest) site-/scanner-wise. Secondly, we performed a meta-analysis for each combined structure by pooling the Cohen's d effect sizes and standard errors from each site using an inverse variance-weighted random-effects model, fitted by a restricted maximum-likelihood estimator, by using the *rma-function* from the *metafor*-package (version 2.0.0) [Viechtbauer, 2010]. Thirdly, forest plots were generated using the *forest*-function from the *metafor*-package, providing the output from the *rma*-function as input.

References

Dannlowski U, Grabe HJ, Wittfeld K, Klaus J, Konrad C, Grotegerd D, Redlich R, Suslow T, Opel N, Ohrmann P, Bauer J, Zwanzger P, Laeger I, Hohoff C, Arolt V, Heindel W, Deppe M, Domschke K, Hegenscheid K, Völzke H, Stacey D, Meyer zu Schwabedissen H, Kugel H, Baune BT (2015): Multimodal imaging of a tescalcin (*TESC*)-regulating polymorphism (rs7294919)-specific effects on hippocampal gray matter structure. Mol Psychiatry 20:398–404. https://www.nature.com/articles/mp201439.

Gurholt TP, Osnes K, Nerhus M, Jørgensen KN, Lonning V, Berg AO, Andreassen OA, Melle I, Agartz I (2018): Vitamin D, Folate and the Intracranial Volume in Schizophrenia and Bipolar Disorder and Healthy Controls. Sci Rep 8:10817.

Haarman BCM "Benno," Burger H, Doorduin J, Renken RJ, Sibeijn-Kuiper AJ, Marsman J-BC, de Vries EFJ, de Groot JC, Drexhage HA, Mendes R, Nolen WA, Riemersma-Van der Lek RF (2016): Volume, metabolites and neuroinflammation of the hippocampus in bipolar disorder - A combined magnetic resonance imaging and positron emission tomography study. Brain Behav Immun 56:21–33.

Haukvik UK, Westlye LT, Morch-Johnsen L, Jorgensen KN, Lange EH, Dale AM, Melle I, Andreassen OA, Agartz I (2015): In vivo hippocampal subfield volumes in schizophrenia and bipolar disorder. Biol Psychiatry 77:581–8.

van der Kouwe AJW, Benner T, Salat DH, Fischl B (2008): Brain morphometry with multiecho MPRAGE. NeuroImage 40:559–569.

Nakagawa S, Cuthill IC (2007): Effect size, confidence interval and statistical significance: a practical guide for biologists. Biol Rev Camb Philos Soc 82:591–605.

Roberts G, Lenroot R, Frankland A, Yeung PK, Gale N, Wright A, Lau P, Levy F, Wen W, Mitchell PB (2016): Abnormalities in left inferior frontal gyral thickness and parahippocampal gyral volume in young people at high genetic risk for bipolar disorder. Psychol Med 46:2083–2096.

Rosner B (2011): Fundamentals of biostatistics. Boston: Brooks/Cole, Cengage Learning.

Soeiro-de-Souza MG, Lafer B, Moreno RA, Nery FG, Chile T, Chaim K, Leite C da C, Machado-Vieira R, Otaduy MCG, Vallada H (2017): The CACNA1C risk allele rs1006737 is associated with age-related prefrontal cortical thinning in bipolar I disorder. Transl Psychiatry 7:e1086–e1086. https://www.nature.com/articles/tp201757.

Sprooten E, Sussmann JE, Clugston A, Peel A, McKirdy J, Moorhead TWJ, Anderson S, Shand AJ, Giles S, Bastin ME, Hall J, Johnstone EC, Lawrie SM, McIntosh AM (2011): White matter integrity in individuals at high genetic risk of bipolar disorder. Biol Psychiatry 70:350–356.

Tesli N, van der Meer D, Rokicki J, Storvestre G, Røsæg C, Jensen A, Hjell G, Bell C, Fischer-Vieler T, Tesli M, Andreassen OA, Melle I, Agartz I, Haukvik UK (2020): Hippocampal subfield and amygdala nuclei volumes in schizophrenia patients with a history of violence. Eur Arch Psychiatry Clin Neurosci.

Van Gestel H, Franke K, Petite J, Slaney C, Garnham J, Helmick C, Johnson K, Uher R, Alda M, Hajek T (2019): Brain age in bipolar disorders: Effects of lithium treatment. Aust N Z J Psychiatry:4867419857814.

Viechtbauer W (2010): Conducting Meta-Analyses in R with the metafor Package. J Stat Softw 36:1–48.

Vogelbacher C, Möbius TWD, Sommer J, Schuster V, Dannlowski U, Kircher T, Dempfle A, Jansen A, Bopp MHA (2018): The Marburg-Münster Affective Disorders Cohort Study (MACS): A quality assurance protocol for MR neuroimaging data. NeuroImage 172:450–460. http://www.sciencedirect.com/science/article/pii/S105381191830079X.
